# Valence activity of SO-coupled atomic core shells in solid compounds of heavy elements†

**DOI:** 10.1039/d4sc08151j

**Published:** 2025-02-21

**Authors:** Shi-Ru Wei, Han-Shi Hu, W. H. Eugen Schwarz, Jun Li

**Affiliations:** a Theoretical Chemistry Center, Department of Chemistry, Engineering Research Center of Advanced Rare-Earth Materials of the Ministry of Education, Tsinghua University Beijing 100084 China junli@tsinghua.edu.cn; b Physical and Theoretical Chemistry Lab, Department of Chemistry and Biology, Faculty of Science and Technology, University of Siegen Siegen 57068 Germany eugen.schwarz@uni-siegen.de; c Department of Chemistry, Southern University of Science and Technology Shenzhen 518055 China; d Fundamental Science Center of Rare Earths, Ganjiang Innovation Academy, Chinese Academy of Sciences Ganzhou 341000 China

## Abstract

A close inspection reveals chemically relevant changes from light to heavy elements of the atomic orbital-energy patterns, relevant for both chemical theory and material applications. We have quantum-chemically investigated the geometric and electronic structures of solid [ThO_2_] and a series of [UO_3_] phases at a realistic relativistic level, both with and without spin–orbit (SO) coupling. The observable band gap between the occupied O(2p) bonding valence band and the empty U(5f6d) conduction band is smallest for δ-[UO_3_], with medium short U–O distances and high *O*_h_ symmetry. Both Pauli-repulsion of O(2p) by the strongly SO-split U(6p) core and additional covalent U(6p)–O(2p) mixing cause a “pushing up from below” (PFB) and a large SO splitting of the valence band of the light element. PFB has been observed in molecular chemistry, but PFB and PFB-induced SO splitting have so far not been considered in solid-state science. Our findings open up new possibilities for electronic material applications.

## Abstract 2

The core–valence energy gaps decrease from the light to the heavy elements, with the heavy noble-gas cores transferring their large spin–orbit splitting into the upwardly pushed valence bands of the ligands.

## Graphical abstract

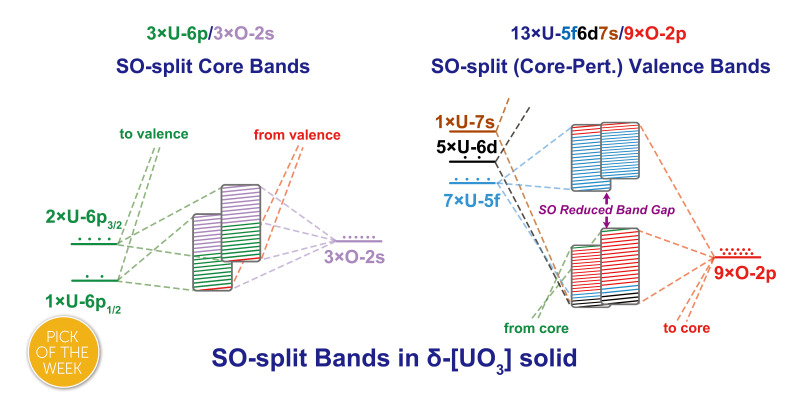

## Introduction

Solid actinide materials are very important in the nuclear fuel cycle, as niche products for special applications in catalysis and nano-chemistry, and also for the understanding of the natural system of elements. However, experimental research on actinides has become limited. Several uranium oxides [UO_*x*_] of different oxidation states (*x* from 2 to 3) have been studied for a long time.^1–3^ Theoretical calculations are particularly important, though they are particularly challenging due to the extended spdf valence shell, the strong electron correlation, and the relativistic effects including spin–orbit (SO) coupling.^4^ The electrons in chemical compounds can be classified by their energies, either as weakly bound and chemically active “valence” electrons, or as more strongly bound and chemically inactive “core” electrons. However, actinide chemistry breaks several common rules of chemistry and opens up new horizons due to the core-penetrating valence shell with a non-innocent outer core shell.^5^

The atoms of the light and medium heavy elements have closed (s)^2^, (sp)^8^ or (spd)^18^ core shells and (sp)^*x*^ or (ds)^*x*^ valence shells, with large core–valence (c–v) gaps in between. These s-, sp- and d-elements are well investigated, both in theory and experiment. For the heaviest elements, however, there are more levels per energy unit (high density of states) and the c–v gaps become smaller. Now, there is no longer a clear separation of core and valence regions.^5,6^ In 1980, Tatsumi and Hoffmann^7^ were the first to emphasize the chemical valence activity of the U-6p outer core shell. The chemical U-6p activity appears particularly significant for 5f^0^ systems. In 1982 Jørgensen baptized this 6p semi-core effect as “pushing from below” (PFB) into the valence shell.^8^ Respective molecular reviews by Bursten^9^ and Denning^10^ appeared in the early 1990s. Pyykkö even counted the U-6p semi-core shell of UO_6_ molecules fully among the valence shells.^11^ More recent works^12–17^ supported the molecular PFB effect, and also reviewed the inverse *trans* influence (ITI) in uranyl analogues, and stressed the multi-centre bonding character of many actinide molecules. Apparently however, the possibility of PFB in solid actinide compounds, and in particular the connected and chemically important spin–orbit coupling in solids has never been considered in detail. Therefore we here investigate solid [Th(iv)O_2_] and [U(vi)O_3_] phases, where some former literature has largely neglected the semi-core shell activity and the respective SO effects.^18–20^

There are two main challenges in the theoretical calculation of actinide solids. The first is the strong correlation problem of the extended 5f6d7sp valence–shell, still not easily handled by extended wave-function (WF) nor by single-determinant density-functional (DF) approaches. The latter ones also suffer from the self-interaction error,^21,22^ localization^23^ and delocalization errors.^21^ The dative pairs of the coordination bonds often become symmetry-broken with some orbital components polarized more towards the central metal, and others more towards the ligands.^23^ Another issue is the sometimes comparatively small covalent overlap of the d and f orbitals of the transition metal atoms, causing charge-transfer resonance and spin-decoupling effects.^24–26^ Therefore, various computational approaches beyond the pure density and density-gradient approaches have been applied for a better geometric and electronic structure reproduction of the actinide solids (for more details see the ESI, Section 1†).

The second challenge of actinide quantum chemistry is the strongly relativistic behaviour of the valence electrons, especially their SO coupling. Complex two-component (quaternionic) spinors instead of the one-component real spin–orbitals are a challenge for both computation and analysis. Many authors are convinced that SO coupling is of little relevance for geometric structure and thermodynamic and reactive energies of high-valent actinide compounds, because the valence band is dominated by the light-atomic ligands, and relativistic effects are believed to play only a role in electronic excitations into the empty actinide (5f6d)^0^ shells with medium strong SO splitting (up to 1 eV). However, SO coupling can become particularly important for p-type electronic structures. Huhn & Blum's benchmark work^27^ on compounds of the heavy 6p elements (Tl, Pb, Bi, Po) indicates huge band-structure changes due to SO coupling (see also ref. 28). The SO coupling is proportional to 
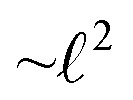
, but contains a radial pre-factor 
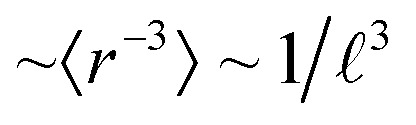
. In a given range of energies, the SO splitting is therefore larger for smaller angular momentum values 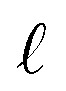.^29^ Another widespread misconception since Dirac's early days is the belief that one-electron states in a screened Coulomb potential with small total orbital energy behave largely non-relativistic. However, both the orthogonality of the valence orbitals to the strongly relativistic core shells as well as the direct action of the relativistic Hamiltonian on the inner tails of the ‘deep diving’ s and p orbitals induce significant relativistic effects in the valence shell around a heavy nucleus.^30^

The chemical bonding in polar actinide compounds with Lewis bases is dominantly due to donation of ligand electron pairs into the An-5f6d valence band. The respective medium strong SO coupling in actinide oxides such as [UO_2_], [U_3_O_8_], [NpO_2_], [PuO_2_] *etc.*, partially quenched by the crystal fields, has often been explored, sometimes with diverging conclusions.^31–38^ Depending on the specific system and the property of concern, SO coupling can sometimes be neglected, whereby the wave-function computation and analysis becomes much easier and faster. This experience has mistakenly led previous researchers of [UO_3_] phases (with formal An-5f^0^ configuration) to refrain from the consideration of SO influences altogether.

## Materials and methods

Quantum chemical calculations on materials were performed with software packages AMS-BAND^39–43^ and VASP^44–47^ for solids and AMS-ADF ^48–50^ for molecules, applying the (more critical) independent-electron and density-functional (DF) approximations, and the (less critical) zero-order regular approximation (ZORA) for relativity including spin–orbit coupling (SOC). The bonding analyses were performed with the help of LOBSTER.^51,52^ More details are given in the ESI, Section 2.†

## Results

### Geometric structure

Geometric structure data (computed by the PBE density functional approach) of one [ThO_2_] and five [UO_3_] phases are summarized in the ESI, Tables S2, S4 and Fig. S1.† The optimized structures agree well with the experimental results at the % level (Table S2†). Concerning α-[UO_3_], different experimental structure data are found in the literature.^53–55^ We find the *C*2*mm* structure to be unstable, as also noted by Brincat,^56^ and the *C*2 and *P*3̄*m*1 structures to be very similar (Table S3†). Therefore, in the following, we consider only α-*P*3̄*m*1. Concerning γ-[UO_3_], we also find two very similar structures *Fddd* and *I*4_1_.

**Fig. 1:**
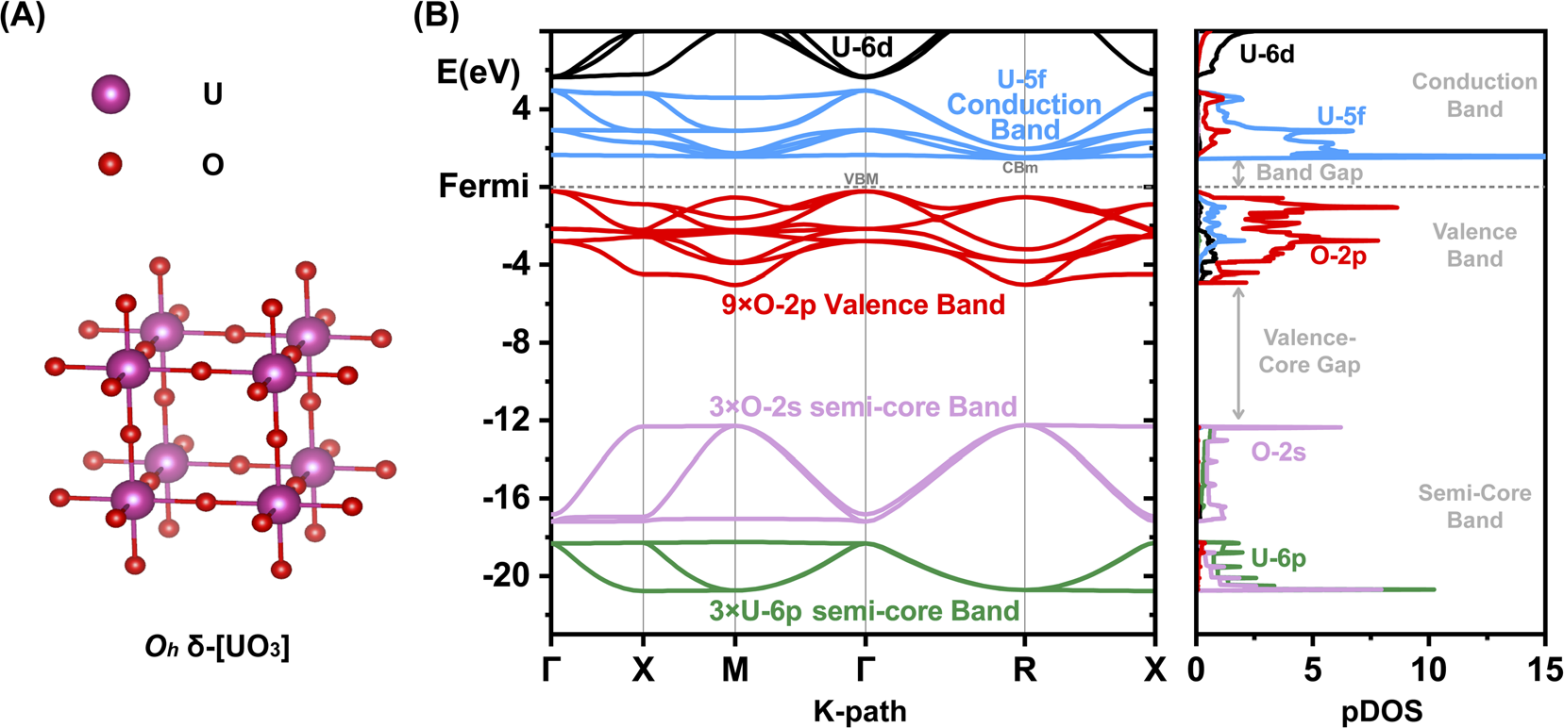
Geometric and electronic structure of δ-[UO_3_]. (A) Geometric structure. (B) Electronic band structure and partial atomic orbital density of states (pDOS). Energy range of semi-core U-6p (green) and O-2s (lilac), and of valence O-2p (red), U-5f (blue) and U-6d (black). SR Kohn–Sham PBE approximation; energies in eV w. r. t. the lower Fermi edge.

Most [UO_3_] polymorphs have linear chains of more or less strongly, ‘triply’ bonded uranylic units (U–O ∼1.8–2.0 Å), weakly connected by equatorial flat or puckered oxygen layers (U–O ∼2¼ − 2½ Å). Long and short U–O distances with nearly constant mean values had been discussed by Pyykkö.^57^ Only the α- and δ-[UO_3_] phases have σπ-bonded –O–U–O–U– strings in all directions: the α-phase has slightly expanded and contracted distances of 2.1 and 2.2 Å, and the δ-phase is fully symmetric in all 3 directions with 6 equal U–O distances of 2.08 Å (Fig. 1A). The six O atoms around the U atom generate a perfect *O*_h_ crystal field (CF), which facilitates the transfer of strong U-6p SO splitting into the O-2p valence band. In the other [UO_3_] phases, in contrast, the SO splitting is partially quenched by the distorted CF due to 2 short axial and 4 more distant equatorial O atoms. The mutual interaction of the CF and SO coupling is discussed below.

### Large band gap reductions in [UO_3_] phases by SO splitting

An overview of the chemically relevant spin-averaged scalar relativistic (SR) band structure and the partial density of states (pDOS) of δ-[UO_3_] is displayed in Fig. 1B, covering the O-2p and U-5f,6d valence shells around the Fermi level, and also the often-overlooked “semi-core” bands below. Textbooks usually consider O-2s^2^ as a valence shell, while its low energy is near-degenerate with the U-6p^6^ shell, which is commonly viewed as a chemically inert noble-gas atomic-core shell. Below we will account for relativistic U-6p spin–orbit coupling, which causes: (i) small energy shifts of the O-2p shell, (ii) effects of the order of up to 1 eV in the U valence shells, and (iii) splitting of up to 10 eV between U-6p_1/2_ and U-6p_3/2_ (see Tables S13 and 14†). This has unexpected effects for various actinide compounds in their band structure around the Fermi level, which will be elucidated below.

We compare the computationally and experimentally derived band gaps of one [ThO_2_] and five [UO_3_] phases in Table 1 (see also Table S5†). Overall, the PBE density and gradient functional approximation provides far too small band gaps, especially if SOC is taken into accounted correctly (errors of almost −1 and −1½ eV, respectively). In the present cases, the empirical Hubbard+*U* correction for 2-electron interactions accidently yields satisfactory band gaps provided SOC is neglected. This indicates the inadequacy of the PBE+*U* approach, but was sometimes taken as an argument that SOC is insignificant. The computed band gaps increase with increasing Hubbard+*U*,^63^ but only up to +*U* ≈ 4 eV, when the crystal orbitals become ‘rearranged’. Several common density functionals reproduce reasonable band gaps only for some of the oxide phases, with an overall error scattering of the order of ±1 eV.

**Table 1:** Calculated (VASP) band gaps (in eV) of several [An(5f^0^)O_*n*_] phases, at various density functional levels (PBE, PBE+*U*, HSE, *G*_0_*W*_0_@PBE), at the scalar-relativistic approximation and with spin–orbit coupling (+SOC). At the bottom the experimentally derived values (Exptl.) for comparison, and the reduction of the band gap due to SOC (Δ_SOC_Δ*E*_gap_)

	Band gap (Δ*E*_gap_ in eV)					
Solid phase	δ-[UO_3_]	α-[UO_3_]	β-[UO_3_]	γ-[UO_3_]	η-[UO_3_]	[ThO_2_]
Space group	*Pm*3̄*m*	*P*3̄*m*1	*P*2_1_	*Fddd*	*P*2_1_2_1_2_1_	*Fm*3̄*m*
**PBE**	1.67	1.68	1.44	1.89	1.91	4.45
PBE + SOC	0.75	1.31	0.98	1.46	1.38	4.35
**PBE+** ***U***	2.25	1.97	2.34	2.79	2.70	4.83
PBE+*U* + SOC	1.26	1.49	1.77	2.37	2.13	4.74
**HSE**	3.26	2.96	3.14	3.68	3.60	6.15
HSE + SOC	2.28	2.42	2.61	3.23	3.05	6.03
***G***_**0**_ ***W***_**0**_ **@PBE**	3.36	3.26	—	—	—	6.12
*G*_0_ *W*_0_@PBE + SOC	2.24	2.67	—	—	—	6.01
**Exptl.** ^1,58–62^	2.17	2.63	2.17	2.38	—	5.75–6.00
Δ_SOC_Δ*E*_gap_≈	−1.0	−0.5	−0.5	−0.4	−0.5	−0.1

**Fig. 2:**
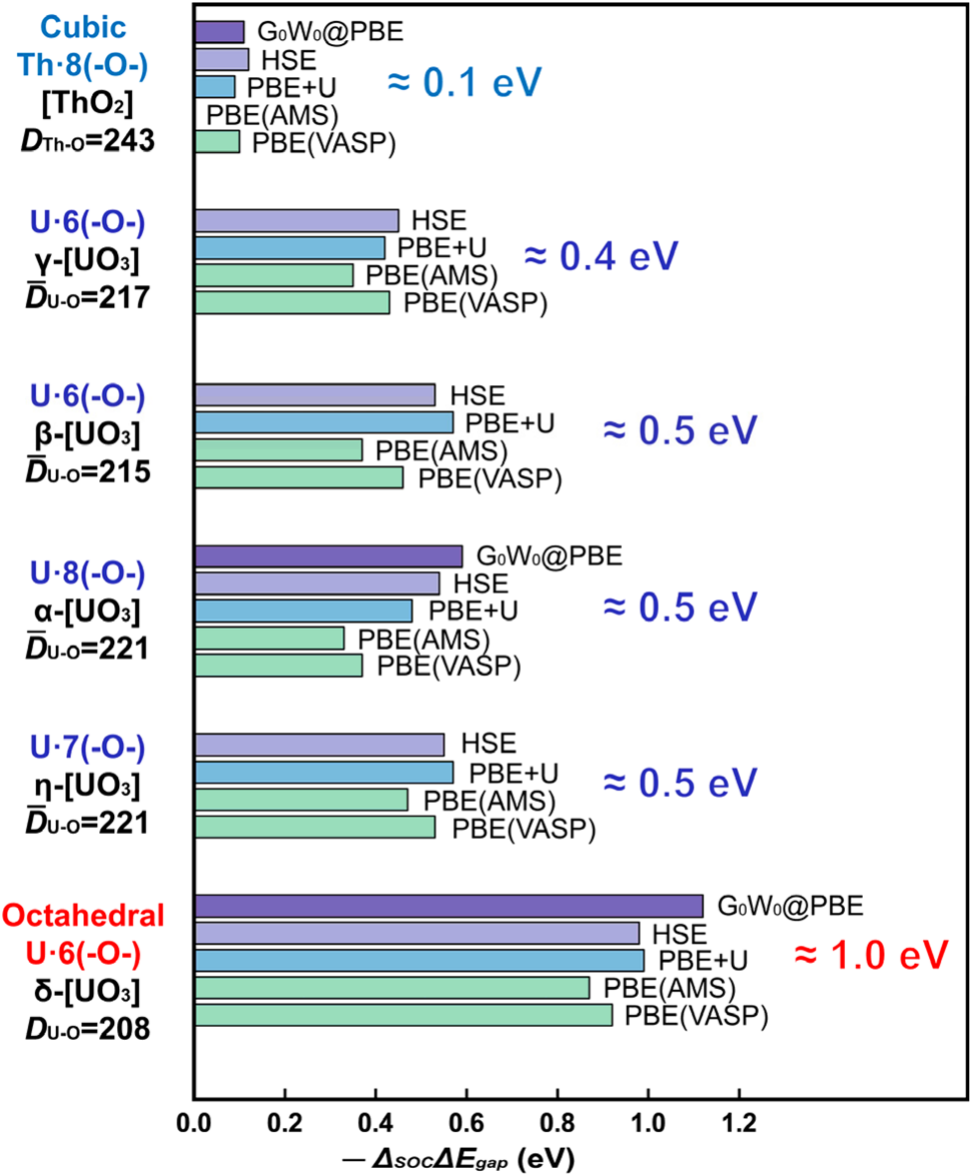
Reduction of band gap by spin–orbit coupling (SOC), w. r. t. the scalar relativistic approximation. −Δ_SOC_Δ*E*_gap_ = Δ*E*_gap_(SR) − Δ*E*_gap_(SOC) for six different An-oxide phases, calculated by PBE, PBE+*U*, HSE, and *G*_0_*W*_0_ approaches, using the VASP and AMS-BAND software. Linear (uranylic) instead of octahedral coordination perturbs the p-AO degeneracy and thus the SO coupling; longer U–O interaction distances reduce the SO splitting. Bond lengths *D*_An−O_ (average values *D̄*) in pm.

On the other hand, the HSE exchange-hybrid functional, or the *G*_0_*W*_0_ correction to PBE, both yield more physical results, meaning too large band gaps without SOC and reasonable gaps if SOC is considered for all core and valence shells. However, the (nonrelativistic) 1-electron self-interaction and 2-electron correlation still pose more serious computational problems than relativity, even including the 1-electron SOC. Table 1 and Fig. 2 show a fairly consistent band gap reduction by SOC and therefore appear reliable.

The common opinion in the literature is that the O-2p dominated valence band is hardly affected by the heavy-atomic SO coupling. Indeed, that holds for [ThO_2_] with the largest bond length. However, we here find that the gaps of most [UO_3_] phases are reduced by SO coupling by about ½ eV, but those of δ-[UO_3_] by around a surprising 1 eV. In δ-[UO_3_], SO-coupling shifts the O-2p dominated Valence Band Maximum (VBM) up by about ½ eV and lowers the U-5f Conduction Band Minimum (CBm) by about ½ eV. The energy level shift by SO coupling for all investigated [UO_3_] and [ThO_2_] phases is displayed in Table S7,† together with the % of An-*n*p mixing into the O-2p band at the VBM. Different software with different relativistic approximation schemes and using different density functionals all give the same qualitative picture (Fig. 2 and Table S6†). Even for the simplest density functional approximation, the SO coupling raises the VBM, lowers the CBm and thereby reduces the band gap of all [U(5f6d)^0^O_3_] phases. Similar trends were found in heavy atomic 6p main-group compounds.^27^

### U-6p pushing from below (PFB) in δ-[UO_3_]

**Fig. 3:**
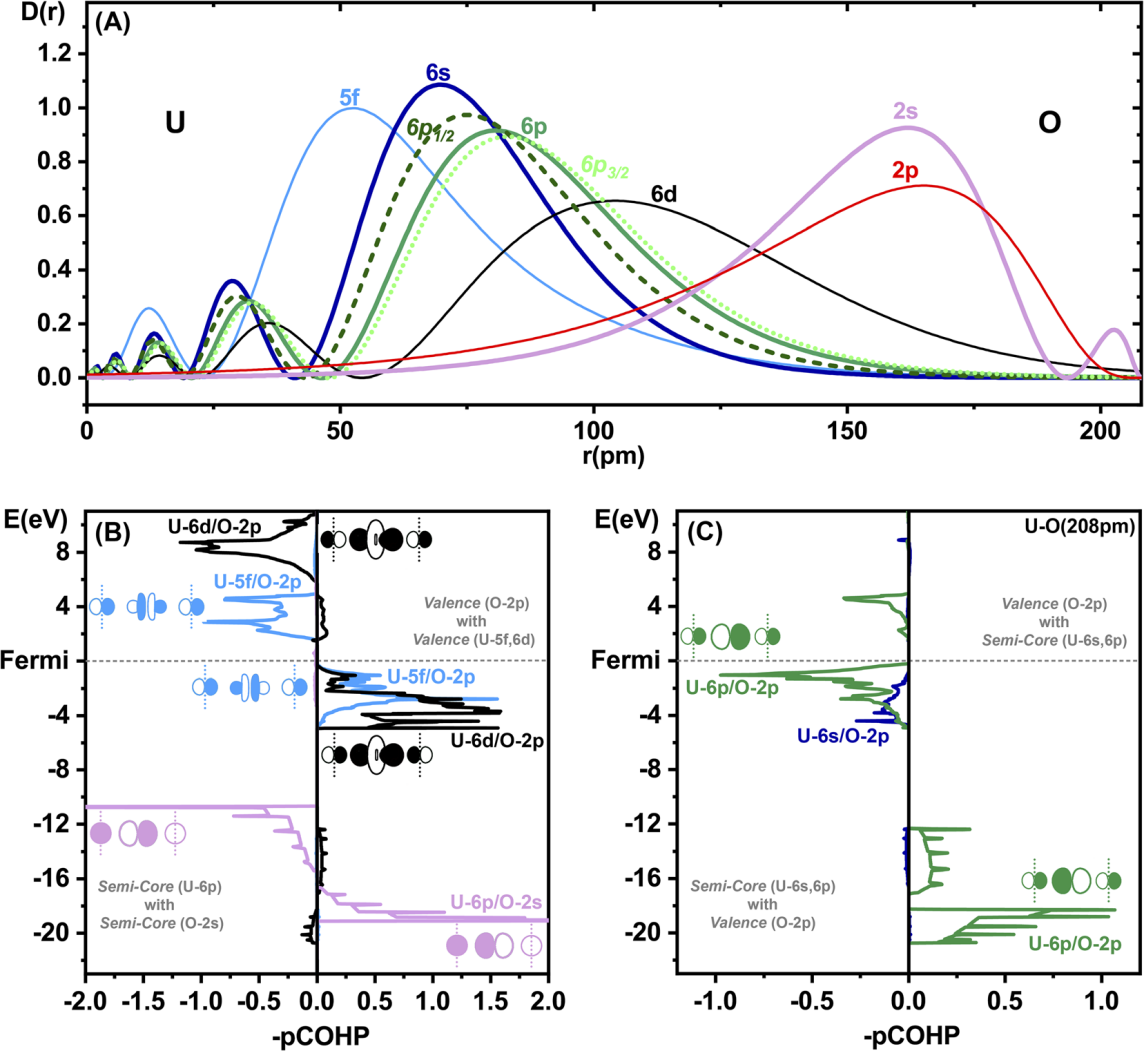
Bonding analysis of δ-[UO_3_]. (Top) (A) Radial atomic orbital density distributions *D*(*r*) (in atomic units, ZORA-PBE density functional calcs.) of U^6+^ at *r* = 0, and of O^0^ at *r* = 208 (the U–O distance in δ-[UO_3_], *r* in pm). Fully occupied semi-core orbitals are bold: U-6s (solid, dark blue), spin-averaged U-6p (solid, green), SO split U-6p_1/2_ (dashed, dark green) and U-6p_3/2_ (dotted, light green). Valence orbitals are thin: inner U-5f (blue), outer U-6d (black). Orbitals of O, in reverse: O-2s (bold, lilac) and O-2p (thin red). (Bottom) Projected crystal orbital Hamilton populations (pCOHP) of δ-[UO_3_] in the semi-core & valence regions (SR-PBE approximation) calculated with LOBSTER.^51,52^ Positive and negative values indicate, respectively, bonding and antibonding interactions, as shown by the AO sketches along an axis of the unit cell, with O atoms at the corners (vertical dashed lines) and the U atom in between. (Left) (B) At the top, the common dative valence interactions of O-2p into U-5f (blue) and U-6d (black), bonding below and antibonding above the Fermi edge; at the bottom, the ‘degeneracy-driven’ semi-core interactions due to the overlap of U-6p and O-2s (lilac). (Right) (C) The ‘unusual’ semi-core/valence interactions U-6s/O-2p (dark blue) and U-6p/O-2p (green), stabilizing the semi-core shells (at the bottom), and destabilizing (PFB) the valence levels in particular around the Fermi edge (at the top).

The SO splitting of the unperturbed atomic U-5f or U-6d valence shells is less than about 1 eV, while that of the U-6p core shell is an order of magnitude larger (Tables S13 and 14†). Hence, the “observation” for δ-[UO_3_] of a large band gap reduction by SO coupling of about 1 eV (rather independent of the chosen computational approach and of the error of the predicted gap size) indicates the admixture of core-orbitals in the valence shell and the concept of U-6p pushing from below (ESI, Section 5†). The molecular PFB has been discussed since nearly half a century.^7,8,10–15,64,65^ Motta & Autschbach had recently reviewed and computationally analysed the energetic contributions of the U-6p PFB effect in molecules.^15^ On the one hand, the binding energy of the electrons in the U-6p shell is significant in comparison to the valence orbital interaction energies of big heavy atoms. On the other hand, the radial distribution of the outer semi-core U-6p orbitals extends significantly into the valence space. The U-6p shell sits radially between the inner U-5f and outer U-6d valence shells (Fig. 3A). The radial U-6p SO splitting is very remarkable, with the U-6p_1/2_ being spatially a little larger than U-6s, and the U-6p_3/2_ a little larger than the scalar U-6p. The O-2s ‘valence’ orbital sits energetically between the SO split U-6p_1/2_ and U-6p_3/2_ ‘core’ orbitals. Both the U-6p_3/2_ Pauli repulsion (due to orbital orthogonalization) and the U-6p_3/2_ semi-core/valence activity will dominate over the weaker effects of radially smaller and lower-energy U-6p_1/2_. The more pronounced overlap-orthogonalization effects of U-6p_3/2_*vs.* U-6p_1/2_ are highlighted in Table S12.†

We study the octahedral U–O bonding in δ-[UO_3_] as a remarkable example. The projected crystal orbital Hamilton populations (pCOHP),^66,67^ calculated with LOBSTER,^51,52^ show the valence region (upper part of Fig. 3B) with common coordination bonding by O-2p pairs, donating dominantly into U-6d and also U-5f (and smaller U-7s,7p admixtures, see Fig. S5†). In the semi-core region (Fig. 3B, lower part) there are non-negligible near-degenerate attractive and repulsive U-6p/O-2s overlap interactions, almost without any overall chemical bonding (baptized degeneracy-driven bonding^68^).

Surprisingly, we see strong anti-bonding interactions of U-6p with O-2p close below the Fermi edge (green curve in Fig. 3C), with bonding counterparts in the semi-core region. Obviously, there occurs non-negligible U-6p core/O-2p valence mixing, stabilizing the U-6p core shell while pushing up some parts of the O-2p valence shell, with only a small overall bonding effect. The U-6p semi-core/O-2p valence mixing causes some population reduction of the formal U-6p^6^ shell, which remains no longer completely filled (Table S9†). This is possible because the formal O-2p^6^ shell has lost some population by the coordinative-bonding O-2p^6^→U-5f^0^6d^0^ charge transfer. In summary, the U–O bonding is due to the O valence, U valence and U semi-core shells being triply connected in an involved manner.^15^

### Ligand fields, spin–orbit coupling and core–valence mixing

Most of human chemical work concerns elements from the upper and middle parts of the periodic table, where thermodynamics and the structure of stationary compounds are mainly governed by the kinematics of charged electronic point particles in Coulomb fields. That can be represented by common real one-component wave-functions, at the non-relativistic or scalar-relativistic approximation. Spectroscopy and time-dependent reactions may require complex two-component wave-functions. Spin–orbit coupling may play a role in spectroscopy and chemical kinetics. In principle, SOC changes the fundamental symmetry and requires four-component quaternion wave-functions, making both computation and analysis up to an order of magnitude more complicated. Yet, as long as SOC is small, it can be treated as a simple perturbation in the common formalism.

However, when we come to the really heavy elements in periods 6 and 7 (including many technologically relevant metals such as Ba, the Lanthanides, Ta, W, Ir, Pt, Au, Hg, Pb, Th, U, or Pu), the common ‘scalar’ approximation is often no longer appropriate, namely if the SO-splitting becomes comparable to or greater than the chemical interactions (say of the order of an eV), as *e.g.* for the 6p 
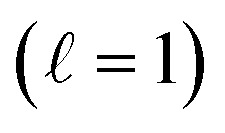
 valence shell of 6p-element Pb.^28^ A simplistic hydrogen-like model yields for the SO splitting of an 
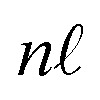
 orbital around an effective nuclear point charge:
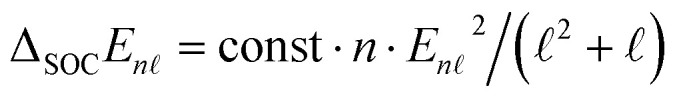


This makes it understandable that the SO splitting of the 5d, 5f and 6d 
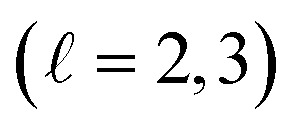
 valence shells of the mentioned heavy transition elements is not larger than 1 eV, while the SO splitting of the 5p or 6p 
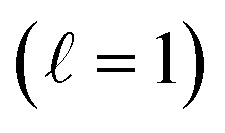
 outer core shells with larger 
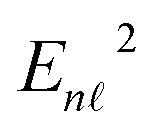
 and smaller 
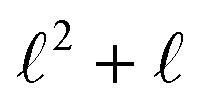
 is of the order of 10 eV.

The second point is the energy gap that separates the chemically inactive atomic core shells from the active valence shells. The large c–v gap at the upper right corner of the periodic system (O(1s–2s) ≈ 5 × 10^2^ eV, Cl(2p–3s) ≈ 2 × 10^2^ eV) shrinks dramatically toward the lower left corner (Ba(5p_3/2u_–6s_1/2g_) to Pu(6p_3/2u_–5f_5/2u_) ≈ 2 × 10^1^ eV) causing the above mentioned PFB.

**Fig. 4:**
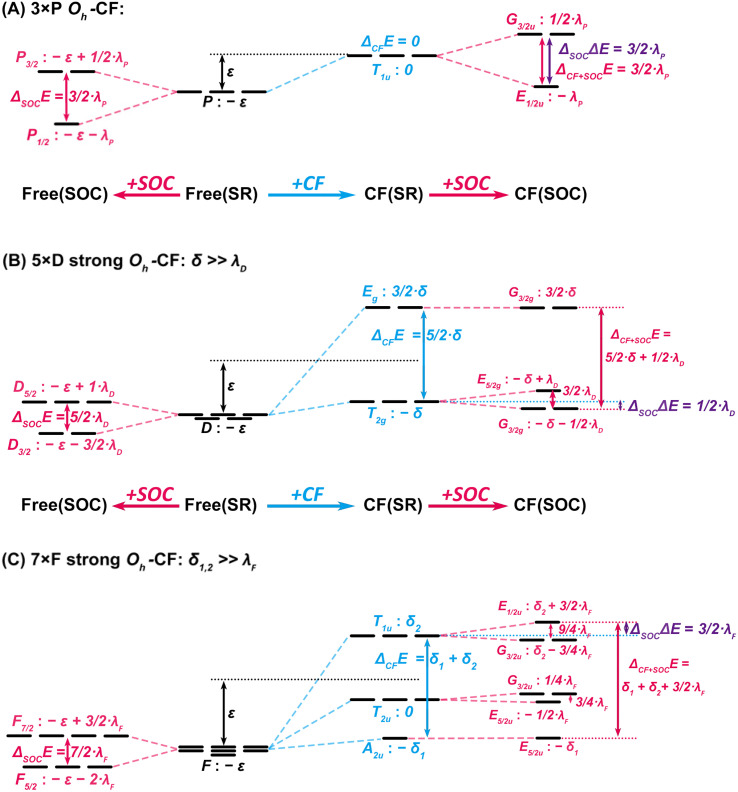
*O*_h_-Crystal-field and spin–orbit effects on p, d and f orbital levels. (Top) (A) p-Shell – independent shift *ε* by the CF and splitting 3/2·*λ*_P_ by SOC. (Middle) (B) d-Shell – shift *ε* and splitting 5/2·*δ* by the CF, partially quenching the 5/2·*λ*_D_ SO splitting. (Bottom) (C) f-Shell – shift *ε* and splitting *δ*_1_, *δ*_2_ by CF, significantly quenching the 7/2·*λ*_F_ SO splitting.

Fig. 4 displays the joint action of SOC and of a CF of *O*_h_ symmetry on atomic p, d and f levels. The larger the angular momentum, the smaller the SO splitting and the more easily it is reduced by crystal or ligand fields. For *P* or *T*_1u_ symmetry, the direct SOC in the valence shell is further enhanced by the Pauli-repulsion of an energetically adjacent noble-gas p^6^ core shell due to the radial p_1/2_–p_3/2_ splitting (Fig. 3A). The SO splitting is enlarged in the early period 7 by additional core/valence hybridization. It is then necessary to treat the whole atomic core at the SOC level, or apply small-core SOC effective-core-potentials (SOC-ECP).

### The U-6p-induced SO splitting of the O-2s,2p band in δ-[UO_3_]

**Fig. 5:**
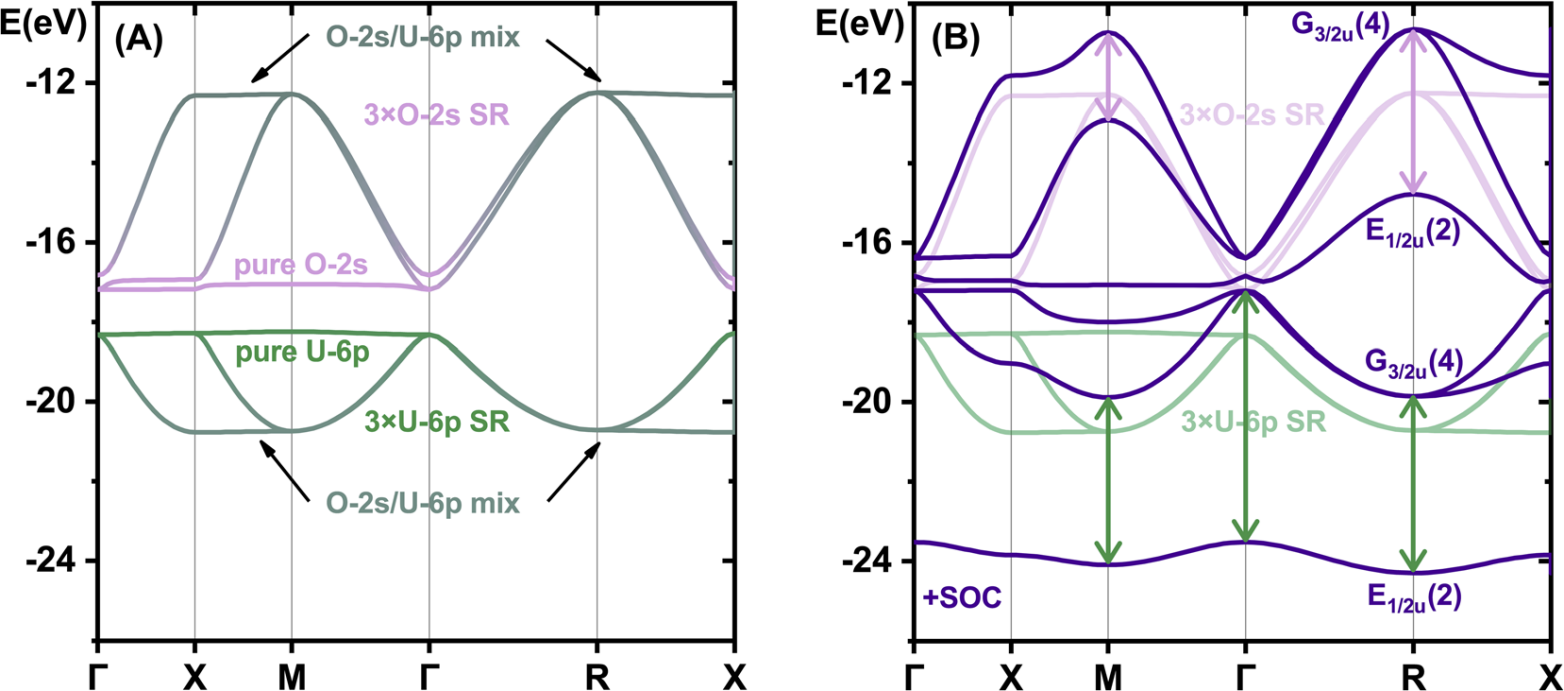
Band structure of δ-[UO_3_] in the U-6p/O-2s semi-core region. Energies in eV, lower Fermi edge set to zero. PBE calculations with VASP. (Left) (A) Projected SR bands, U-6p in green, O-2s in lilac. (Right) (B) SO split bands in purple, and transparent spin-averaged SR bands: dominantly U-6p in green and dominantly O-2s in lilac. Green and lilac double-arrows highlight the SO splitting.

The compensating bonding and anti-bonding overlap interactions of the U-6p^6^ semi-core with the 3O–2s^2^ inner-valence shells nicely show (Fig. 5A) the dependence on direction in space. Further details about how symmetry determines the U–O interactions at different points of the Brillouin zone are displayed in Fig. S7.† At the translationally symmetric Γ and -antisymmetric R points, the U-6p/O-2s manifolds remain *T*_1u_ triply degenerate (at the popular approximate SO-averaged SR level). SO coupling leads to a really significant energy splitting indicated in Fig. 5B by green and lilac double-arrows. The biggest splitting of 6.3 eV occurs for orbitals of rather pure U-6p character at the Γ point. At the M point, the lower U-6p dominated band is split by 4.2 eV, and the upper O-2s dominated band by 2.2 eV, reflecting an approximate 2 : 1 orbital mixing. At the optimally overlapping R point, the SO splitting of the lower and upper bands is respectively 4.4 eV and 4.1 eV, corresponding to nearly equal U-6p/O2s mixing. Obviously, the light-atomic O-2p valence band is incorrectly reproduced by the SR approximation without SO coupling.

**Fig. 6:**
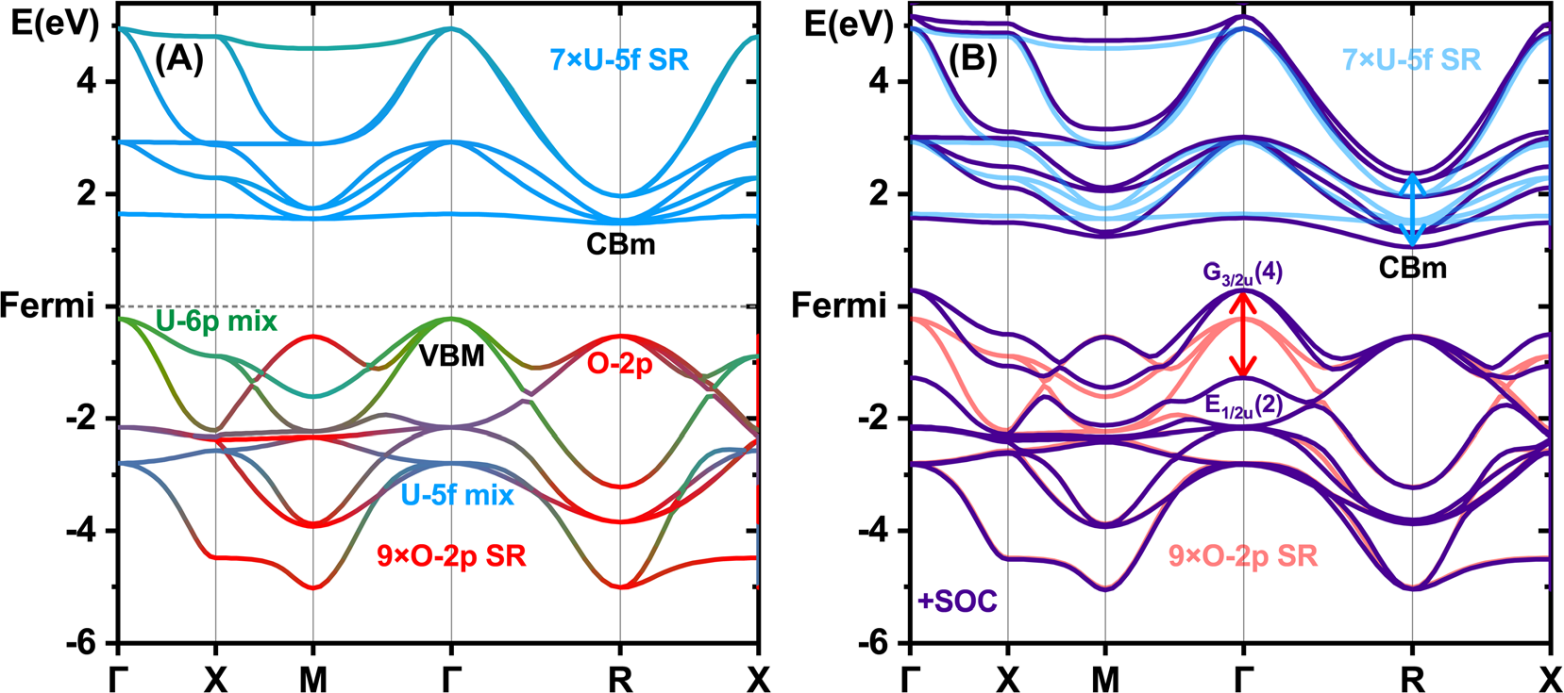
Band structure of δ-[UO_3_] in the O-2p/U-5f6d valence and conduction regions. Energies in eV, lower Fermi edge set to zero. PBE calculations with VASP. (Left) (A) Projected SR bands, O-2p in red, U-6p in green and U-5f in blue (both stronger × 8). (Right) (B) SO split bands in purple, and transparent spin-averaged SR bands: dominantly O-2p valence in red and dominantly U-5f virtual-conduction in blue. Red and light-blue double-arrows highlight the largest SO splitting.

The valence band of δ-[UO_3_] (Fig. 6) consists of O-2p orbitals dative-bonding into U-5f6d(7sp) and mixing with semi-core U-6p. Fig. 6B shows pronounced SO splitting, mainly for orbitals with the U-6p admixture. Bonding orbitals of O-2p/U-5f type (at Γ and X points) and of O-2p/U-6d type (at M and R, Fig. S9†) are hardly affected by SO coupling (quenched by CF). The same holds for α-[UO_3_] and [ThO_2_] (Fig. S13 and S25†).

The U-6p semi-core shell influences the valence interactions by two different mechanisms: 1st by Pauli-orthogonalization/repulsion without U-6p^6^ occupation change, 2nd by mixing U-6p and O-2p, reducing the U-6p character of the semi-core band a little. Both mechanics push the O-2p levels up toward the VBM, more by the outer and higher 6p_3/2_ than by the inner and lower 6p_1/2_, resulting in a large SO splitting of the O-2p valence band maximum of about 1½ eV. These effects are most pronounced for δ-[UO_3_] at the translationally symmetric Γ point with unbroken local *O*_h_ symmetry, where no damping of U-p SO coupling by the CF happens. The special *O*_h_ symmetry also keeps most of the SO splitting of the f-type conduction bands at the R point, where 7 nearly degenerate atom-like U-5f orbitals are not perturbed by interaction with O-2p, while the strong *O*_h_ CF with O-2p coordination significantly quenches the SOC of U-5f at the Γ point. AO percentages of the occupied valence and virtual conduction COs at the R and Γ points are displayed in Table S21.† At the X point, there are still two degenerate highest-energy orbitals pushed up by U-6p with considerable SO splitting. At the M point, the highest-energy orbital is of pure O-2p character without any SO splitting. At other points in the Brillouin zone there is little or no U-6p mixing and little or no pushing from below and SO splitting.

An approximate calculation with U-6p as a frozen core shell (using AMS-BAND) supplies important additional insight about how U-6p influences the VBM (see also Fig. S8†). At the VBM of δ-[UO_3_], *ca.* ¾ of the PFB-induced SO splitting comes from the Pauli-repulsion which is correctly reproduced by a simplistic 6p-frozen model, while more advanced effective core potentials ^69^ would also simulate the SO-dependent U-6p mixing effect. Anyway, a safer approach is the explicit inclusion of the U-6p shell into an extended valence shell.

A projected band analysis (Fig. S10–S27†) explains why the other [UO_3_] phases and [ThO_2_] exhibit significantly reduced SO splitting. The reduced symmetry at the U atom quenches the SO coupling partly. The U-6p PFB semi-core/valence mixing is weaker for 2 short plus 4–6 long U–O bonds than for 6 medium-short bonds; the U-6p admixture does not appear at the VBM but somewhere in the middle of the valence band and does not affect the “observable” size of the band gap. High symmetry around U and short U–O bonds will boost the PFB effect by the outer U-6p core shell and the SO coupling effects at the VBM. This is confirmed by the results of [UO_3_] solids under high pressure in ESI, Section S7.†

## Conclusions

We have identified three mechanisms contributing to the SO splitting of light-atomic valence bands in heavy atomic compounds:

(i) The usual dative bonding by electron-pairs from Lewis-basic ligands into the (*n* − 2)f, (*n* − 1)d and/or *n*p valence shells of the heavy transition metals transfers a fraction of the spin–orbit splitting (typically of the order of less than an eV), which is often reduced by the asymmetric crystal fields. In the typical valence energy range, this quenching is least pronounced for small angular momenta (*i.e.*, for p-orbitals of the p-block elements).

(ii) The heavy atomic spin–orbit splitting of energy and radius of the outer noble gas (*n* − 1)p^6^ core shell into p_1/2_^2^ and p_3/2_^4^ sub-shells is an order of magnitude larger. The ortho-normalization onto the occupied core shells required by the Pauli exclusion principle transfers this core splitting into the overlapping valence shells of the bonded ligands, raising the valence shells (pushing up from below) in a spin-dependent manner.^70,71^ This is the dominant contribution to the SO splitting in the present cases.

(iii) In addition, the comparatively weakly bound outer core orbitals of very heavy atoms can mix with ligand valence orbitals, allowing some core electrons to distribute into vacancies of the light-atomic valence shells. This stabilizes the core shell and partially destabilizes the valence shell, also pushing from below. In general, and also in the present case of [UO_3_], the relativistic behaviour of the valence electrons has two origins: orthogonality onto the strongly relativistic inner core orbitals (Pauli repulsion), and the relativistic terms of the Hamiltonian acting directly on the inner tails of the core-penetrating valence orbitals.^72^

Spin–orbit coupling needs careful consideration in solid-state science. We found an exciting example, the δ-phase of the technically important [UO_3_] solid, where the SO splitting in the O-2p valence band can actually be “seen” in terms of a band gap significantly reduced in comparison to reliable calculations at the scalar relativistic level with quenched spin–orbit coupling. δ-[UO_3_] shows a very pronounced “pushing from below”, *i.e.* the Pauli-repulsion and the valence activity of the U-6p semi-core shell, which is often counted as a chemically inactive noble-gas shell. High local symmetry at the heavy transition element and short interatomic distances such as in the δ-[UO_3_] phase, or at elevated pressures or in strained confinement, increase both the admixture of low-energy orbitals into the valence band and the magnitude of the valence SO splitting.

Molecular chemistry and ligand field theory of transition metal complexes are conceptually and computationally simpler than in the solid state. Therefore, the solid-state theory had to get by with simple, more easily manageable model approaches. The higher accuracy and reliability demands of chemistry led to some delay in advance, but then led to more advanced approaches. Both fields can now cross-fertilize each other.

The common non-relativistic model physics for materials science is a well-defined closed theory with a toolbox, forming a whole grid of ladders of approximations. A certain empirical experience is required to achieve chemical accuracy, but one will not always achieve it. In contrast, relativistic electron theory is a complex open theoretical framework. For chemical accuracy, relativity should be taken into account at least in periods 6 and 7, although it may cancel out in some applications. Computationally the lowest order of relativistic approximation is usually sufficient, where only two new terms show up in the Hamiltonian.^30^ The kinetic (‘velocity-mass’ and ‘zitter’) one-electron term can easily be included in the common non-relativistic framework of real (1-component) orbitals with a spin-label. The spin–orbit coupling term requires quaternionic (4-component) or bi-quaternionic orbitals. This leads to the spinor-orbital picture of ‘double-group’ symmetry; it often requires up to an order of magnitude more computational effort. But then one has achieved the relativistic goal, and the main problem still remaining is with nonrelativistic electron-interaction.

Consideration of the valence-activity of semi-core shells of the heavy elements, and the large SO coupling effects induced thereby, offers new aspects in solid-state, materials and chemical sciences of the heavy elements.

## Data availability

The data that support the findings of this study are presented in this paper and in the ESI file.†

## Author contributions

Han-Shi Hu and Jun Li: conceptualization, resources, editing; Shi-Ru Wei: main investigation, data curation, computations, formal analysis; W. H. Eugen Schwarz and Shi-Ru Wei: writing, editing.

## Conflicts of interest

There are no conflicts to declare.

## Supplementary Material

SC-016-D4SC08151J-s001
