# Supplementary material for: Valence activity of SO-coupled atomic core shells in solid compounds of heavy elements
Source: Chem Sci. 2025 Feb 21;16(16):6744–54. doi: 10.1039/d4sc08151j (PMC11934502; doi:10.1039/d4sc08151j)
Supplement: SC-016-D4SC08151J-s001 [file SC-016-D4SC08151J-s001.pdf]

Supplementary Information (SI) for Chemical Science.
This journal is © The Royal Society of Chemistry 2025

Supporting Information

Valence Activity of SO-Coupled Atomic Core Shells in Solid Compounds of Heavy Elements

Shi-Ru Wei[a], Han-Shi Hu[a], W. H. Eugen Schwarz[a,b,]* and Jun Li[a,c,d]*

[a] Theoretical Chemistry Center, Department of Chemistry; and Engineering Research Center of Advanced Rare-Earth Materials of the Ministry of Education; Tsinghua University, Beijing 100084, China.
[b] Physical and Theoretical Chemistry Lab, Department of Chemistry and Biology, Faculty of Science and Technology, University of Siegen, Siegen 57068, Germany.
[c] Department of Chemistry, Southern University of Science and Technology, Shenzhen 518055, China.
[d] Fundamental Science Center of Rare Earths, Ganjiang Innovation Academy, Chinese Academy of Sciences, Ganzhou 341000, China.

*Corresponding authors: W. H. Eugen Schwarz, eugen.schwarz@uni-siegen.de
and Jun Li, junli@tsinghua.edu.cn

This PDF file includes:

Supplementary Text
Figs. S1 to S39
Tables S1 to S21
Supplementary References (1 to 97)

Contents

Section 1. Quantum Chemical Methods for $5f^06d^0$ Oxides [UO_3] & [ThO_2] ... 3
Different methods ... 3
Solid [UO_3] Phases ... 3
Section 2. Computational Methodology ... 4
Table S1 ... 5
Section 3. Geometric Optimization ... 5
Table S2 ... 6
Table S3 ... 7
Fig. S1. ... 7
Table S4 ... 8
Section 4. Effect of SO Coupling on Band Gap and Band Structure ... 8
Table S5 ... 9
Table S6 ... 10
Table S7 ... 10
Table S8 ... 11
Table S9 ... 11
Table S10 ... 12
Table S11 ... 12
Fig. S2. ... 12
Section 5. U-6p "pushing from below" and SO splitting in δ-[UO_3] ... 13
Fig. S3. RDF for U^{6+} and O^0 ... 13
Fig. S4. ... 14
Fig. S5. ... 14
Table S12 ... 15
Fig. S6. ... 15
Fig. S7. ... 16
Fig. S8. ... 17
Fig. S9. ... 17
Section 5a. *U-6p Valence Admixture in Various Phases and under Pressure* ... 18
Figs. S10-S33 ... 18
Section 6. From Molecular $U(OH)_6$ to Solid δ-[UO_3]: SO Coupling vs. Crystal Field Splitting ... 32
Fig. S34a ... 32
Fig. S34b. ... 34
Figs. S35a-S35d ... 35
Fig. S36. ... 38
Table S13 ... 39
Table S14 ... 39
Fig. S37. ... 40
Table S15 ... 41
Table S16 ... 42
Fig. S38. ... 43
Table S17 ... 44
Table S18 ... 45
Section 7. SOC in High-Pressure Phases ... 45
Table S19 ... 46
Table S20 ... 47
Table S21 ... 48
Fig. S39. ... 49
SI References ... 50-53

Section 1. Quantum Chemical Methods for $5f^0 6d^0$ Oxides [UO_3] & [ThO_2]

Different methods

It is well-known that the pure Local Density Approximation (LDA) and Generalized Gradient Approximation (GGA) tend to underestimate the band gaps of solids (*1-4*). A typical example for the failure is the Mott insulator [UO_2]: popular middle-quality GGA density functionals miscalculate the electronic structure of [UO_2] even qualitatively, predicting metallic conductivity instead of the experimental gap of about 2.1 eV (*5-7*). The introduction of the semi-empirical DFT+U method(*5, 8, 9*) with two adjustable parameters U and J can often reduce the band-gap problems. One can then reproduce the band gaps and also the geometric crystal parameters by fitting the additional "+U" parameters to the specific system. U = 4.5 eV and J = 0.54 eV (*10*), have often been used for [UO_2] and then also for other [UO_x] phases (for x from 2 to 3) (*11-24*). The advantage of the DFT+U approach is that it does not change the computational expenses. It has been widely used in actinide solids and surfaces research (*5, 25-44*). However, an occupation matrix control (OMC) should be included to guarantee for a correct ground state was reached (*21-24, 44-46*). Yet the problem remains that sometimes no choice of the U-J parameters yields approximately correct band gaps, such as for [ThO_2] (*31*) or α-[UO_3] (*12*).

Both [ThO_2] and the various [UO_3] phases have similar electronic structure with an O-$2p^6$ dominated valence band and an An-$5f^0$ type conduction band. Even simple GGA functionals can correctly predict a positive band gap, but qualitatively too smaller in magnitude by about an eV. Empirical DFT+U improves these results in some cases, but can't solve the problem for [ThO_2] or α-[UO_3]. In contrast to the GGA, the Hartree-Fock (HF) method always overestimates the band gaps (*3*). A hybrid mixture can give better results (*47*). Using the HSE range-separated hybrid functional, He et al. (*17*) found a too big band gap for α-[UO_3], while Wen et al. (*48*) found a reasonable band gap for [ThO_2], well agreeing with the experiment. More advanced but more costly methods such as density matrix embedding theory, dynamical mean-field theory, and the Green's function (GW) approach etc. can yield more reliable results (*3, 49*).

Solid [UO_3] Phases

[UO_3] is an economically important uranium oxide in the highest oxidation state, which appears in the nuclear fuel cycle, also in the spent fuel. Seven polymorphs of [UO_3] have been identified experimentally, including α-, β-, γ-, δ-, ε-, ζ- and η-[UO_3] (*50, 51*). Further, slightly different structures have been communicated for ε-, and ζ-[UO_3]. Pickard et al. were the first to perform density functional calculations (LDA and GGA), theoretically reproducing the geometric structures of α-, δ-, and η-[UO_3] (*52*). Then He et al. calculated electronic and geometric structures for α-, δ-, and γ-[UO_3] at the LDA+U and HSE levels (*17*). Geng et al. calculated the δ-[UO_3] phase at the GGA level (*18*). Brincat et al. systematically verified the stationarity of the α-, β-, γ-, δ-, and η-[UO_3] phases at the GGA+U level, and compared the relative stabilities of different phases under geochemical pressures (*13*). High pressure-induced phase transition of [UO_3] were studied in more detail by Ma et al., with GGA+U, they predicted three new structures for [UO_3] (*12*). Shields et al. examined the effects of pressure on the structure and vibrational properties for α-, β-, γ-, and δ-[UO_3] by DFT+U approaches (*11*). All these works neglected spin-orbit coupling.

Our present computational research focuses on the electronic-structure of solid [UO_3] phases, including the SO coupling and the U-6p semi-core orbital mixing effects. Different density functional approximations are compared to obtain a more reliable picture for the electronic structures of the different [UO_3] polymorphs. The various calculated literature results need a re-evaluation in view of the considerable SO coupling effects. We here also explore the Pauli-repulsion and core-valence mixing effects of the SO split U-6$p_{1/2}$, 6$p_{3/2}$ semi-core shells, known in molecular chemistry as "pushing from below" PFB.

In the next Section 2 we describe the applied computational methodology. Then our results are discussed with respect to five points: Section 3, the geometric structures of the [UO_3] phases; Section 4, the SO coupling effect on band structure and valence-conduction band gaps; Section 5, the PFB phenomenon, particularly impressive for the SO coupled high-symmetry δ-[UO_3] phase; Section 6 the relation of the solid phase to molecular single-center complexes and CF quenches the SO coupling; and Section 7 the impact of SO coupling on the high-pressure phases. In a concluding section, we summarize some general chemical insights concerning the often neglected spin-orbit coupling, and the decreasing core-valence gap for the heaviest elements. We highlight the evidences of these effects in all phases of [UO_3], and stress that more attention must be paid to the spin-orbit coupling in heavy element chemistry.

Section 2. Computational Methodology

For the solid-state investigations of [UO_3], the Vienna Ab initio Simulation Package (VASP) (*53-56*) was used in Geometry Optimization, Band Structures and Density of States (DOS) calculations. The AMS-BAND package (*57-61*) was also used, in particular for further wave-function analyses. For comparison, molecular $U(OH)_6$ was studied using the AMS-ADF package (*62-64*).

The investigations were carried out by applying Kohn-Sham density functional approximations, at first at the scalar-relativistic (SR) level, then with inclusion of SO coupling. The wavefunction was represented by a Kramers and geometric symmetry restricted single closed-shell configuration. We have used the density-gradient Perdew–Burke–Ernzerhof (PBE) (*65, 66*), the PBE+U approach (*5, 8, 9*) (with U = 4.5 eV, J = 0.54 eV (*10*) and U_{eff} = 3.96eV), the exchange-hybrid approach of Heyd-Scuseria-Ernzerhof (HSE06) (*67*) functional and a quasi-particle energies improvement by a single-shot G_0W_0 Green function approximation (*68-72*) based on the PBE. For comparison, both scalar relativistic (SR) and SO coupled calculations were carried out. The Zero Order Regular Approximation (ZORA) (*73-75*) was used in the AMS codes, and also in the VASP code based on the projector augmented wave (PAW) method (*76-78*).

With VASP, the cut-off energy of the plane-wave basis sets was set to 500 eV, with a Γ-centered Brillouin zone sampling (see Table S1). Different k-meshes were used in order to control the computation time. For highly symmetrical and smaller unit cells as for [ThO_2] and δ-[UO_3], we always used the better k-meshes. For the other phases, we also used less k points, namely for the geometric structure optimization which does not require the full number of k points. For the single point DOS calculation with PBE or PBE+U, we use a satisfactory bigger k-mesh. However, for HSE calculation, we have to use a smaller k-mesh because of computing speed limitations. Some simple tests did show that the quality of the k-mesh had little effect on the band gaps.

Table S1. k-meshes for all phases.

Phase	Space group	Geometric Optimization (PBE)	DOS (PBE)	DOS (PBE+U)	DOS (HSE)	DOS (G_0W_0)
$[ThO_2]$	$Fm\bar{3}m$	15×15×15	15×15×15	15×15×15	15×15×15	15×15×15
α-$[UO_3]$	$P\bar{3}m1$	9×9×6	9×9×6	9×9×6	9×9×6	9×9×6
	C2mm	10×10×8				
	C2	9×5×8				
β-$[UO_3]$	$P2_1$	7×3×3	9×3×5	9×3×5	5×2×2	
γ-$[UO_3]$	Fddd	5×5×5	5×5×5	5×5×5	2×2×3	
	$I4_1$	5×5×7	5×5×7	5×5×7	2×2×2	
η-$[UO_3]$	$P2_12_12_1$	7×7×5	9×9×7	9×9×7	4×4×3	
δ-$[UO_3]$	$Pm\bar{3}m$	9×9×9	11×11×11	11×11×11	11×11×11	11×11×11
x-$[UO_3]$	P63mmc	9×9×4	11×11×5	11×11×5		
y-$[UO_3]$	$Fm\bar{3}m$	9×9×9	13×13×13	13×13×13	9×9×9	
z-$[UO_3]$	Pm3n	7×7×7	11×11×11	11×11×11	9×9×9	

With AMS-BAND, localized NAO and STO basis sets of TZ2P quality were used with a frozen mall-core. For the molecular calculations, all-electron STO basis sets of TZ2P quality (*79, 80*) were used. The convergence criterion of the electronic SCF steps was set to 1×10^{-6} eV. The solid geometry optimizations were done by a conjugate-gradient algorithm at the scalar-relativistic SR-PBE level. The ionic steps were converged for Hellmann-Feynman forces less than 0.01 eV/Å.

VASPKIT (*81*) was used for Generating input files and post-processing in VASP calculations. Band structures from HSE and G_0W_0 calculations were produced by Wannier function interpolation using Wannier90 (*82*). Mulliken population (*83*), Inverse Crystal Orbital Bond Index (ICOBI) (*84*) and Crystal Orbital Hamilton Populations (COHP) (*85, 86*) analyses were carried out with the Aathen Lobster-5.0.0 program (*87, 88*). The VESTA (*89*) and AMS-GUI was used to produce iso-surfaces for crystalline orbitals and molecular orbitals.

<u>Section 3</u>. Geometric Optimization

Although PBE+U can describe the electronic structure better than PBE because PBE+U improves the predicted band gap, becoming closer to the experimental data, we find that the optimized geometric structure from PBE better agrees with experimental data than PBE+U (Table S2). Anyway, there is no essential difference. We compare three possible α-$[UO_3]$ phases from experiment and DFT calculation in Table S3, in our calculation, the structures of $P\bar{3}m1$ α-$[UO_3]$ and C2 α-$[UO_3]$ are almost the same. Structure data for six phases are summarized in Tabel S4. Structures for six normal phases and two high-pressure stable phases $P6_3/mmc$ x-$[UO_3]$ and $Fm\bar{3}m$ y-$[UO_3]$ $[UO_3]$ phases (*12*) are shown in Fig. S1.

Table S2. Lattice parameters for all [UO_3] phases and [ThO_2], using the conventional cell, comparing PBE (this work), PBE+U (Brincat (*13*) and Wen (*48*)) and experimental results (*13*).

Phase	Space group	Formula units per cell	Method	Lattice parameters (Å)		
				a (Δ%) [a]	b (Δ%) [a]	c (Δ%) [a]
δ-[UO_3]	$Pm\bar{3}m$	1	Exptl.	4.17	4.17	4.17
			PBE	4.16 (−0.2)	4.16 (−0.2)	4.16 (−0.2)
			PBE+U [b]	4.20 (0.7)	4.20 (0.7)	4.20 (0.7)
α-[UO_3]	$P\bar{3}m1$	1	Exptl.	3.97	3.97	4.17
			PBE	3.81(−4.0)	3.81(−4.0)	4.15 (0.5)
			PBE+U [b]	3.85 (−3.0)	3.85 (−3.0)	4.18 (0.2)
β-[UO_3]	$P2_1$	10	Exptl.	10.34	14.33	3.91
			PBE	10.78 (4.3)	14.32 (0.0)	4.10 (4.9)
			PBE+U [b]	10.81 (4.6)	14.33 (0.0)	4.19 (7.2)
γ-[UO_3]	Fddd	32	Exptl.	9.79	19.93	9.71
			PBE	9.86 (0.7)	20.17 (1.2)	9.85 (1.4)
			PBE+U [b]	9.94 (1.5)	20.68 (3.8)	9.93 (2.3)
γ-[UO_3]	$I4_1$	16	Exptl.	6.90	6.90	19.98
			PBE	6.97 (1.0)	6.97 (1.0)	20.17 (1.0)
			PBE+U [b]	7.02 (1.7)	7.02 (1.7)	20.68 (3.5)
η-[UO_3]	$P2_12_12_1$	4	Exptl.	7.51	5.47	5.22
			PBE	7.57	5.54	5.24
			PBE+U [b]	7.76 (3.3)	5.56 (1.6)	5.34 (2.3)
[ThO_2]	$Fm\bar{3}m$	4	Exptl.	5.60	5.60	5.60
			PBE	5.62 (0.4)	5.62 (0.4)	5.62 (0.4)
			PBE+U [c]	5.67 (1.3)	5.67 (1.3)	5.67 (1.3)

[a] The percentage deviation of computed structural data from the experimentally derived ones.
[b] Brincat's work using PBE+U with U_{eff} = 4.0 eV
[c] Wen's work using PBE+U with U_{eff} = 4.0 eV

Table S3. Comparison of 3 possible structures of α-[UO_3], from PBE optimizations with VASP.

Phase	α-[UO_3]		
Space Group	C2mm [a]	P$\bar{3}$m1	C2
# of formula units per cell	1	1	1
# of U-O bonds	8	8	8
U-O distances (in Å)	2×2.074	2×2.075	2×2.075
	2×2.105	6×2.247	2×2.217
	4×2.365		4×2.262
Average	2.23±0.2	2.21±0.1	2.20±0.1
O-U-O Angle (degree)	90	78.0	77.6~78.6
Energy per unit (eV)	−37.72	−38.04	−38.04

[a] with an imaginary frequency

Fig. S1. Structures of 8 phases, optimized by PBE. (A) α-[UO_3], (B) β-[UO_3], (C) I41-γ-[UO_3], (D) η-[UO_3], (E) δ-[UO_3], (F) $P6_3/mmc$ x-[UO_3] at 80GPa, (G) Fm$\bar{3}$m y-[UO_3] at 80GPa, (H) [ThO_2].

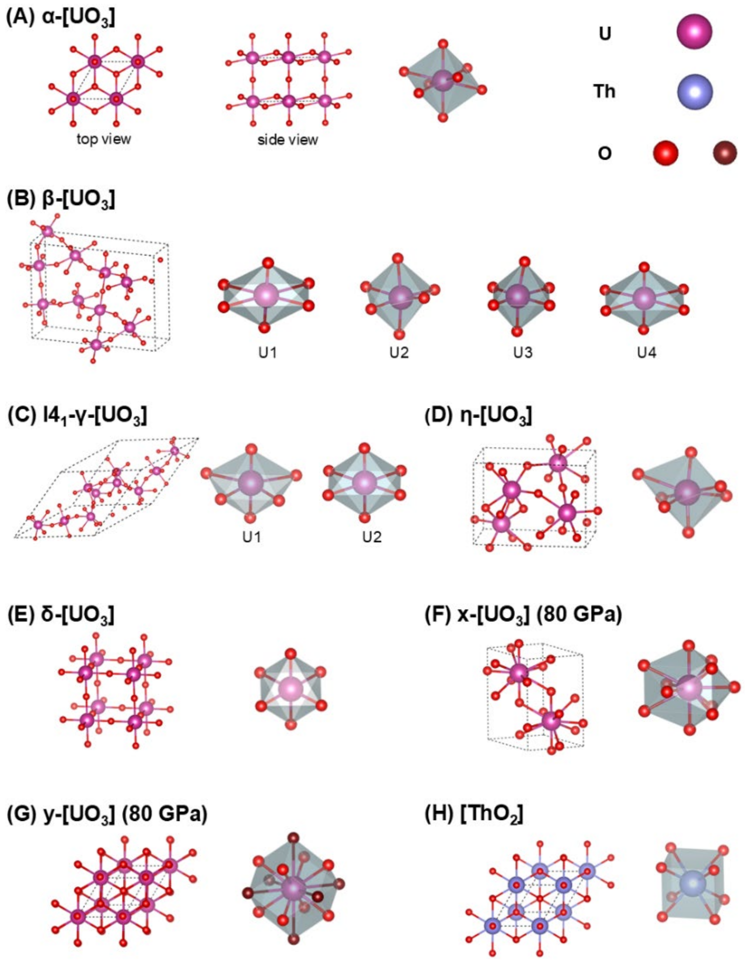

Table S4. Structure data for one [ThO_2] and five [UO_3] phases: Phase name, space group, atoms per unit cell, number of atoms per atom type, coordination number CN, metal−O bond distances in Å (the short uranylic ones < 2 Å are highlighted in blue). From quantum-chemical scalar-relativistic Kohn-Sham-PBE optimizations with VASP.

Phase	δ-[UO_3]	α-[UO_3]	β-[UO_3]				γ-[UO_3]		η-[UO_3]	[ThO_2]
Space group	Pm$\bar{3}$m	P$\bar{3}$m1	P2_1				Fddd/I4_1		P$2_12_12_1$	Fm$\bar{3}$m
units/cell	1	1	10				8		4	1
atom type	U1	U1	U1	U2	U3	U4	U1	U2	U1	Th1
# type/cell	1	1	2	4	2	2	4	4	1	1
# M-O	6	8	6	6	6	6	6	6	7	8
Strut. Motif [a]	U(=O=)$_6$	(=O=U=O=)<br>·6(←O-)	(≡O≡U≡O≡)·4(←O-)							Th(-O-)$_8$
U/Th-O distances (in Å)			2×1.84	1.96<br>2.03	1.80<br>1.81	1.79<br>1.80	2×1.82	2×1.91	1.88<br>1.90	
	6×2.08	2×2.08		2.11<br>2×2.12<br>2.15						
		6×2.25	2×2.32		2×2.28		2×2.34<br>2×2.35	4×2.22	2.20<br>2.24<br>2.32	
			2×2.47		2×2.48	2×2.41<br>2×2.44			2.44<br>2.47	8×2.43
Average	2.08	2.21	2.21	2.08	2.18	2.22	2.21	2.12	221	2.43
			2.15				2.17			

[a] Here, U≡O indicates a short 'triple' bond ≈ 1.8 to 1.9 Å; U=O indicates a 'double' bond ≈ 2.1 Å; Th-O indicates a 'single' bond 2.3 Å or longer.

Section 4. Effect of SO Coupling on Band Gap and Band Structure

Here, we discuss the energies of the valence band Maximum $E(VBM)$, of the conduction band minimum $E(CBm)$, the respective energy gap ΔE_{gap}, and their changes Δ_{SOC} from the SR approximation to the more realistic SO coupled level. We define:

$$\Delta E_{gap}(SR) = E_{SR}^{CBm} - E_{SR}^{VBM} > 0$$

$$\Delta E_{gap}(SOC) = E_{SOC}^{CBm} - E_{SOC}^{VBM} > 0$$

$$\Delta_{SOC} E(VBM) = E_{SOC}^{VBM} - E_{SR}^{VBM} > 0$$

$$\Delta_{SOC} E(CBm) = E_{SOC}^{CBm} - E_{SR}^{CBm} < 0$$

$$\Delta_{SOC}\Delta E_{gap} = \Delta E_{gap}(SOC) - \Delta E_{gap}(SR) = \Delta_{SOC} E(CBm) - \Delta_{SOC} E(VBM) < 0$$

Table S5. Calculated band gaps (in eV) of several [An-5f^0 $O_{2,3}$] phases, at various DFT levels (PBE, PBE+U, HSE, G_0W_0@PBE), scalar-relativistic approximation and with spin-orbit coupling (+SOC), using VASP, at the bottom compared with the experimentally derived value (Exptl.). Blue calculated values are too small by more than −½ eV, red values are too large by +½ eV (Δ in parentheses is the calculation error). $\Delta_{SOC}\Delta E_{gap}$ is the reduction of the band gap due to SOC.

phase	δ-[UO_3]	α-[UO_3]	β-[UO_3]	γ-[UO_3]	γ-[UO_3]	η-[UO_3]	[ThO_2]
Space group	$Pm\bar{3}m$	$P\bar{3}m1$	$P2_1$	Fddd	$I4_1$	$P2_12_12_1$	$Fm\bar{3}m$
PBE	1.67 (Δ=−0.5)	1.68 (Δ=−1.0)	1.44 (Δ=−0.7)	1.89 (Δ=−0.5)	1.89 (Δ=−0.5)	1.91	4.45 (Δ=−1.4)
PBE+SOC	0.75 (Δ=−1.4)	1.31 (Δ=−1.4)	0.98 (Δ=−1.2)	1.46 (Δ=−0.9)	1.46 (Δ=−0.9)	1.38	4.35 (Δ=−1.5)
PBE+U	2.25 (Δ=+0.1)	1.97 (Δ=−0.7)	2.34 (Δ=+0.2)	2.79 (Δ=+0.4)	2.79 (Δ=+0.4)	2.70	4.83 (Δ=−1.1)
PBE+U+SOC	1.26 (Δ=−0.9)	1.49 (Δ=−1.1)	1.77 (Δ=−0.4)	2.37 (Δ=−0.0)	2.37 (Δ=−0.0)	2.13	4.74 (Δ=−1.2)
HSE	3.26 (Δ=+1.1)	2.96 (Δ=+0.3)	3.14 (Δ=+1.0)	3.68 (Δ=+1.3)	3.69 (Δ=+1.3)	3.60	6.15 (Δ=+0.3)
HSE+SOC	2.28 (Δ=+0.1)	2.42 (Δ=-0.2)	2.61 (Δ=+0.4)	3.23 (Δ=+0.8)	3.23 (Δ=+0.9)	3.05	6.03 (Δ=+0.1)
G_0W_0@PBE	3.36 (Δ=+1.2)	3.26 (Δ=+0.6)					6.12 (Δ=+0.2)
G_0W_0@PBE+SOC	2.24 (Δ=+0.1)	2.67 (Δ=+0.0)					6.01 (Δ=+0.1)
Exptl.	*2.17*	*2.63*	*2.17*	*2.38*	*2.38*		*5.75~6.00*
$\Delta_{SOC}\Delta E_{gap}$	≈−1.0	≈−0.5	≈−0.5	≈−0.4	≈−0.4	≈−0.5	≈−0.1

As shown in Table S5, different methods significantly affect the band gaps, but all show similar $\Delta_{SOC}\Delta E_{gap}$ for a given phase. The different software AMS-BAND and VASP also show similar $\Delta_{SOC}\Delta E_{gap}$ (Table S6). We display $\Delta_{SOC}E(CBm)$, $\Delta_{SOC}E(VBM)$, the total reduction of the band gap by SO coupling, $\Delta_{SOC}\Delta E_{gap}$, and the AO populations at the VBM, from the DFT-PBE calculations of the α-, β-, γ-, η-, δ-[UO_3] and [ThO_2] phases in Table S7. A part of the SO induced band gap reduction $\Delta_{SOC}\Delta E_{gap}$ of the current [$AnO_{2,3}$] phases is due to the SO splitting of the An-5f dominated conduction band minimum CBm (0.25 to 0.3 eV for [UO_3], 0.42 for δ-[UO_3], <0.1 eV for [ThO_2]). That is understandable in terms of the overall atomic SO splitting of about 0.8 for U-5f (and also for U-6d, see Tables S13-S14), which is reduced by the lower symmetry of the atoms' environment in the crystal. Concerning the O-2p dominated valence band maximum VBM, its rise due to SO splitting is typically about 0 to 0.2 eV. This may be understood as due to the donation of the O-2p pairs into the formally empty U-5f6d valence shell. However, the SO induced rise of the VBM of 0.5 eV for δ-[UO_3] comes as a surprise. Below we trace it back to the usually missed antibonding U-6p semi-core admixture to the upper O-2p valence states. We note that the U-6p semi-core SO splitting is an order of magnitude larger than the U-5f and 6d SO splitting (see Table Tables S13-S14).

Table S6. Comparison of VASP and BAND software. Band gaps ΔE_{gap}, and reductions by spin-orbit coupling SOC $\Delta_{SOC}\Delta E_{gap}$ in eV.

software	phase		α-[UO$_3$]	β-[UO$_3$]	Fddd γ-[UO$_3$]	I41 γ-[UO$_3$]	η-[UO$_3$]	δ-[UO$_3$]	Fm$\bar{3}$m [ThO$_2$]
	Space group		P$\bar{3}$m1	P2$_1$	Fddd	I4$_1$	P2$_1$2$_1$2$_1$	Pm$\bar{3}$m	Fm$\bar{3}$m
VASP	ΔE_{gap}	PBE	1.68	1.44	1.89	1.89	1.91	1.67	4.45
		PBE+SOC	1.31	0.98	1.46	1.46	1.38	0.75	4.35
	$\Delta_{SOC}\Delta E_{gap}$		−0.4	−0.5	−0.4	−0.4	−0.5	−0.9	−0.1
AMS-BAND	ΔE_{gap}	PBE	1.59	1.36	1.81	1.81	1.84	1.58	4.41
		PBE+SOC	1.26	0.99	1.46	1.51	1.37	0.71	4.41
	$\Delta_{SOC}\Delta E_{gap}$		−0.3	−0.4	−0.3	−0.3	−0.5	−0.9	0.0

Table S7. Breakdown of energy changes (in eV) due to spin-orbit coupling (SOC) for the six different An(5f6d)^0-oxide phases: $\Delta_{SOC}E(CBm)$ and $\Delta_{SOC}E(VBM)$, and the total reduction of the band gap by SOC, $\Delta_{SOC}\Delta E_{gap}$, from SOC calculations. The An-np core+valence, the An-5f and the An-6d valence AO populations at the VBM, from scalar-relativistic calculations. Kohn-Sham DFT-PBE approximation, VASP code.

Oxide Phase	δ-[UO$_3$]	α-[UO$_3$]	β-[UO$_3$]	γ-[UO$_3$]	η-[UO$_3$]	[ThO$_2$]
Space group	Pm$\bar{3}$m	P$\bar{3}$m1	P2$_1$	Fddd/I4$_1$	P2$_1$2$_1$2$_1$	Fm$\bar{3}$m
$\Delta_{SOC}E(CBm)$ / eV	−0.42	−0.30	−0.26	−0.24	−0.31	−0.05
$\Delta_{SOC}E(VBM)$ / eV	+0.50	+0.07	+0.20	+0.19	+0.22	+0.05
An-np at VBM	12%	3%	7%	6%	5%	5%
An-5f at VBM	12%	1%	13%	14%	10%	6%
An-6d at VBM	0%	1%	1%	1%	1%	1%
$\Delta_{SOC}\Delta E_{gap}$ / eV	−0.92	−0.37	−0.46	−0.43	−0.53	−0.10

The AO populations at the VBM are very similar from PBE(SR) and HSE(SR) calculations (Table S8). In order to distinguish U-6p semi-core anti-bonding and U-7p valence-Rydberg bonding admixtures, a LOBSTER band analysis was performed on the VASP crystal wave-function (Table S10): there is a 5.4% U-6p admixture at the VBM of δ-[UO$_3$] what is expected to contribute up to ¼ eV spin-orbit energy raise. We can even directly see the anti-bonding character of O(2p)-U(6p) at VBM (Fig. S2).

We have carefully tested the different basis functions sets used in LOBSTER. The LOBSTER prescription appears to work better. We find that both the inner semi-core U-6p and the outer semi-Rydberg 7s,7p basis functions are important for the valence shell. Even the U-6s^2-core orbital has lost ca. ¼ e due to the Pauli-restricted overlap interactions with the six neighbor formal O^{2-}-2p^6 ligands, and the U-6p^6-semi-core shell has lost ca. ½ e. The poly-centric nature of O-U-O bonding appears also to mix in diffuse U-7sp hybrids (Table S9). The AO population results for the VBM of δ-[UO$_3$] from LOBSTER and VASP are consistent (Table S10). We also performed an ICOBI analysis for the U-O bonding in δ-[UO$_3$] (Table S11).

Table S8. δ-[UO_3] at the VBM, Γ point: Mulliken populations of atomic U-s,p,d,f orbitals from SR-VASP calculations with PBE vs. HSE density functionals.

CO Crystal Orbitals ↓	AO →	U-s		U-p		U-d		U-f	
Density Functional →	CO type↓	PBE	HSE	PBE	HSE	PBE	HSE	PBE	HSE
U-5f T_{1u}	virtual			0.05	0.04			0.61	0.64
U-5f T_{2u}								0.78	0.81
U-5f A_{2u}								0.97	0.97
O-2p/U-6p T_{1u}	valence			0.12	0.12			0.12	0.12
O-2p/U-5f T_{2u}								0.24	0.21
O-2p/U-5f T_{1u}				0.01	0.01			0.36	0.36
O-2s/U-7s A_{1g}	semi-core	0.06	0.06						
O-2s/U-6d E_g						0.13	0.13		
U-6p T_{1u} [a]				0.88	0.88				
U-6s A_{1g}	core	0.98	0.98						

[a] The 'degeneracy-driven bonding and anti-bonding' orbitals of U-6p/O-2s type appear at the R point. At the Γ point, there are three degenerate U-6p orbitals.

Table S9. Atomic orbital Mulliken populations of δ-[UO_3] from Kohn-Sham calculations (VASP and AMS-BAND), using the AMS-BAND and LOBSTER analyses. Different basis functions were compared and we found 7s and 7p basis function can improve the results in Lobster.

Atom	Shell	BAND code	LOBSTER (7s,7p)	LOBSTER (7s,--)	LOBSTER (--,7p)	LOBSTER (--,--)
U	6S	*2.09*	1.74	1.94	1.98	1.99
	6P	5.99	5.42	5.94	5.49	5.94
	5F	2.77	2.22	2.22	2.22	2.22
	6D	1.66	1.80	1.77	1.80	1.76
	7S	*−0.41*	0.56	0.26	-/-	-/-
	7P	0.02	0.63	-/-	0.66	-/-
	eff.charge	+1.88	+1.63	+1.87	+1.85	+2.09
O	O-2s	1.87	1.82	1.83	1.84	1.85
	O-2p	4.72	4.72	4.79	4.78	4.85
	O-3d	0.03	-/-	-/-	-/-	-/-
	eff.charge	−0.62	−0.55	−0.62	−0.62	−0.70
	Charge spilling [a]	-	1.42%	1.55%	1.48%	1.70%

[a] Charge Spilling is a measure for the quality of the reproduction of the crystal wave-function by the restricted atom-centered basis in the LOBSTER software. It should be smaller than 5%, in the range of 1%.

Table S10. δ-[UO_3]: The U-7s,6p,7p,6d,5f AO populations of the crystal orbital at the VBM at the Γ point, from VASP-LOBSTER calculations. Gerade U s,d orbitals do not contribute at the Γ point to the O-2p band.

AO	VASP	LOBSTER
U-7s	0	0
U-6d	0	0
U-5f	12%	11%
U-7p	12%	6.2%
U-6p		5.4%

Table S11. ICOBI for δ-[UO_3], under 0, 40, and 80 GPa, from VASP-LOBSTER calculations.

	ICOBI		
Pressure / GPa	80	40	0
U-O length/ pm	189.2	196.3	208.1
6s-2s	0.000	0.000	0.000
6s-2p	0.013	0.009	0.006
7s-2s	0.062	0.059	0.053
7s-2p	0.024	0.028	0.034
6p-2s	0.001	0.001	0.000
6p-2p	0.036	0.027	0.017
7p-2s	0.057	0.065	0.074
7p-2p	0.047	0.049	0.052
6d-2s	0.164	0.153	0.133
6d-2p	0.398	0.398	0.392
5f-2s	0.058	0.047	0.033
5f-2p	0.432	0.442	0.460
total	1.292	1.278	1.254

Fig. S2. δ-[UO_3] iso-surfaces of the 3-degenerate T_{1u} type crystal orbitals at VBM, with iso-value = 0.02 e/Å^3.

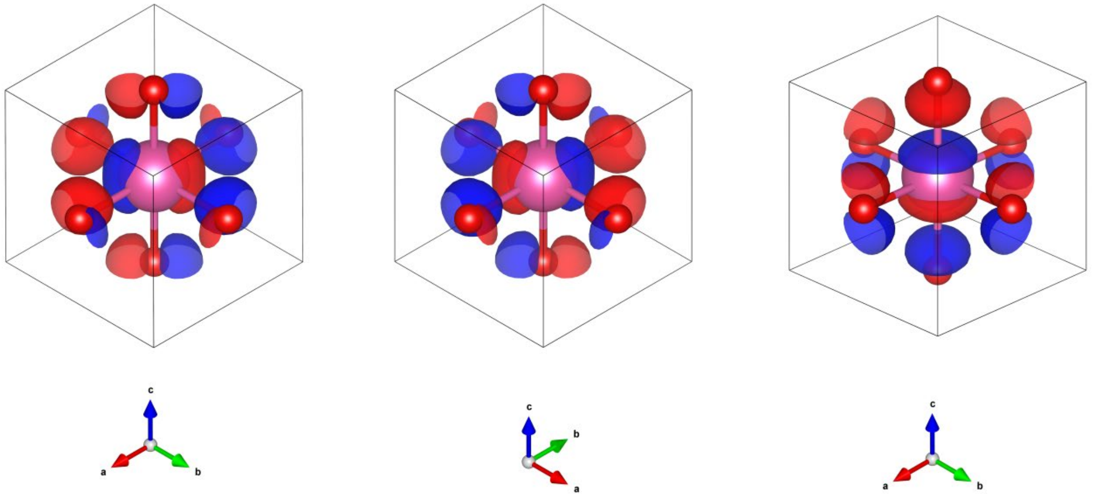

<u>Section 5.</u> U-6p “pushing from below” and SO splitting in δ-[UO_3]

Fig. S3. RDF for U^{6+} and O^0 (A) Top: U atom: Radial orbital density distributions D(r) (in atomic units; calculated for U^{6+} with ZORA-PBE using ADF) vs. the nuclear distance (in pm). The outer core closed shells are bold: 6s (dark blue), 6p (green: scalar-relativistic in full; $6p_{1/2}$ between 6s and 6p, dark & dashed, $6p_{3/2}$ larger than 6p, bright & dotted). The partially occupied valence orbitals are thin: inner 5f (blue), middle 6d (black), outer 7s (rose red) and 7p (beige: scalar-relativistic in full; inner $7p_{1/2}$ dark & dashed, outer $7p_{3/2}$ bright and dotted). The gray vertical dashed line indicates the U-O bond length of 208 pm. **(B) Bottom:** Similar, but without valence-Rydberg U-7s,7p; instead the orbitals of bonded O in reverse, starting at 208 pm: O-2s (lilac) and O-2p (red).

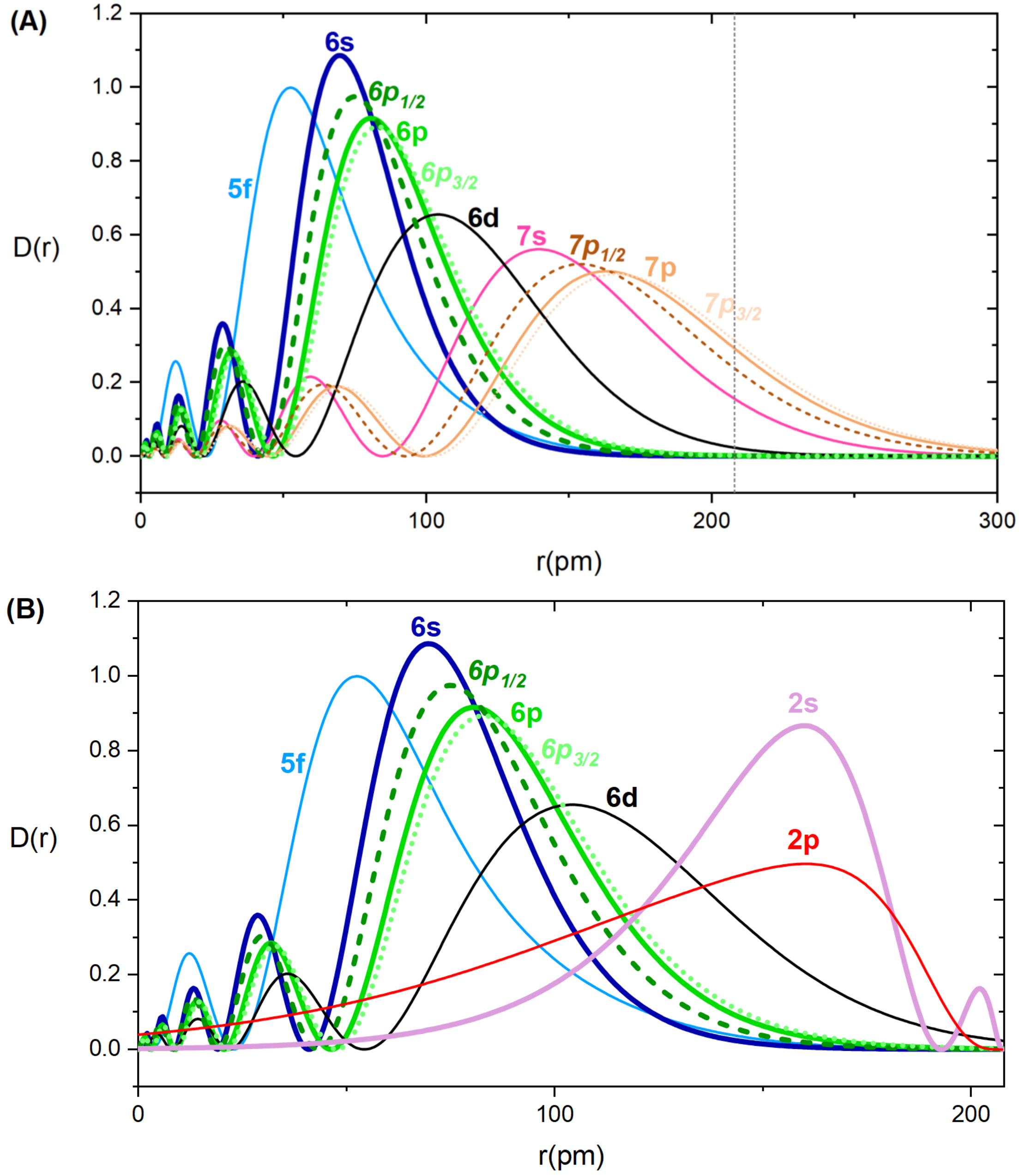

Fig. S4. Band structure of δ-[UO_3] (energies w.r.t. the lower Fermi edge, in eV). Scalar relativistic Kohn-Sham PBE approximation. Atomic core bands up to U-6s (only this highest one shown here) in dark blue; U-6p and O-2s semi-core bands in green and lilac; O-2p dominated valence band in red; conduction bands: the lowest ones of U-5f character in blue and the higher U-6d7s dominated ones in black. The U-6p and O-2s semi-core orbitals are strongly mixed; the O-2p is mixed with O-6d, U-5f and U-6p.

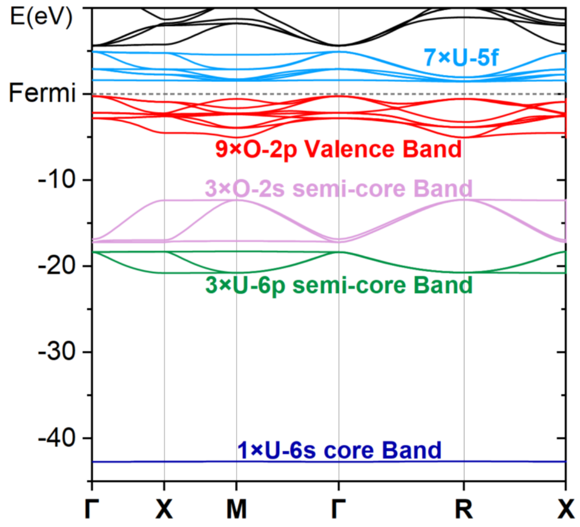

Fig. S5. Complete pCOHP of δ-[UO_3] (projected crystal orbital Hamiltonian population, energies in eV, zero at lower Fermi edge), showing the interactions of U-6s,6p,5f,6d,7s, 7p with O-2p. The interactions of the filled (semi-)core orbitals 6s and 6p are in bold dark blue and green.

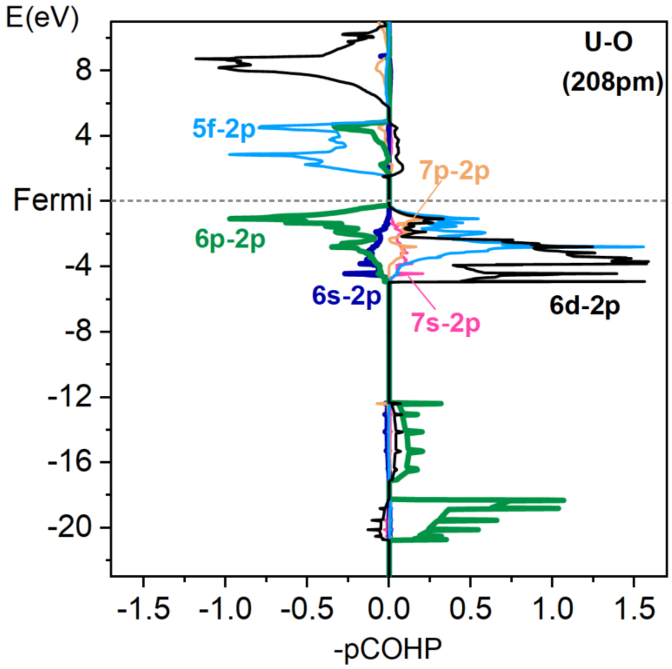

Table S12. Different overlaps S of a 7p STO function $7p_\zeta(r) = N_\zeta \cdot r^6 \cdot e^{-\zeta \cdot r}$, with the more compact $6p_{1/2}$ and the more extended $6p_{3/2}$ spinor of U at same center.

ζ(7p)	$6p_{1/2}$	$6p_{3/2}$
1.5	0.112	0.154
2.0	0.281	0.359
2.5	0.485	0.583
3.0	0.676	0.768
3.5	0.819	0.884
4.0	0.903	0.928

Fig. S6. Partial DOS for δ-[UO_3], from VASP and Lobster calculation.

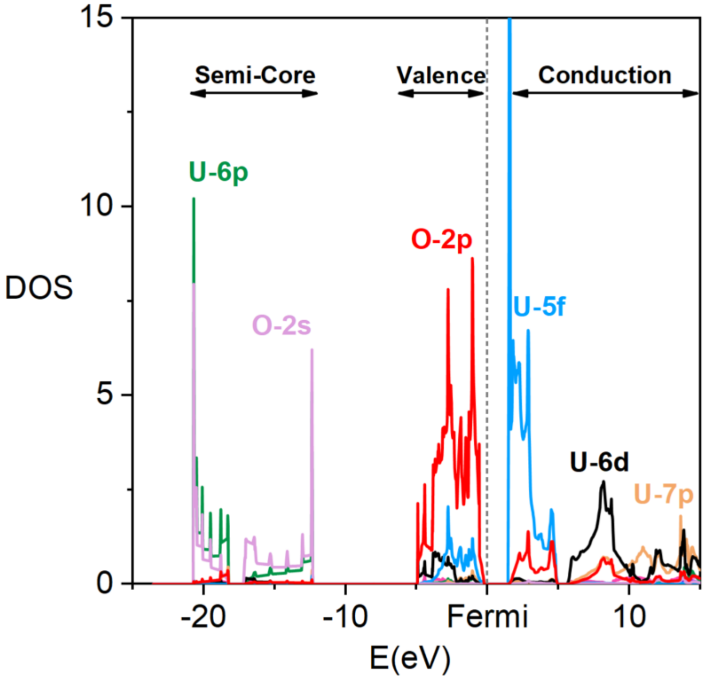

The band structure of δ-[UO_3] in Fig. S4 consists of four physically distinct sections. Our results correspond to the common ones, referred to in the introduction, but add some important new aspects. The four sections are: (i) The horizontal non-interacting **core** levels lying more than 40 eV below the Fermi edge, the highest one being U-6s. (ii) The weakly valence-active U-6p and O-2s **semi-core** levels leading to hybridized bands, in which bonding and antibonding effects largely compensate each other, the so-called degeneracy-driven non-bonding covalence.(*90*) (iii) The polar-covalent U-O bonding levels due to mixing of formally empty U^{6+}-5f,6d,7s,7p and formally filled O^{2-}-2p **valence** levels, caused by coordinative donation of O-2p pairs into U valence shell. (iv) Above the semi-conductor gap of about 2 eV there comes the U-5f dominated empty conduction band.

Fig. S7. Phase relations of interacting atomic orbitals at two different k-points in δ-[UO_3]. **(A) Left**: Translation symmetric Γ point: O-pπ/U-fπ, O-pσ/U-fσ, O-pσ/U-pσ interactions for all 3 equivalent components. In parentheses, the numbers of similarly interacting components at the X, M and R points. **(B) Right**: translation anti-symmetric R point: O-pπ/U-dπ, O-pσ/U-dσ, O-pσ/U-sσ, O-sσ/U-pσ with direction multiplicity. In parentheses, the numbers of similar interactions at the X and M points.

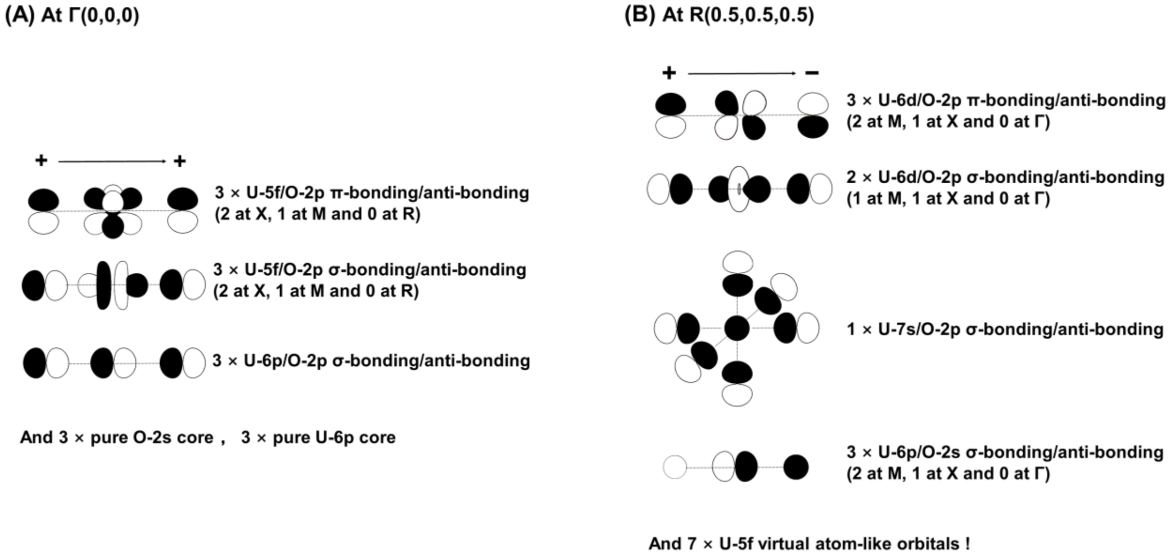

The projected crystal orbital Hamilton populations (pCOHP) in Fig. S5 clearly show the different interactions in sections (ii), (iii) and (iv). Applying a conventional projected density of states analysis (pDOS, see Fig. S6), it is easy to overlook the U-6p contribution in the valence shell because of the small amount of U-6p. But U-6p is in fact radially extended enough to interact with O-2p rather strongly (see Fig. S3). Even though U-6p mixing %age in the valence region is rather low, it can significantly push the O-2p energy level higher by Pauli-repulsion (see Fig. S5) and overlap interaction.

In order to demonstrate the two mechanisms for "U-6p outer core to push SO splitting into O-2p valence", we perform a frozen-U-$6p^6$-shell calculation (Fig. S8 left) using AMS-BAND, and compare it with a 6p-in-valence calculation (Fig. S8 right). Apparently, the different Pauli-repulsions by the U-$6p_{1/2}$ and U-$6p_{3/2}$ do not create the full SO splitting in the O-2p valence shell. One quarter is due to direct mixing of U-6p into the O-2p band (at the VBM at the Γ point; see Tables S7-9).

Wadt (*91*) had questioned Tatsumi & Hoffmann's insight concerning of U-6p influencing the bond angle of uranyl, because relativistic effective core potentials (RECP) with U-$6p^6$ in a frozen core also predict a linear structure. Obviously, U-6p admixture into the valence shell is not correctly simulated by his early crude effective core potential model, different U-$6p_{1/2}$ and U-$6p_{3/2}$ exert different Pauli-repulsions and cause the mentioned ¾ fraction of SO splitting, largest at the Γ point (1.35 eV of the total 1.76 eV, Fig. S8).

Fig. S8. Spin-orbit coupled (SOC) and scalar (SR) bands of δ-[UO_3], PBE calculations with AMS-BAND, energies in eV, with Zero at the lower Fermi edge. Red and blue lines are, respectively, the SR O-2p valence and U-5f conduction bands; the purple lines are the SOC bands. The SO splitting of the O-2p band at the VBM at the Γ point is indicated by a red double-arrow. **(A)** Frozen atomic U-$6s^2 6p_{1/2}^2 6p_{3/2}^4$ cores. **(B)** U-6s,$6p_{1/2,3/2}$ together with U-5f,6d,7s,7p and O-2s,2p in the optimized valence shell.

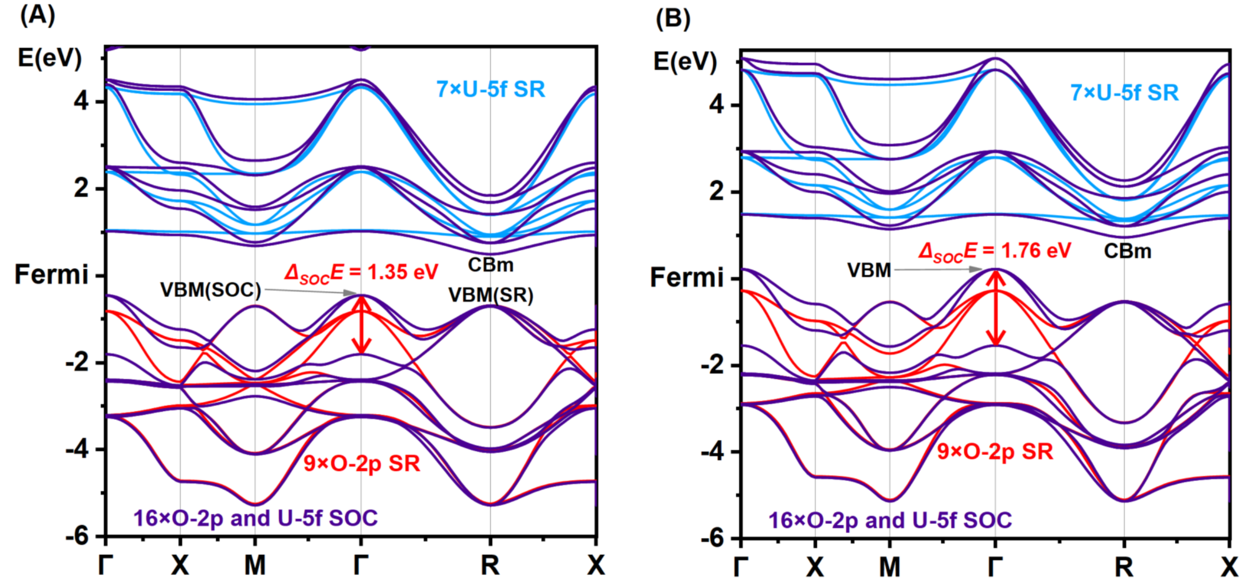

Fig. S9. All-electron band structure of δ-[UO_3], energies in eV, Zero at the lower Fermi edge, PBE approximation. **Left:** Scalar (SR) projected bands with O-2p in red, and (enhanced) U-5f in blue, U-6d in green (U-6p not indicated). **Right:** SR bands in red (valence O-2p type) and blue (conduction U-5f type) vs. the SO split bands (all in purple). The SO splitting at the VBM and CBm are highlighted by red double-arrows.

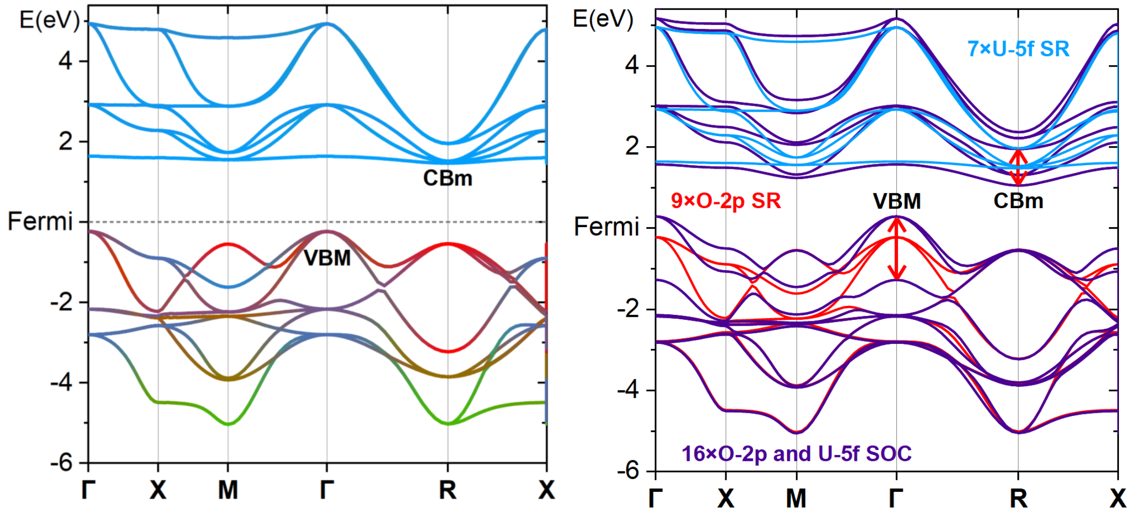

In Fig. S9 and Fig. S10, we compare the projected bands and SO splitting, to verify that O-2p dominated valence crystal orbitals show a significant SO effect only if admixture of U-6p semi-core orbitals is admitted. The U-5f,6d,7p SO coupling alone due to the dative O-2p→U-5f,6d,7p bonding causes a hardly visible SO splitting in the O-2p valence band.

A flat band of non-degenerate pure O_h-U-5f_{xyz}-a_{2u} character (upper region of Fig. S10) is the CBm. The other 6 U-5f type orbitals undergo varying degrees of crystal field splitting, which competes with SO coupling and reduces the latter. At the Γ point, the strong ligand field of O_h symmetry raises the T_{1u}* and T_{2u}* type U-5f orbitals, which are the anti-bonding counterparts of the σ/π U-5f/O-2p coordination bonds. Therefore, the weakest U-5f SO coupling in the conduction band is found at Γ. In contrast, at the R point, the translation symmetry allows g-type U-6d/O-2p bonding without u-type U-5f involvement (Fig. S7). Consequently, the conduction band orbitals are of dominant U-5f character with biggest SO splitting at the near-degenerate R point.

<u>Section 5a</u>. *U-6p Valence Admixture in Various Phases and under Pressure*

Figs. S10-S33 show the scalar-relativistic (SR) projected band structures, and the spin-orbit coupled (SOC) band structures, for all experimentally known and theoretically predicted [UO_3] phases and for [ThO_2], under various pressures (for the experimentally known ones under 0, 40, and 80 GPa; for the high-pressure phases predicted by Ma et al. ((*12*) under 80, 160, and 240 GPa). Our conclusion is that the SOC in the O-2p dominated valence band is determined by the U-6p semi-core admixture, throughout. The relevance of SOC in the high-pressure phases is further discussed in Section 7 below.

Fig. S10. Band structure of δ-[UO_3] at 0 GPa. **Left:** Projected SR bands, O-2p in red, U-5f in blue (in the valence band enhanced × 4), U-6p in green (enhanced × 12) (U-6d here not highlighted). **Right:** SR bands, O-2p type in red, U-5f type in blue, all SOC ones in purple. The SO splittings at VBM and CBm are highlighted by red double-arrows. PBE-approximation.

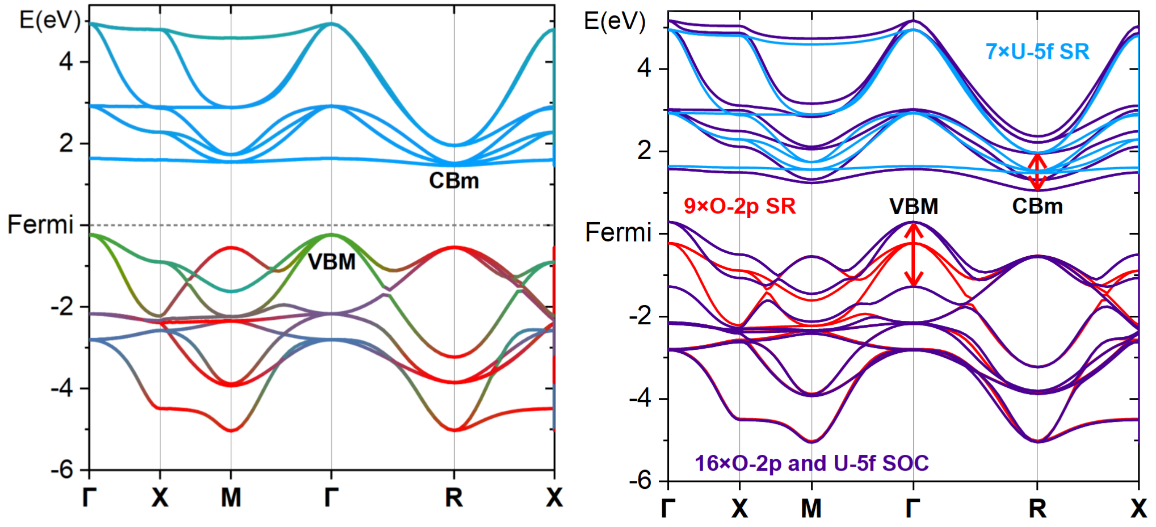

Fig. S11. Band structure of δ-[UO_3] at 40 GPa. See the legend of Fig. S10.

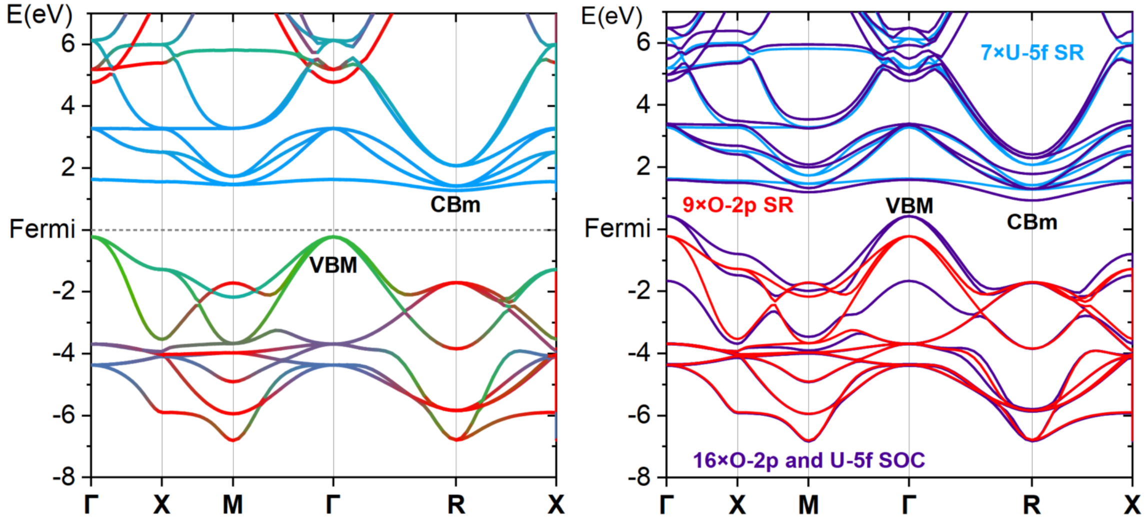

Fig. S12. Band structure of δ-[UO_3] at 80 GPa. See the legend of Fig. S10.

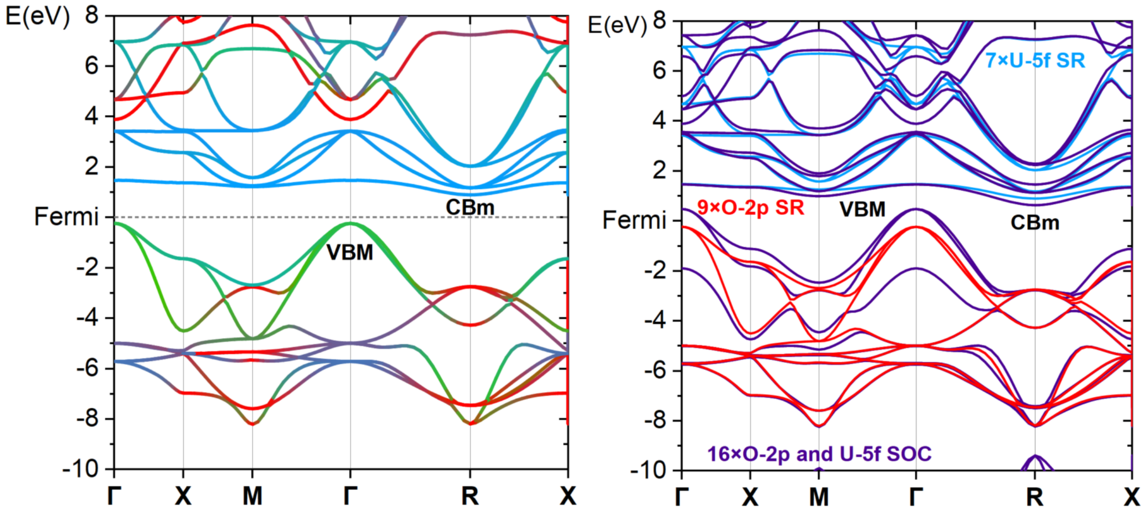

Fig. S13. Band structure of α-[UO_3] at 0 GPa. See the legend of Fig. S10.

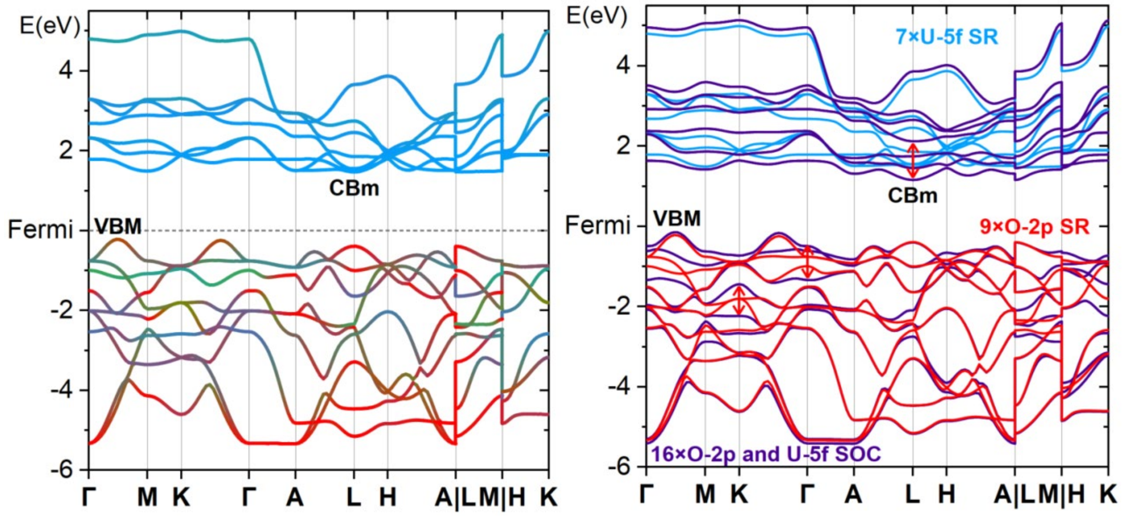

Fig. S14. Band structure of α-[UO_3] at 40 GPa. See the legend of Fig. S10.

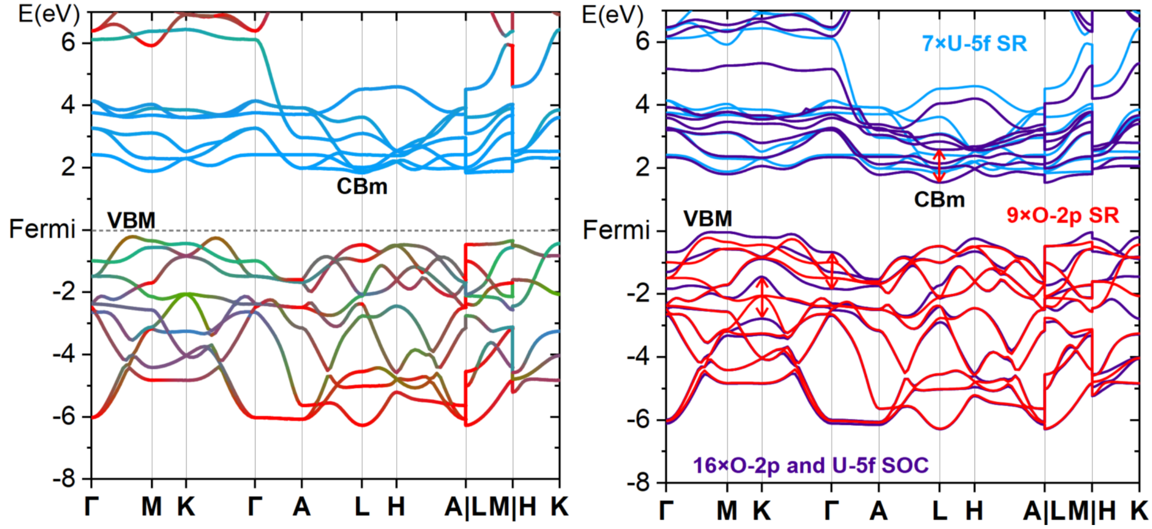

Fig. S15. Band structure of α-[UO_3] at 80 GPa. See the legend of Fig. S10.

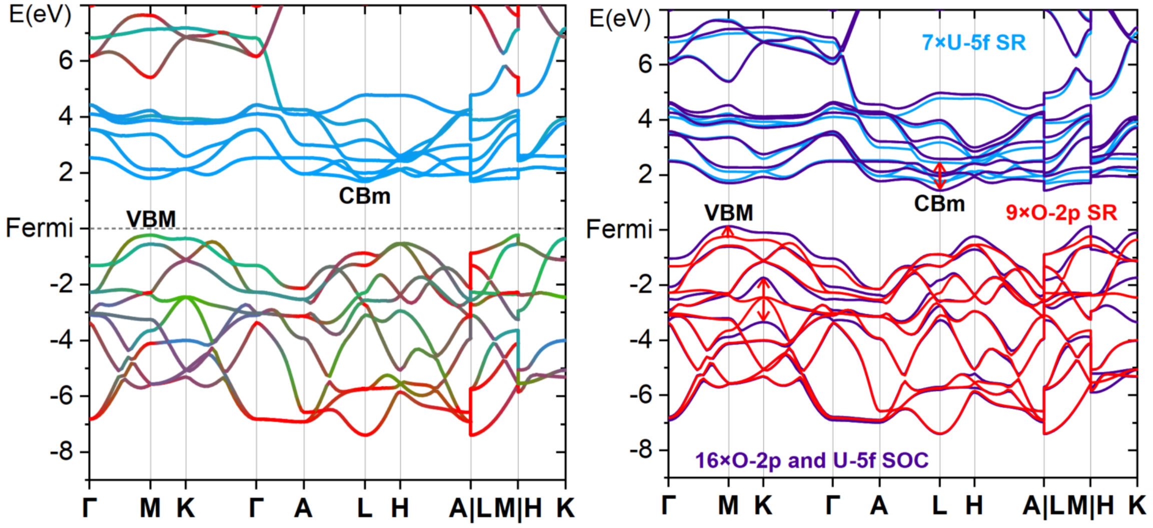

Fig. S16. Band structure of β-[UO_3] at 0 GPa. See the legend of Fig. S10.

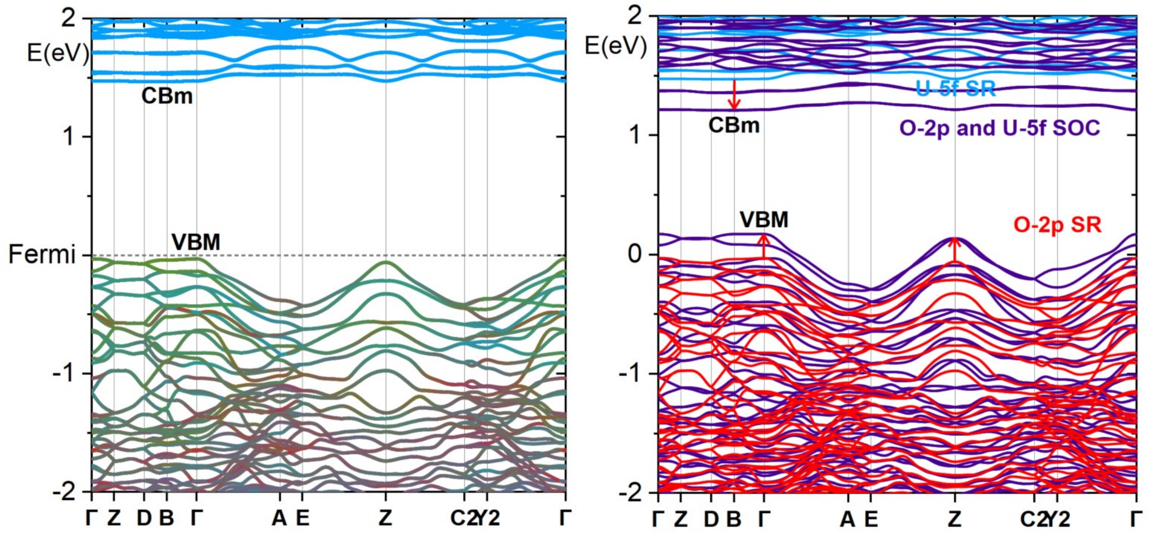

Fig. S17. Band structure of β-[UO_3] at 40 GPa. See the legend of Fig. S10.

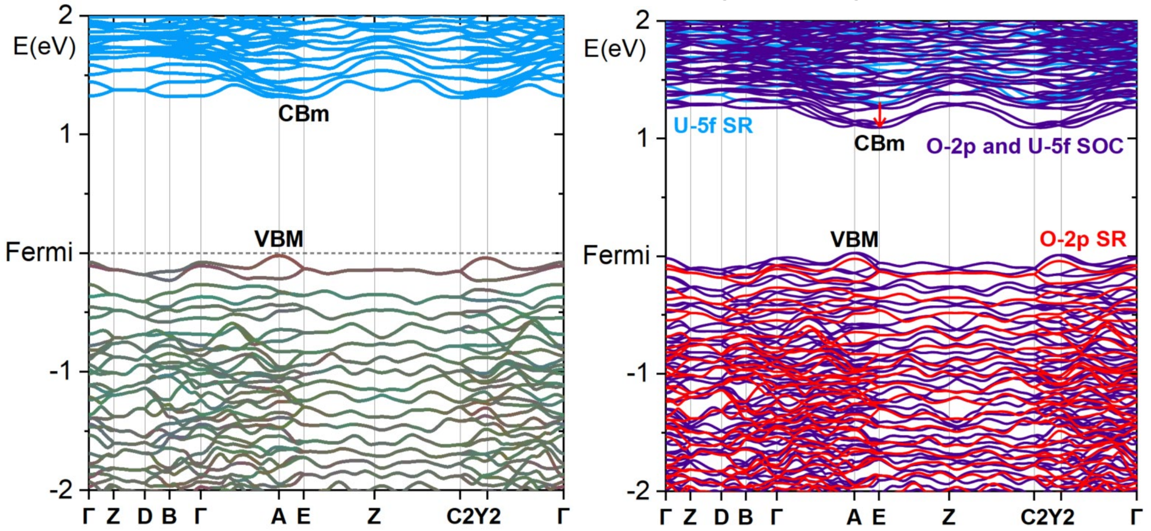

Fig. S18. Band structure of β-[UO_3] at 80 GPa. See the legend of Fig. S10.

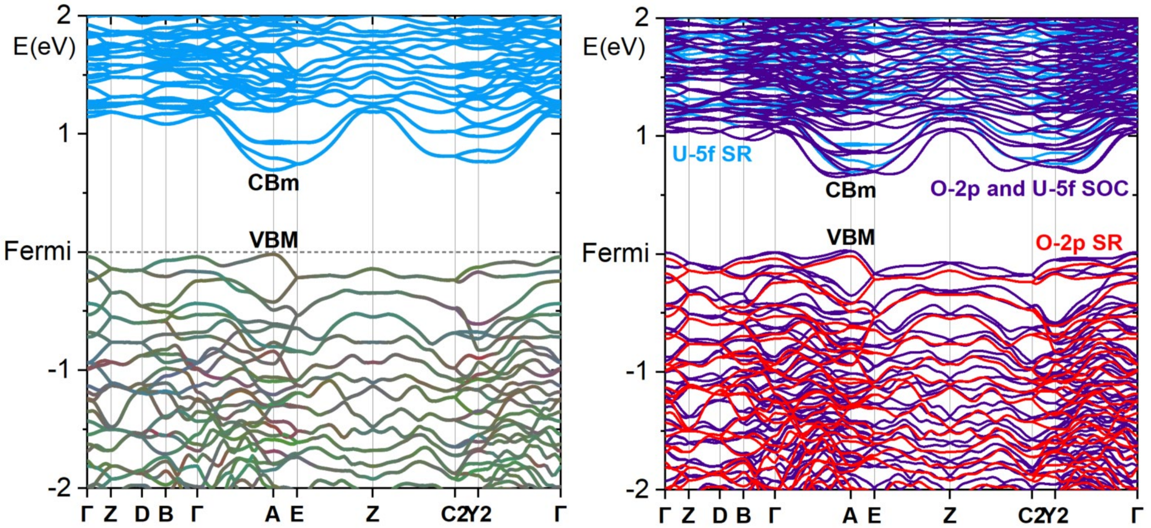

Fig. S19. Band structure of γ-[UO_3] at 0 GPa. See the legend of Fig. S10.

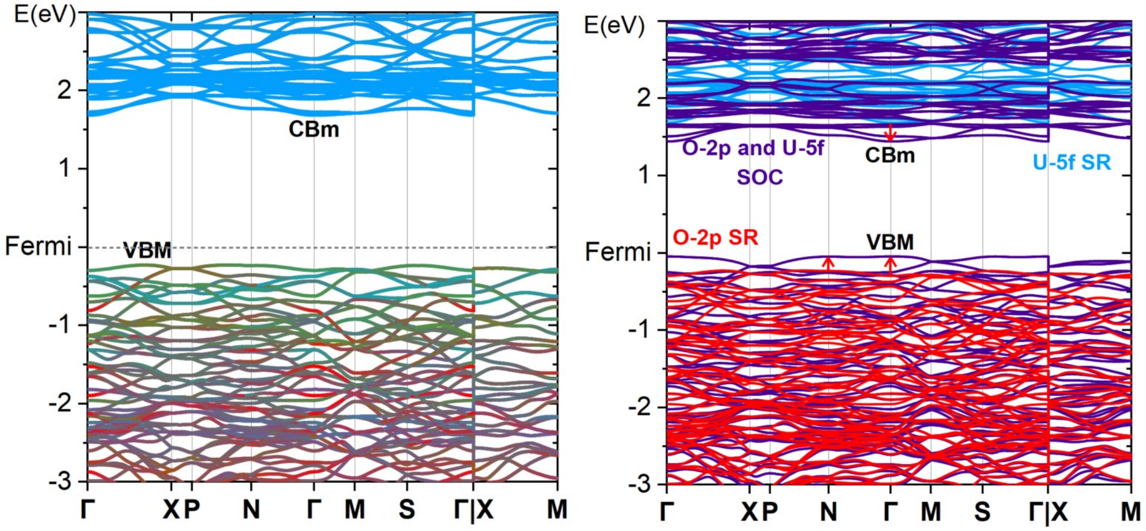

Fig. S20. Band structure of γ-[UO_3] at 40 GPa. See the legend of Fig. S10.

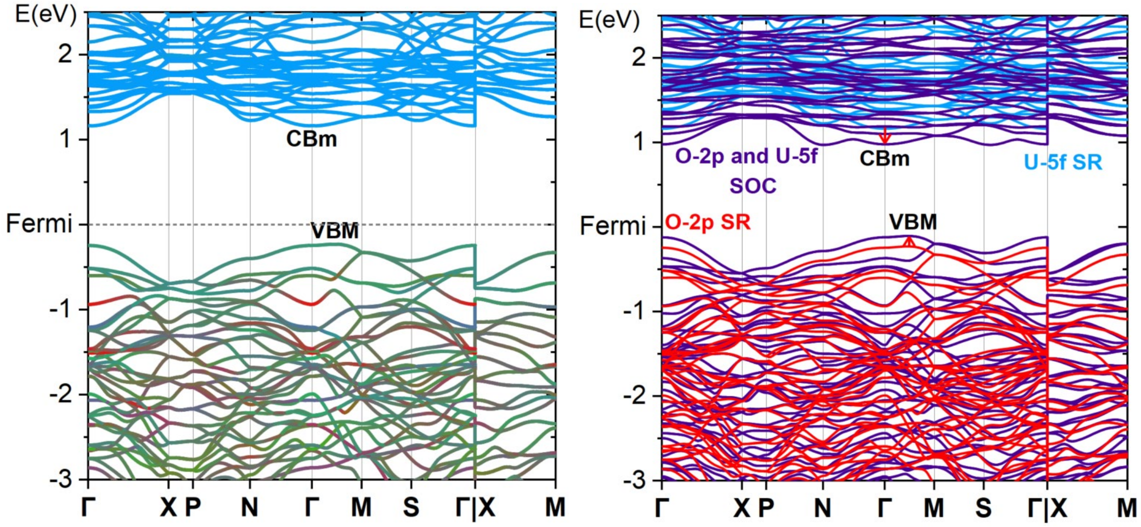

Fig. S21. Band structure of γ-[UO_3] at 80 GPa. See the legend of Fig. S10.

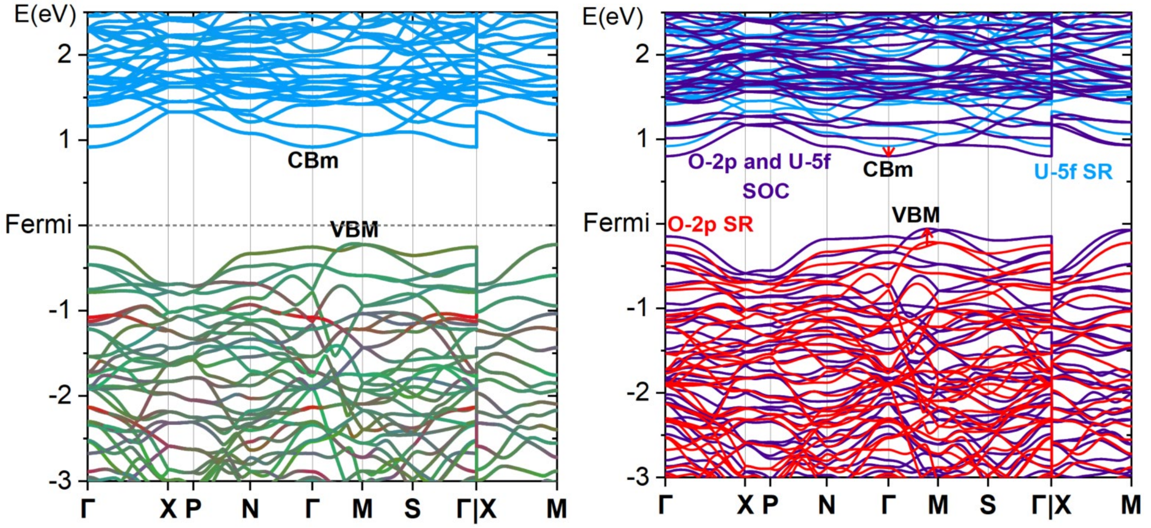

Fig. S22. Band structure of η-[UO_3] at 0 GPa. See the legend of Fig. S10.

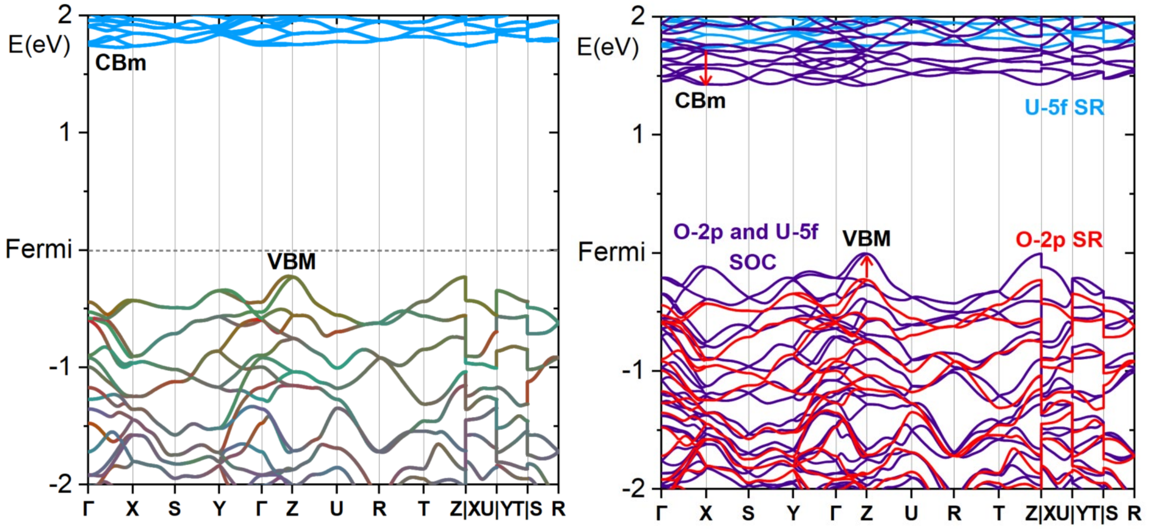

Fig. S23. Band structure of η-[UO_3] at 40 GPa. See the legend of Fig. S10.

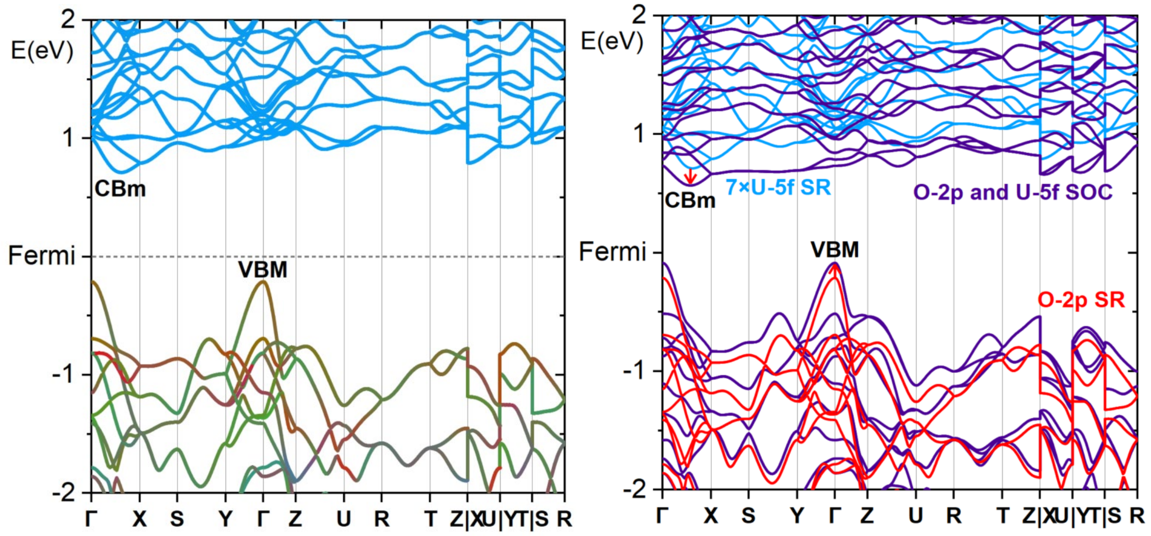

Fig. S24. Band structure of η-[UO_3] at 80 GPa. See the legend of Fig. S10.

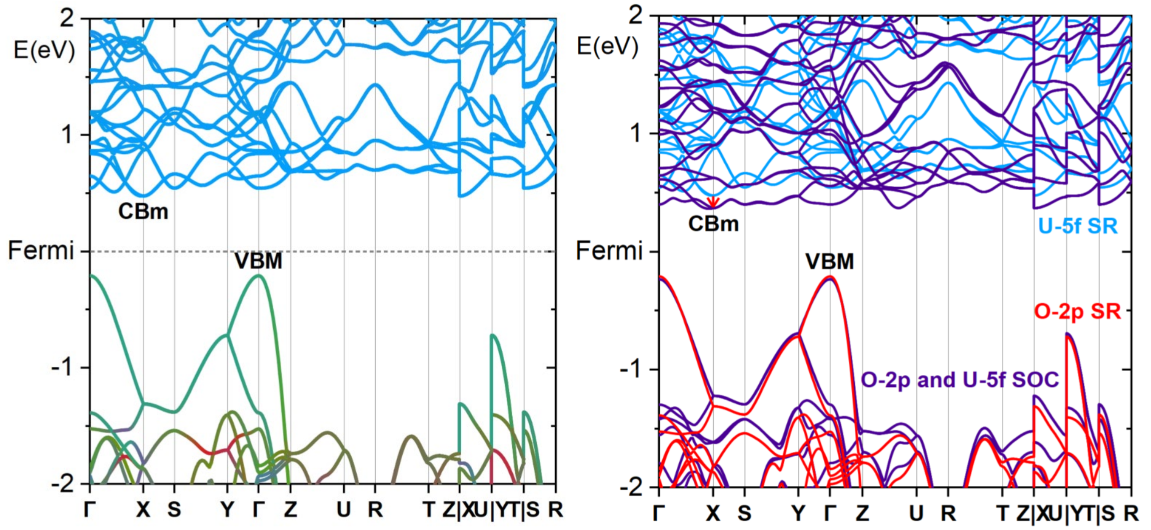

Fig. S25. Band structure of [ThO_2] at 0 GPa. See the legend of Fig. S10.

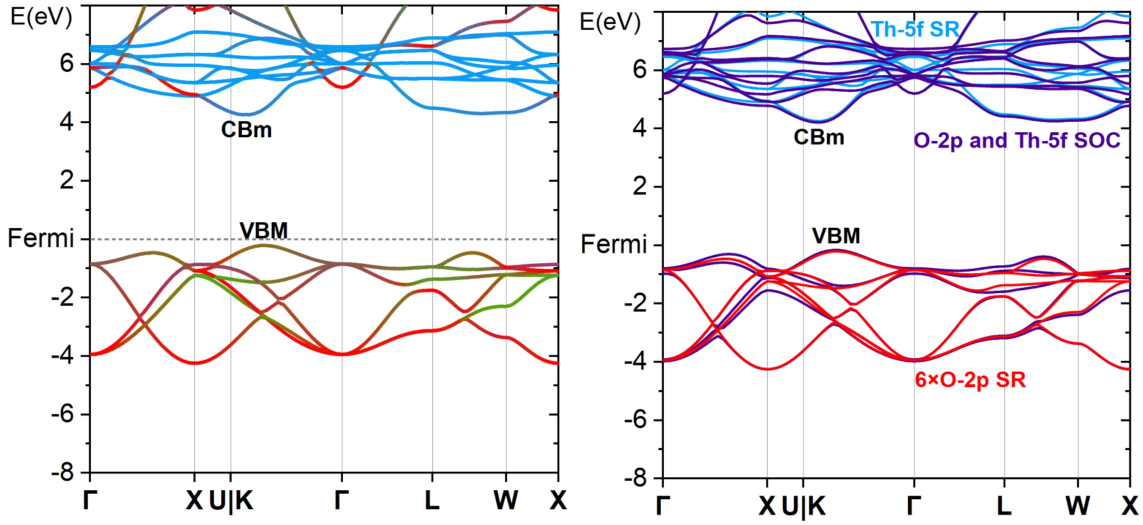

Fig. S26. Band structure of [ThO_2] at 40 GPa. See the legend of Fig. S10.

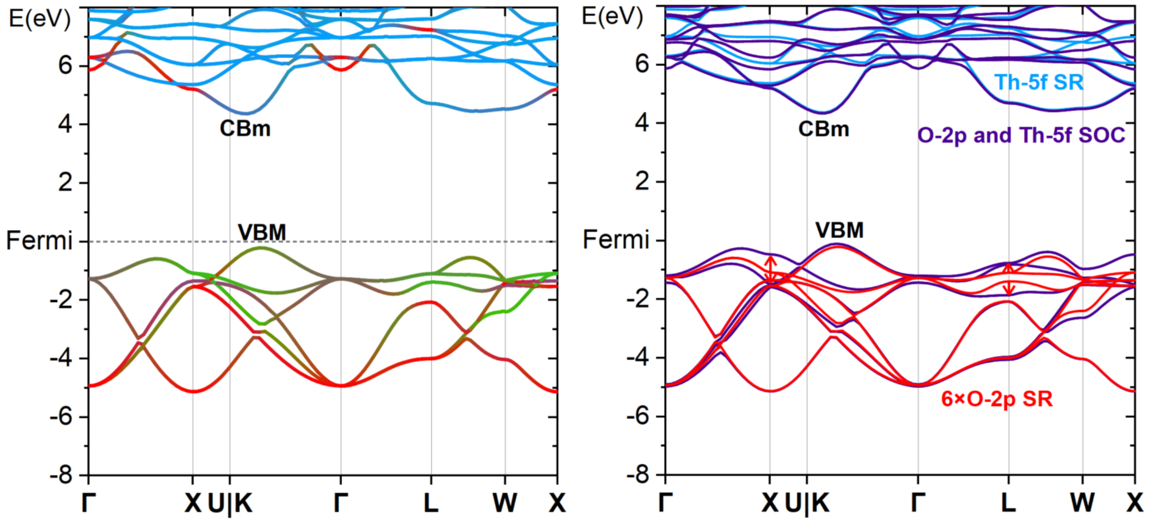

Fig. S27. Band structure of [ThO_2] at 80 GPa. See the legend of Fig. S10.

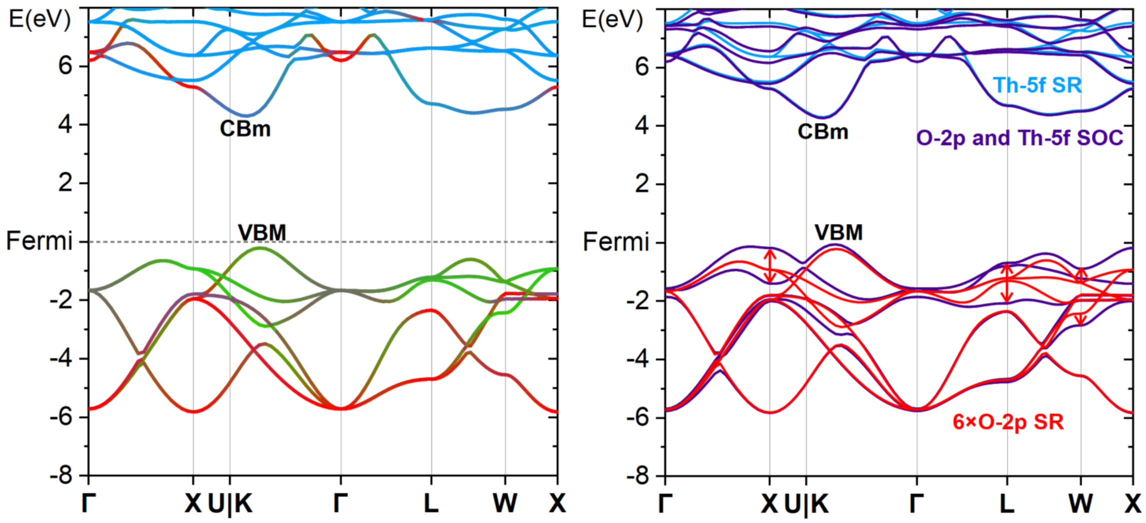

Fig. S28. Band structure of P6$_3$/mmc x-[UO_3] at 80 GPa. See the legend of Fig. S10.

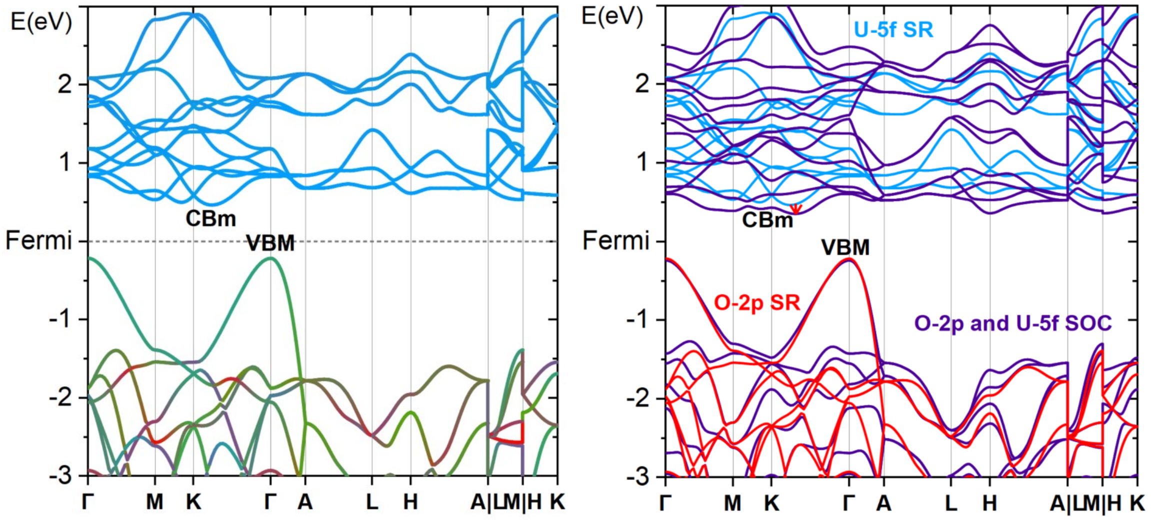

Fig. S29. Band structure of P6$_3$/mmc x-[UO$_3$] at 160 GPa. See the legend of Fig. S10.

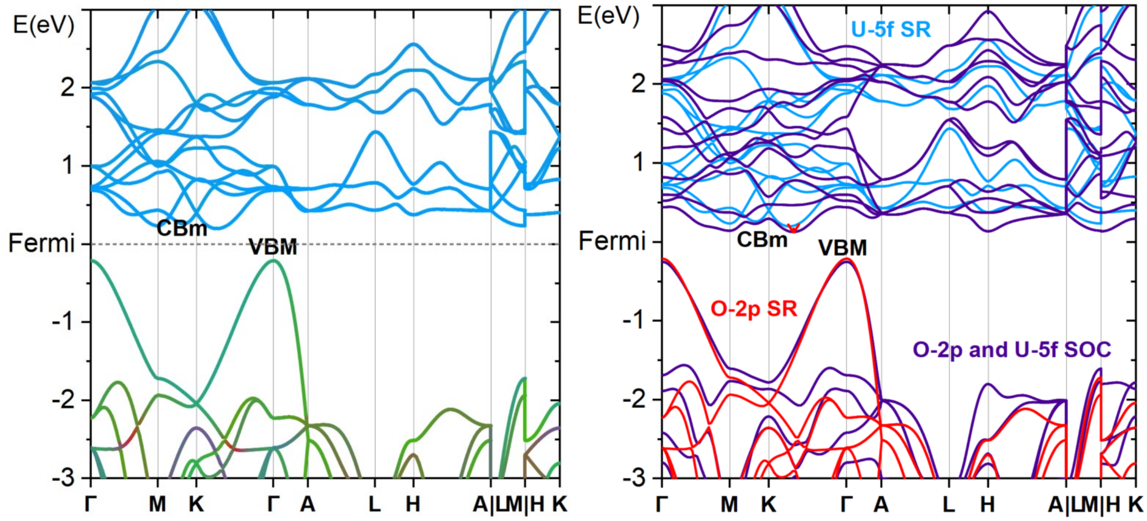

Fig. S30. Band structure of P6$_3$/mmc x-[UO$_3$] at 240 GPa. See the legend of Fig. S10.

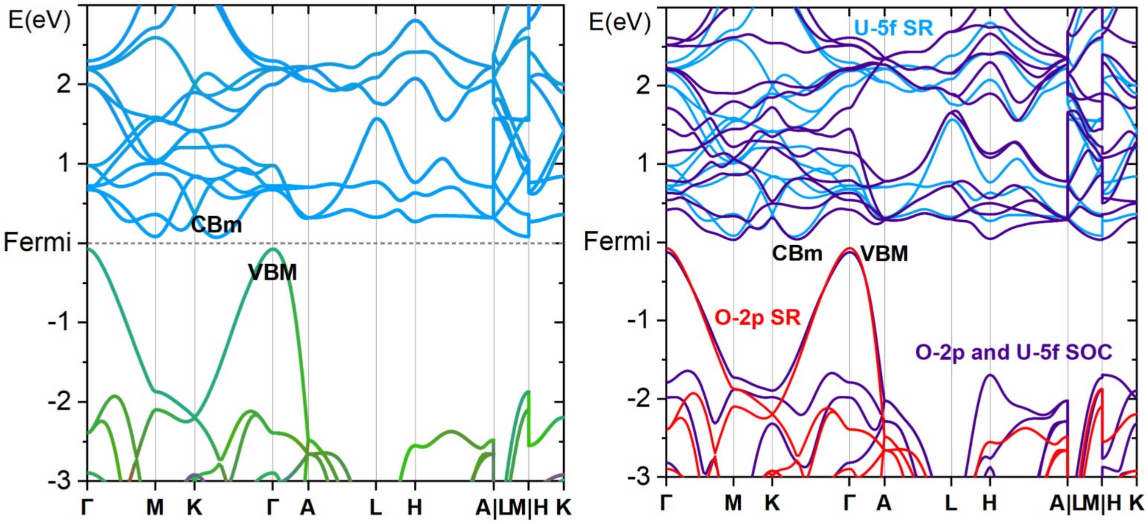

Fig. S31. Band structure of Fm$\overline{3}$m y-[UO_3] at 80 GPa. See the legend of Fig. S10. Bottom: HSE density functional instead of PBE.

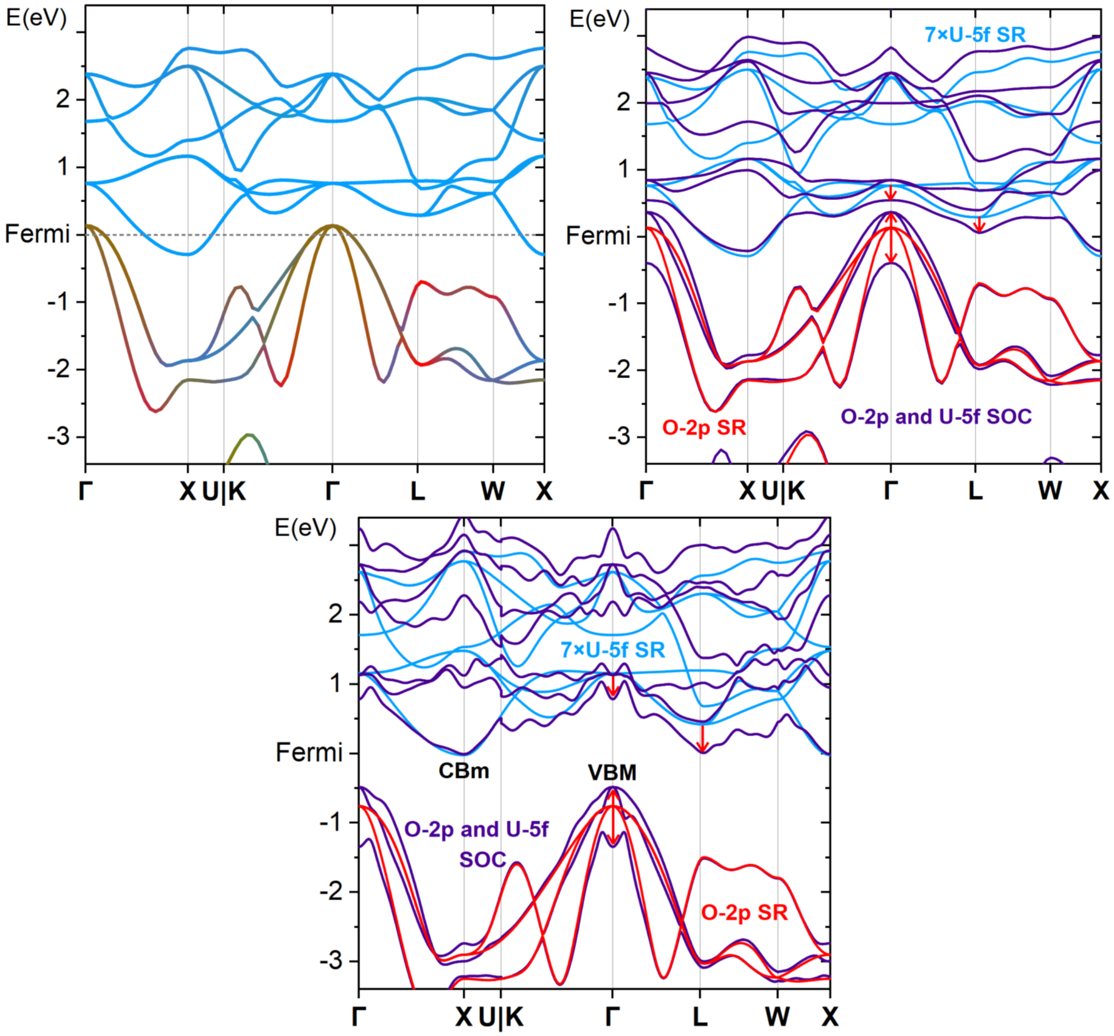

Fig. S32. Band structure of Fm$\overline{3}$m y-[UO_3] at 160 GPa. See the legend of Fig. S10. Bottom: HSE density functional instead of PBE.

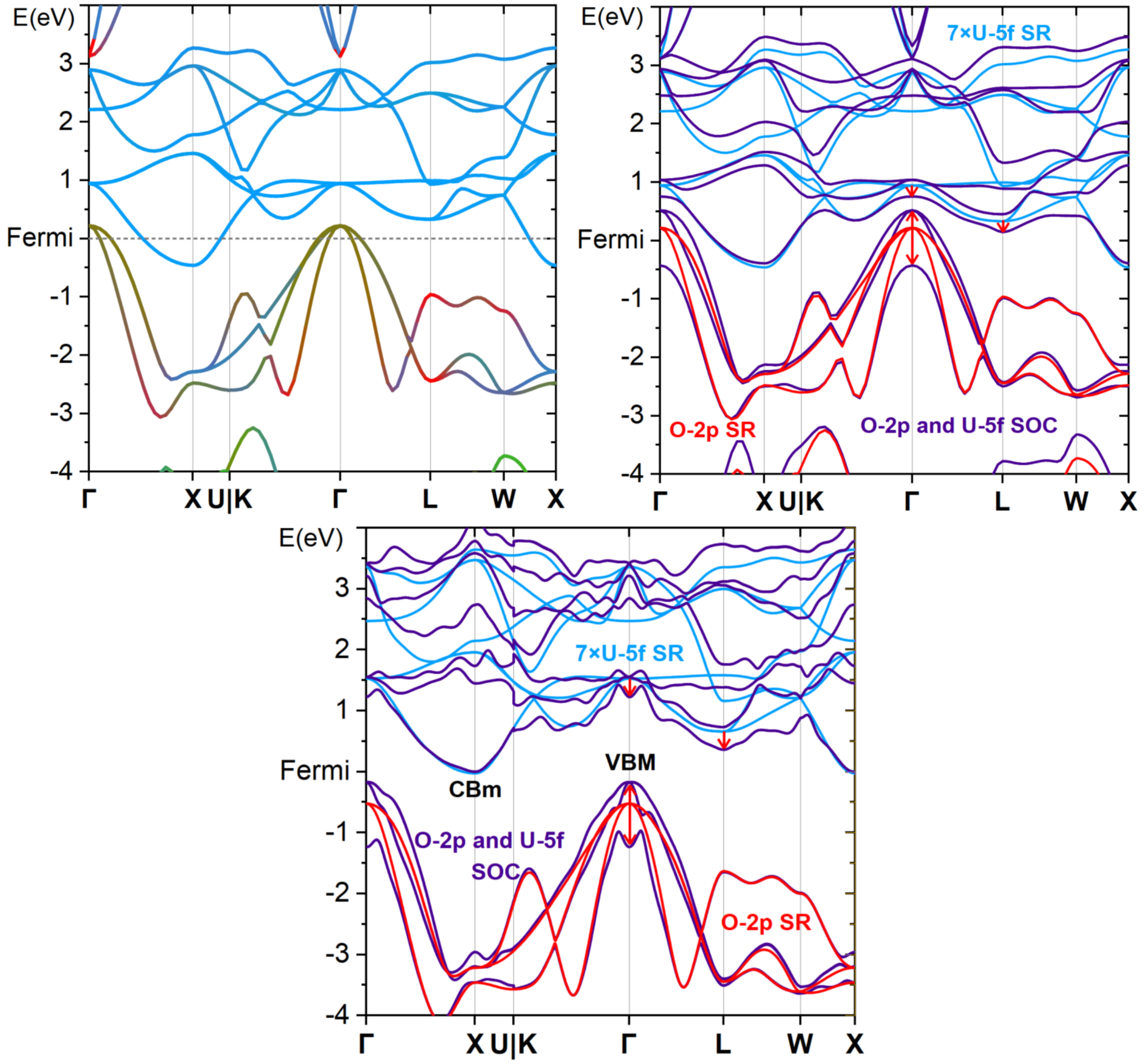

Fig. S33. Band structure of Fm$\overline{3}$m y-[UO_3] at 240 GPa. See the legend of Fig. S10. Bottom: HSE density functional instead of PBE.

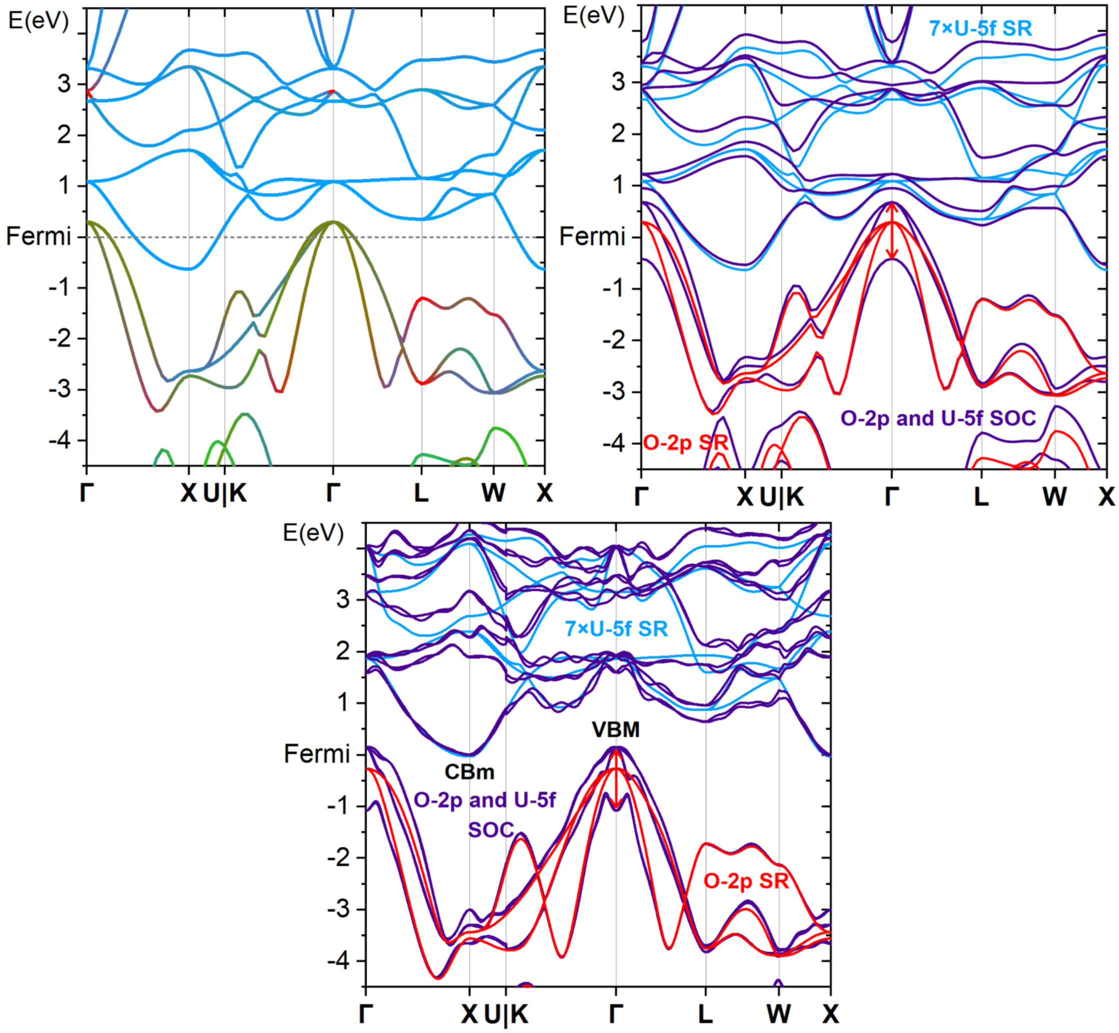

Section 6. **From Molecular $U(OH)_6$ to Solid δ-[UO_3]: SO Coupling vs. Crystal Field Splitting**

In this section, we apply a simple molecular model to better understand the relation between crystal field (CF) and SO Coupling (SOC) effects around the U atom. In general, a group of degenerate orbitals such as U-6p,5f,6d is split by a low-symmetry CF, and also by SOC, but the two perturbations interfere with each other. We define the level splitting changes by ligand field (CF) and SO coupling (SOC) as:

$$\Delta_{CF}E = E_{CF}^{max} - E_{CF}^{min}$$

$$\Delta_{SOC}E = E_{SOC}^{max} - E_{SOC}^{min}$$

$$\Delta_{CF+SOC}E = E_{CF+SOC}^{max} - E_{CF+SOC}^{min}$$

$$\Delta_{SOC}\Delta E = \Delta_{CF+SOC}E - \Delta_{CF}E$$

P-Levels: In cases of high geometric symmetry, such as O_h for U in the δ-[UO_3] phase, the degeneracy of p-states is not lifted by the CF, and the SOC is not attenuated in the crystal lattice:

$$\Delta_{SOC}\Delta E = \Delta_{CF+SOC}E = \Delta_{SOC}E$$

while the degeneracy of d- and f-states is broken. I.e. in δ-[UO_3] the bare-atom like triply-degenerate semi-core 6p and the 6p-mixed valence orbitals keep their full SOC despite the CF (Fig. S34A), which may only shift the levels. In contrast, D_{4h} and lower symmetries of the other [UO_3] phases restrain the SOC splitting (Fig. S34B).

Fig. S34a. Sketch of atomic p-levels, shifted and split by a crystal field (CF) and by spin-orbit coupling (SOC) with parameter **λ**. **(A) Top:** O_h symmetry (e.g. [U(-O-)$_6$] type), CF parameter ε. **(B) Bottom:** weak symmetry breaking CF of strength $\delta(D_{4h})$, e.g. [U(=O)$_2$(··O··)$_4$] type.

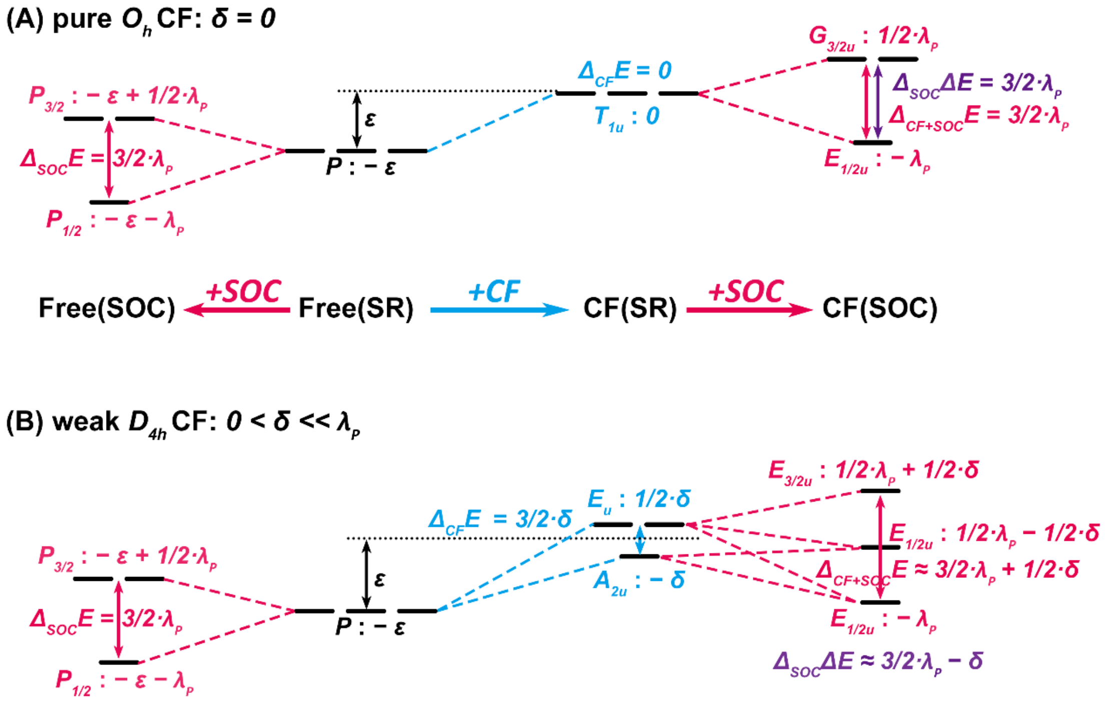

Concerning the SOC of an atomic p-level, we choose as basis $(p_x \cdot \beta\ ,\ p_y \cdot \beta\ ,\ p_z \cdot \alpha)$, yielding the SOC-Hamiltonian matrix:

$$\boldsymbol{\hat{H}_{SOC}} = \frac{\boldsymbol{\lambda}}{\mathbf{2}} \cdot \begin{bmatrix} \mathbf{0} & \boldsymbol{i} & \mathbf{-1} \\ \boldsymbol{-i} & \mathbf{0} & \boldsymbol{-i} \\ \mathbf{-1} & \boldsymbol{i} & \mathbf{0} \end{bmatrix}$$

with eigen-solutions $\boldsymbol{E_1} = -\boldsymbol{\lambda}$ for $(\mathbf{1}, -\boldsymbol{i}, \mathbf{1})^{\dagger}/\sqrt{\mathbf{3}}$ and $\boldsymbol{E_{2,3}} = +\boldsymbol{\lambda}/\mathbf{2}$ for $(\mathbf{1}, \boldsymbol{i}, \mathbf{0})^{\dagger}/\sqrt{\mathbf{2}}$, and $(-\mathbf{1}, \boldsymbol{i}, \mathbf{2})^{\dagger}/\sqrt{\mathbf{6}}$ (in general $\boldsymbol{\lambda} > 0$ for 1-electron p-levels).

The Hamiltonian matrix for a D_{4h} CF perturbation along the z-axis is:

$$\boldsymbol{\hat{H}_{CF}} = \boldsymbol{\varepsilon} \cdot \mathbf{1} + \boldsymbol{\delta} \cdot \begin{bmatrix} \frac{1}{2} & \mathbf{0} & \mathbf{0} \\ \mathbf{0} & \frac{1}{2} & \mathbf{0} \\ \mathbf{0} & \mathbf{0} & \mathbf{-1} \end{bmatrix}$$

where $\boldsymbol{\varepsilon}$ means the overall shift by the totally symmetric component of the CF. The resulting eigen-values of $\boldsymbol{\hat{H}_{CF}} + \boldsymbol{\hat{H}_{SOC}}$ are:

$$\boldsymbol{E_1} = \boldsymbol{\varepsilon} + \mathbf{1/2}(\boldsymbol{\lambda} + \boldsymbol{\delta}) \quad (\boldsymbol{E_{3/2u}})$$

$$\boldsymbol{E_{2,3}} = \boldsymbol{\varepsilon} - \frac{\mathbf{1}}{\mathbf{4}}\left[(\boldsymbol{\delta} + \boldsymbol{\lambda}) \pm \sqrt{9\boldsymbol{\delta}^2 - 6\boldsymbol{\delta}\boldsymbol{\lambda} + 9\boldsymbol{\lambda}^2}\right] \quad ((\boldsymbol{E_{1/2u}})$$

$\boldsymbol{E_1}$, $\boldsymbol{E_2}$, and $\boldsymbol{E_3}$ as a function of $\boldsymbol{\delta}/\boldsymbol{\lambda}$ are shown in **Figure 35a**.

For a weak CF in comparison to strong SOC, $|\boldsymbol{\delta}| \ll \boldsymbol{\lambda}$, this can be approximated and simplified, to order O($|\boldsymbol{\delta}|$), by :

$$\boldsymbol{E_{1,2}} = \boldsymbol{\varepsilon} + \frac{\mathbf{1}}{\mathbf{2}}\boldsymbol{\lambda} \pm \frac{\mathbf{1}}{\mathbf{2}}\boldsymbol{\delta}$$

$$\boldsymbol{E_3} = \boldsymbol{\varepsilon} - \boldsymbol{\lambda}$$

The splittings by pure SO coupling, and by a pure CF are, respectively, $3/2 \cdot \boldsymbol{\lambda}$ and $3/2 \cdot \boldsymbol{\delta}$. Strong SO coupling reduces a small D_{4h} CF splitting from 3/2 δ to about 1 δ .

Vice versa for small SO coupling in comparison to a strong CF, $\boldsymbol{\lambda} \ll |\boldsymbol{\delta}|$, the eigenvalues can be approximated and simplified, to O($\boldsymbol{\lambda}$), by :

$$\boldsymbol{E_{a,b}} = \boldsymbol{\varepsilon} + \frac{\mathbf{1}}{\mathbf{2}}\boldsymbol{\delta} \pm \frac{\mathbf{1}}{\mathbf{2}}\boldsymbol{\lambda}$$

$$\boldsymbol{E_c} = \boldsymbol{\varepsilon} - \boldsymbol{\delta}$$

In the case of a strong D_{4h} CF, the pure SO splitting of $^3/_2 \cdot \boldsymbol{\lambda}$ is reduced by about $1 \cdot \lambda$.

Fig. S34b. Sketch of atomic d and f levels, shifted and split by an O_h crystal field (CF) and by spin-orbit coupling λ (SOC). **(A) Top:** d-levels, CF parameters ε and δ. **(B) Bottom:** f-levels, CF parameters ε, δ_1 and δ_2.

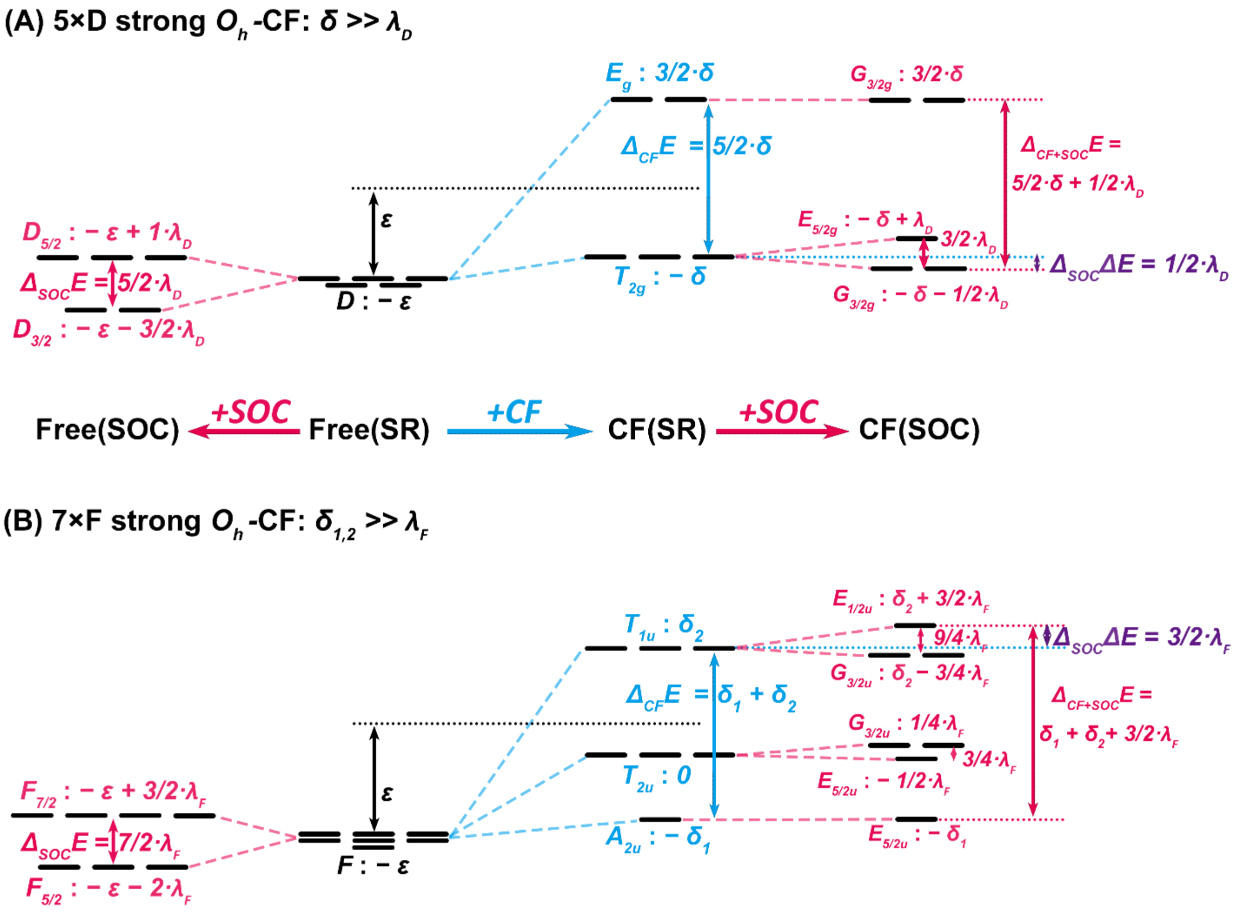

D Levels: d and f levels are split already by the high-symmetric O_h CF. The d levels are found in the textbooks (e.g. (*92*)), where ε, λ and δ now refer to the SOC and O_h-CF parameters of the d-orbitals (assuming the approximation of same radial orbital functions for the different states):

$$\boldsymbol{E_1 = \varepsilon - \delta + \lambda \quad (E_{5/2g})}$$

$$\boldsymbol{E_2 = \varepsilon + \frac{3}{2}\delta + \frac{5}{4}a \quad (G_{3/2g})}$$

$$\boldsymbol{E_3 = \varepsilon - \delta - \frac{1}{2}\lambda - \frac{5}{4}a \quad (G_{3/2g})}$$

with $$\boldsymbol{a = \sqrt{\delta^2 + 2/5\delta\lambda + \lambda^2} - (\delta + 1/5\lambda)}$$

$\boldsymbol{E_1}$, $\boldsymbol{E_2}$, and $\boldsymbol{E_3}$ as a function of $\boldsymbol{\delta/\lambda}$ are shown in **Figure 35b**.

For strong CF and weak SOC, $\boldsymbol{\lambda} \ll |\boldsymbol{\delta}|$,

$$\boldsymbol{E_1 = \varepsilon + \frac{3}{2}\delta} \qquad (\boldsymbol{G_{3/2g}})$$

$$\boldsymbol{E_2 = \varepsilon - \delta + \lambda} \quad (\boldsymbol{E_{5/2g}})$$

$$\boldsymbol{E_3 = \varepsilon - \delta - \frac{1}{2}\lambda} \quad (\boldsymbol{G_{3/2g}})$$

that is, the atomic SO splitting of 5/2·λ is reduced to 3/2·λ for the spatial triplet ($\boldsymbol{E}_2[E_{5/2g}]$ & $\boldsymbol{E}_3[G_{3/2g}]$), while the spatial doublet ($\boldsymbol{E}_1[G_{3/2g}]$) is not split at all.

For strong SOC and a weak CF, $|\boldsymbol{\delta}| \ll \boldsymbol{\lambda}$,

$$\boldsymbol{E_1 = \lambda + 3/2\delta} \quad (\boldsymbol{G_{3/2g}})$$

$$\boldsymbol{E_2 = \lambda - \delta} \quad (\boldsymbol{E_{5/2g}})$$

$$\boldsymbol{E_3 = -3/2\lambda - \delta} \quad (\boldsymbol{G_{3/2g}}).$$

F Levels: f levels in an O_h CF were considered by Atanasov et al.(*93, 94*), where CF parameters ε, δ_1 and δ_2 occur:

$$\boldsymbol{E_1 = \varepsilon + \delta_2 + 3/2\lambda} \quad (\boldsymbol{E_{1/2u}})$$

$$\boldsymbol{E_{2,4} = \varepsilon + \frac{\delta_2}{2} - \frac{1}{4}\lambda \pm \frac{1}{2}\sqrt{{\delta_2}^2 - 2\delta_2\lambda + (7/2 \cdot \lambda)^2}} \quad (\boldsymbol{G_{3/2u}})$$

$$\boldsymbol{E_{3,5} = \varepsilon - \frac{\delta_1}{2} - \frac{1}{4}\lambda \pm \frac{1}{2}\sqrt{{\delta_1}^2 - \delta_1\lambda + (7/2 \cdot \lambda)^2}} \quad (\boldsymbol{E_{5/2u}})$$

$\boldsymbol{E_1}$, $\boldsymbol{E_{2,4}}$, and $\boldsymbol{E_{3,5}}$ as a function of $\boldsymbol{\delta/\lambda}$ are shown in **Figure 35c**.

For strong CF and weak SOC, $\boldsymbol{\lambda} \ll \boldsymbol{\delta_1}, \boldsymbol{\delta_2}$,

$$\boldsymbol{E_1 = \varepsilon + \delta_2 + \frac{3\lambda}{2}} \quad (\boldsymbol{E_{1/2u}})$$

$$\boldsymbol{E_2 \approx \varepsilon + \delta_2 - \frac{3\lambda}{4}} \quad (\boldsymbol{G_{3/2u}})$$

$$\boldsymbol{E_3 \approx \varepsilon - \frac{\lambda}{2}} \quad (\boldsymbol{E_{5/2u}})$$

$$\boldsymbol{E_4 \approx \varepsilon + \frac{\lambda}{4}} \quad (\boldsymbol{G_{3/2u}})$$

$$\boldsymbol{E_5 \approx \varepsilon - \delta_1} \quad (\boldsymbol{E_{5/2u}})$$

Figures S35a-S35d show the full variation of the energy splittings jointly caused by CF and SOC for the P, D and F shells. Obviously, the quenching influence of the CF on the SO splitting increases from the atomic p-orbitals to the d-orbitals and more so to the f-orbitals.

Fig. S35a. Orbital energy levels of an SO split P-shell (SO splitting energy parameter $\boldsymbol{\lambda}$, whose value is fixed to 1) in a D_{4h} crystal field (CF splitting energy parameter $\boldsymbol{\delta}$). **Left:** The 3 levels in Blue, Green ($2\times E_{1/2u}$,) and Red ($1\times E_{3/2}u$). The energy levels without SOC are in Black (dashed): A_{2u} and E_u become degenerate for zero D_{4h} CF (T_{1u} at O_h symmetry). **Right:** Energy shift due to SOC in the CF, $\Delta_{SOC}\Delta E$, between the two limits, 1.5 $\boldsymbol{\lambda}$ for zero D_{4h} CF, and 0.5 $\boldsymbol{\lambda}$ for large CF.

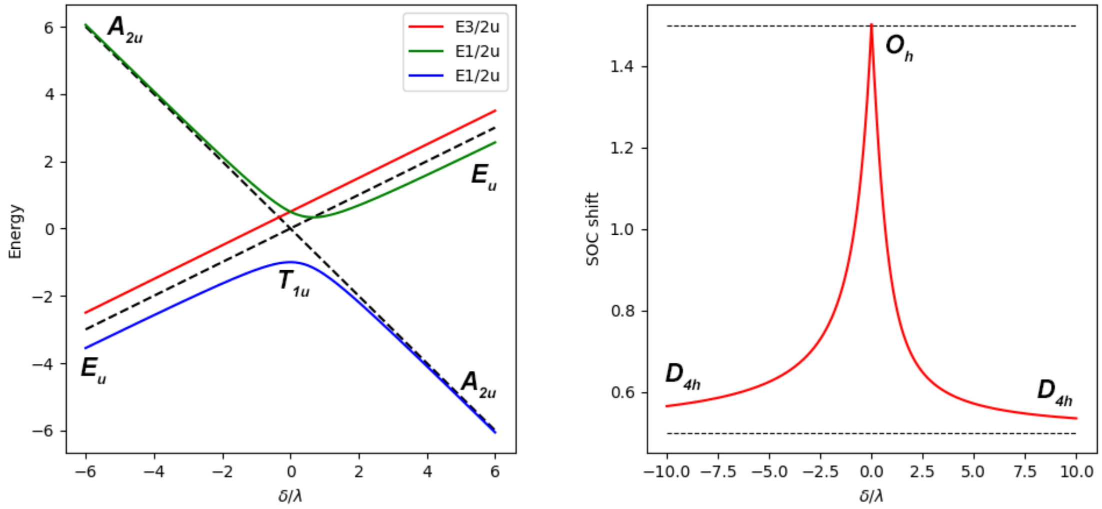

Fig. S35b. Orbital energy levels of an SO split D-shell (SO splitting energy parameter $\boldsymbol{\lambda}$, whose value is fixed to 1) in a O_h crystal field (CF splitting energy parameter $\boldsymbol{\delta}$). **Left:** The 3 levels in Blue ($2\times G_{3/2g}$), Green ($2\times G_{3/2g}$) and Red ($1\times E_{5/2g}$). The energy levels without SOC are in Black (dashed): T_{2g} and E_g become degenerate for zero O_h CF. **Right:** Energy shift due to SOC in the CF, $\Delta_{SOC}\Delta E$, between the three limits, 2.5$\boldsymbol{\lambda}$ for zero O_h CF, $\boldsymbol{\lambda}$ for large CF ($\boldsymbol{\delta}$<0) and 0.5$\boldsymbol{\lambda}$ for large CF ($\boldsymbol{\delta}$>0).

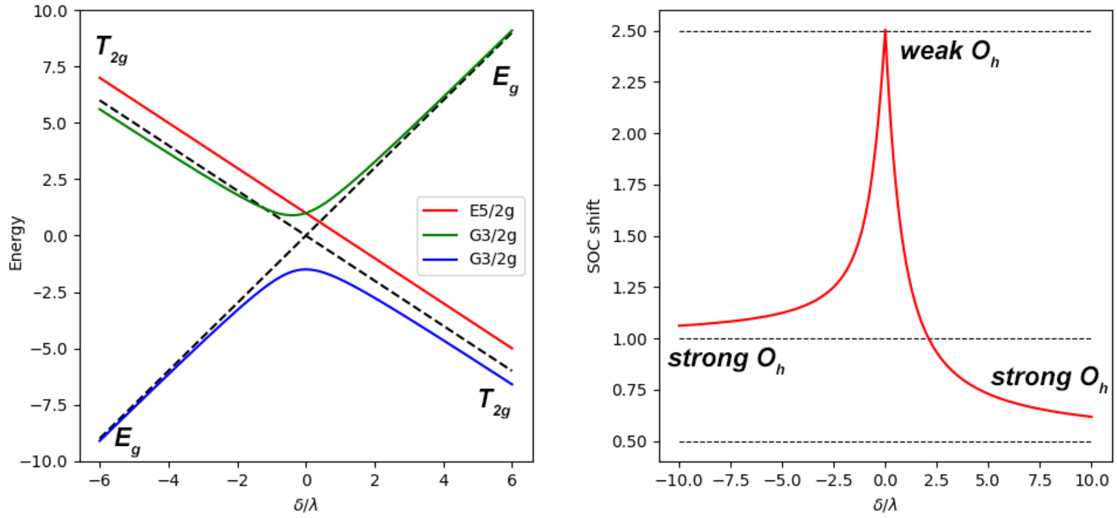

Fig. S35c. Orbital energy levels of an atomic SO split F-shell (SO splitting energy parameter λ, whose value is fixed to 1) in an O_h crystal field (CF splitting energy parameters $\boldsymbol{\delta_1}$ and $\boldsymbol{\delta_2}$). **Left:** The 5 levels, 2 × $G_{3/2u}$ in Blue and Green, 2 × $E_{5/2u}$ in Pink and Yellow, and 1× $E_{1/2u}$ in Red. The energy levels without SOC are in Black (dashed): A_{2u}, T_{1u} and T_{2u} become degenerate for zero O_h CF. **Right:** Energy shift due to SOC in the CF, $\Delta_{SOC}\Delta\boldsymbol{E}$, between the three limits, 3.5$\boldsymbol{\lambda}$ for zero O_h CF, 1.5$\boldsymbol{\lambda}$ for large CF($\boldsymbol{\delta}$>0) and 0.75$\boldsymbol{\lambda}$ for large CF($\boldsymbol{\delta}$<0).

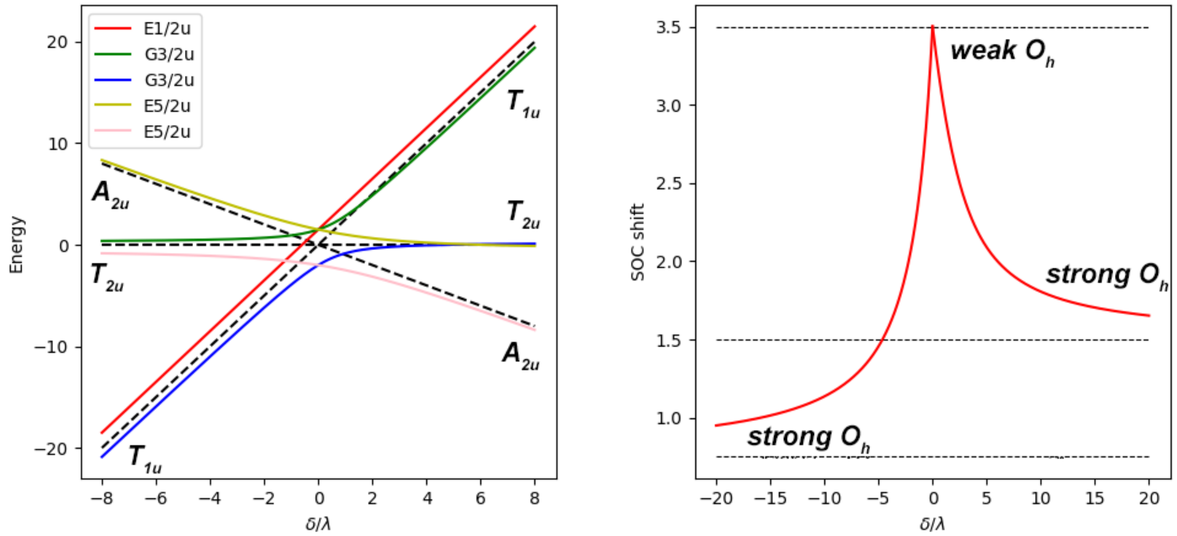

Fig. S35d. Orbital energy levels of an SO split F-shell in an O_h crystal field (SO splitting energy parameter λ; CF splitting energy parameter $\boldsymbol{\delta_1}$ and $\boldsymbol{\delta_2}$). **Left:** The more common "positive CF" case, $\boldsymbol{\lambda = (1+x), \delta_1 = +1(1-x)}$ and $\boldsymbol{\delta_2 = +2½(1-x)}$, for $\boldsymbol{x \in [-1,1]}$, indicating the energy splitting from pure CF to pure SOC (comparable CF and SOC for x = 0) into 5 levels: 2 × $G_{3/2u}$ (Blue, Green), 2 × $E_{3/2u}$ (Pink, Yellow), and ($E_{5/2u}$) and 1 × $E_{1/2}$u (Red). **Right:** The "negative CF" case, $\boldsymbol{\lambda = (1+x), \delta_1 = -1(1-x)}$ and $\boldsymbol{\delta_2 = -2½(1-x)}$ **.**

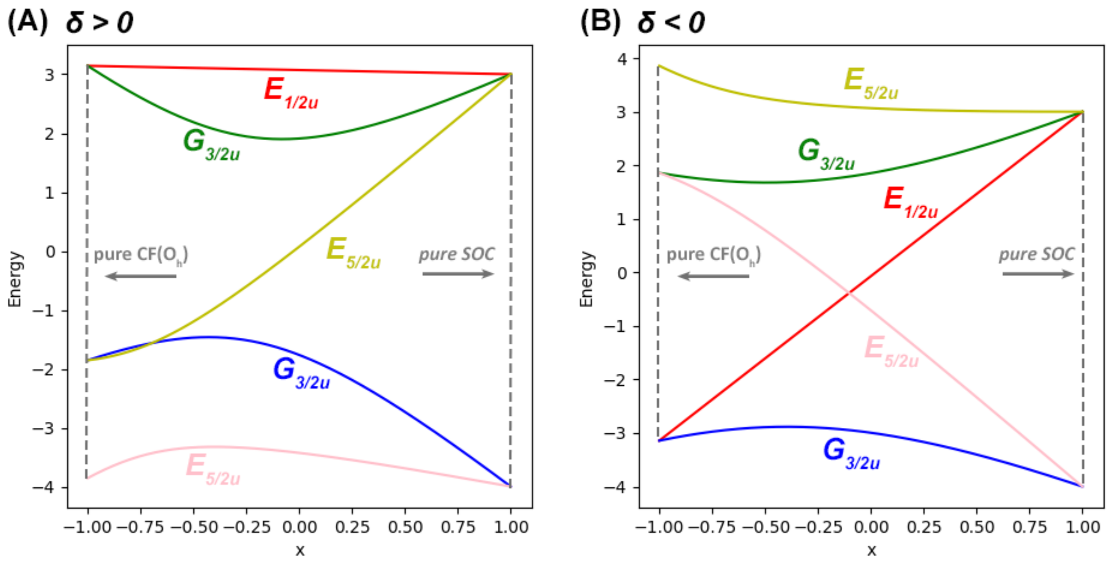

For common positive CF and smaller SOC, the upper T_{1u} f-level is only SO-split by 2¼/3½ ≈ 0.64 of the free-atomic SO-splitting, while the SO splitting of the middle T_{2u} f-level remains negligible.

Coming back to the case of the semi-core U-6p levels, an MO level scheme of *O* O_h $U(OH)_6$ with D(U-O) = 208 pm as in solid δ-[UO_3] was shown in Fig. S36(A). Semi-core U-6p mixes into the O-2p dominated valence orbitals, shifting the O-2p/U-5f $3T_{1u}$ bonding orbitals above the O-2p/U-6d $2E_g$ and O-2p/U-7s $3A_{1g}$ orbitals, which is the well-known effect in molecular U-compounds called "pushing from below" (PFB). The slightly U-5f bonding $4T_{1u}$ orbital of dominant O-2p character is also pushed up due to U-6p admixture. The HOMO is $1T_{1g}$ O-2p, of weakly O-O antibonding type, and corresponding to its symmetry without admixture of U-spdf. The corresponding symmetry characters of the crystal orbitals in δ-[UO_3] are shown in Fig. S36B.

Fig. S36. Molecular and solid-state interactions of U with O. **(A) Left:** Scalar-relativistic orbital level sketch for molecular octahedral $U(OH)_6$ (D(U-O) = 208.1 pm, as in δ-[UO_3]; linear UOH with optimized D(O-H) = 96.8 pm). Scalar ZORA computations at the scalar relativistic Kohn-Sham-PBE level with ADF. Atomic, and O_h molecular, orbital symmetry labels (* meaning anti-bonding), and orbital energies *E* (in eV). The lower-energy atomic-core orbitals up to U-6s/$1A_{1g}$ are omitted. The atomic orbital admixtures in the $U(OH)_6$ molecular orbitals are indicated by colors and dotted lines: O-2s in brown, O-2p in red, U-6p in green, U-5f in blue, U-6d in black, U-7s in purple, U-7p in beige. **(B) Right:** Band structure of δ-[UO_3], from scalar-relativistic KS-PBE calculations with VASP. The corresponding symmetry characters of the crystal orbitals in δ-[UO_3] and the molecular orbitals in $U(O\text{-}H)_6$ are displayed.

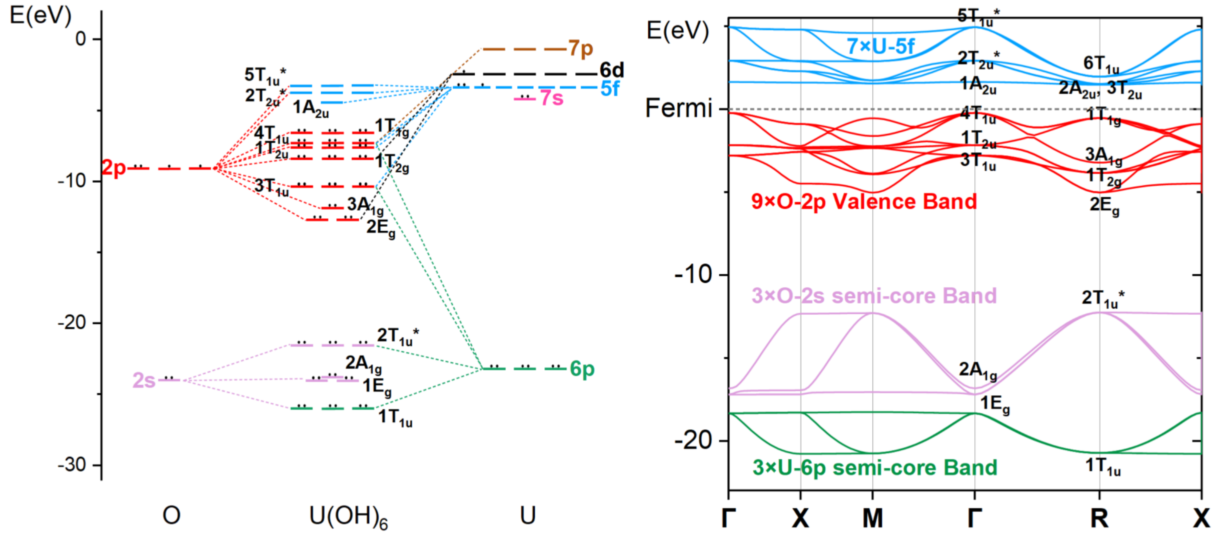

Table S13. Energy levels and splitting $\boldsymbol{\Delta_{SOC}E}$ by SOC (in eV) of AOs for bare U^{3+}, SR- and SOC-ZORA PBE-DF calculations with ADF.

E/eV				$\boldsymbol{\Delta_{SOC}E}$ /eV
SR		SOC		
7P	−15.85	$P_{3/2}$	−15.41	1.64
		$P_{1/2}$	−17.05	
7S	−21.81	$S_{1/2}$	−21.77	
6D	−22.25	$D_{5/2}$	−21.85	0.80
		$D_{3/2}$	−22.65	
5F	−25.64	$F_{7/2}$	−24.94	0.82
		$F_{5/2}$	−25.76	
6P	−45.50	$P_{3/2}$	−43.14	8.65
		$P_{1/2}$	−51.79	
6S	−70.22	$S_{1/2}$	−69.89	

Table S14. Energy levels and splitting $\boldsymbol{\Delta_{SOC}E}$ by SOC (in eV) of AOs for bare U^{6+}, SR- and SOC-ZORA PBE-DF calculations with ADF.

E/eV				$\boldsymbol{\Delta_{SOC}E}$ /eV
SR		SOC		
7P	−40.11	$P_{3/2}$	−39.16	3.46
		$P_{1/2}$	−42.62	
7S	−49.58	$S_{1/2}$	-49.53	
6D	−56.24	$D_{5/2}$	−55.56	1.42
		$D_{3/2}$	−56.98	
5F	−71.62	$F_{7/2}$	−70.81	1.11
		$F_{5/2}$	−71.92	
6P	−86.07	$P_{3/2}$	−83.09	10.92
		$P_{1/2}$	−94.01	
6S	−114.39	$S_{1/2}$	−114.10	

The 7 rather pure U-5f lowest CB orbitals of δ-[UO_3] at the R-point ($6T_{1u}$, $3T_{2u}$ and $2A_{2u}$ at/near CBm) are nearly degenerate without strong CF splitting (but significant SO splitting see Fig. S8). In contrast 7 rather pure U-5f dominated CB orbitals at the Γ-point of δ-[UO_3] ($5T_{1u}$, $2T_{2u}$ and $1A_{2u}$) are similar to the 7 almost UMOs of $U(OH)_6$ in Fig. S36A. The mainly difference between solid δ-[UO_3] and molecular O_h-$U(OH)_6$ is that there are more electronegative O per U in the molecule. In our molecular model there is only one-sided interaction by U on each O atom. Therefore, the pushing up to the VBM is more pronounced in the solid than in the molecule, making $4T_{1u}$ to be the VBM above $1T_{1g}$.

The biggest SO splitting is found in the unperturbed atom without CF (Tables S13-S14). We studied the SO splitting in the O_h molecule at different bond lengths of U-O. Most triply-degenerate MOs keep their coordinative-dative O-2p/U-5f,6p,6d mixture. We present MO level data (SR & SOC) and AO populations in Tables S15-S16. Contraction of the U-O distances

cause more U-6p/O-2p anti-bonding interaction. Remarkably, for larger SO splitting in the occupied orbitals, there occurs smaller SO splitting in the virtual orbitals. The $U(OH)_6$ molecule has U-6p admixture in the occupied orbitals $1T_{1u}$, $2T_{1u}^*$, $3T_{1u}$ and $4T_{1u}$ (Fig. S37) they exhibit larger SO splitting for larger U-6p mixing (see Table S16 and Fig. S38). Shorter U-O distances increase the U-6p admixture into the valence $4T_{1u}$ MO, pushing it up to become the HOMO for the short distance of D(U-O) = 187.3 pm, lying then above $1T_{1g}$. The enhanced PFB by U-6p at short U-O distances suggests that An-oxides under high pressure may give us new insights into the semi-core and valence interactions. Similar results will be shown for the δ-[UO_3] solids under different pressures in Section 7. In summary, high pressure enhances the U-6p participate in valence interactions, causing smaller $\Delta_{SOC}\boldsymbol{E}$ in the lowest $1T_{1u}$ U-6p semi-core crystal orbital, but bigger $\Delta_{SOC}\boldsymbol{E}$ in the higher O-2s semi-core and the various O-2p valence orbitals.

Fig. S37. $U(OH)_6$ molecule with $\boldsymbol{D}_{U\text{-}O}$=208 pm: molecular structures and iso-surfaces for the T_{1g}(HOMO) and 4 important T_{1u} MOs, with iso-value = 0.03 e/bohr^3.

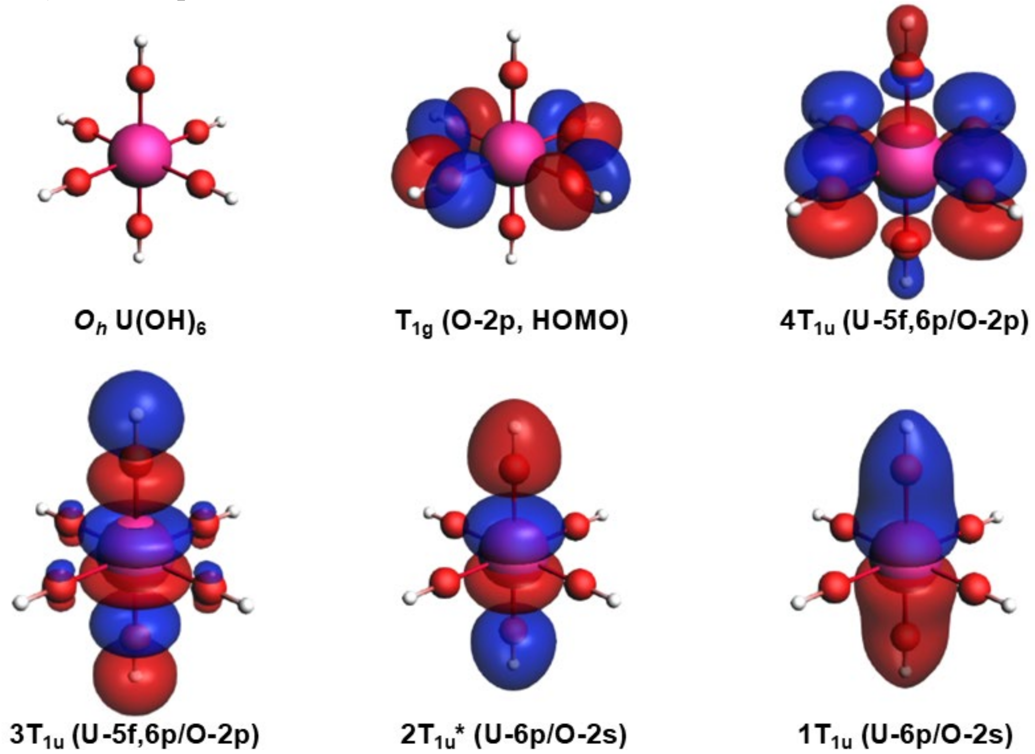

Our $U(OH)_6$ SO splitting for virtual molecular orbitals at the normal bond length of 208pm fit excellently to those experimentally derived results (*95, 96*), both indicating an SO splitting of about 0.2 eV and 0.3 eV for the O_h-CF-split $5T_{1u}^*$ and $2T_{2u}^*$ levels, respectively. There is a no such strong CF splitting in $U(OH)_6$ (U-O = 208pm) with Δ = 1.2 eV, and we further confirm quenching of the f-SO splitting by the CF due to the bond length shortening. We can even see the CF quenching of the SOC directly in the band structure of δ-[UO_3], from Γ to R, in the main text in Fig. 4 right: for the case where the CF becomes weaker (Δ smaller), and the SOC effect becomes stronger. When the CF is too strong, for example for Δ > 3 eV at the Γ point of δ-[UO_3],

Table S15. MO levels of Octahedral $U(O\text{-}H)_6$ molecule with fixed D(U-O) = 187.3, 208.1, 228.9 (in pm) as in δ-[UO_3] (and 10% shorter or longer) and linearly arranged optimized D(O-H) = 97.4, 96.8, 96.8 pm, respectively. Scalar (SR) ZORA computations compared with spin-orbital coupling (SOC) at the Kohn-Sham-PBE level with ADF: Molecular orbital energies E (in eV), Symmetry labels, and bonding characterization.

E/eV						Symmetry		MO type
187.3		208.1		228.9				
SR	SOC	SR	SOC	SR	SOC	SR	SOC	
−0.87	−0.63	−3.32	−2.93	−4.97	−4.53	$5T_{1u}$*	$E_{1/2u}$	U-5f virtual
	−0.68		−3.10		−4.68		$G_{3/2u}$	
−1.33	−1.17	−3.83	−3.63	−5.27	−5.00	$2T_{2u}$*	$E_{5/2u}$	
	−1.34		−3.92		−5.43		$G_{3/2u}$	
−2.44	−2.45	−4.53	−4.63	−5.74	−5.86	$1A_{2u}$	$E_{5/2u}$	
−6.39	−6.38	−6.61	−6.60	−6.80	−6.79	$1T_{1g}$	$G_{3/2g}$	O-2p_π weakly antibonding
	−6.40		−6.62		−6.81		$E_{1/2g}$	
−6.35	−5.71	−7.31	−7.14	−7.41	−7.35	$4T_{1u}$ a)	$G_{3/2u}$	
	−7.42		−7.56		−7.48		$E_{1/2u}$	
-7.88	−7.86	-7.61	-7.60	−7.50	−7.51	$1T_{2u}$	$G_{3/2u}$	O-2p_π weakly bonding
	−7.87		-7.62		−7.51		$E_{5/2u}$	
−9.21	−9.17	−8.46	-8.42	−8.00	−7.98	$1T_{2g}$	$E_{5/2g}$	
	−9.24		-8.48		−8.03		$G_{3/2g}$	
−9.64	−9.42	−10.42	−10.09	−10.98	−10.78	$3T_{1u}$ a)	$G_{3/2u}$	O-2p_σ bonding
	−10.38		−11.12		−11.39		$E_{1/2u}$	
−11.24	−11.23	−11.91	−11.92	-11.99	-12.00	$3A_{1g}$	$E_{1/2g}$	
−13.22	−13.23	−12.70	−12.72	−12.23	−12.24	$2E_g$	$G_{3/2g}$	
−20.17	−19.25	−21.63	−20.45	−22.78	−21.61	$2T_{1u}$*	$G_{3/2u}$	O-2s semi core (anti-bonding with U-6p and non-bonding)
	−22.11		−23.18		−23.59		$E_{1/2u}$	
−23.53	−23.52	−23.80	−23.80	−23.84	−23.84	$2A_{1g}$	$E_{1/2g}$	
−24.11	−24.11	−23.92	−23.92	−23.84	−23.84	$1E_g$	$G_{3/2g}$	
−27.06	−26.36	−26.07	−25.29	−25.69	−24.71	$1T_{1u}$	$G_{3/2u}$	U-6p semi core (bonding with O-2s)
	−30.60		−30.77		−31.43		$E_{1/2u}$	
−47.10	−46.97	−48.65	−48.55	−49.81	−49.69	$1A_{1g}$	$E_{1/2g}$	U-6s core

a) Both $3T_{1u}$ and $4T_{1u}$ are O-2p_σ-2p_π mixed.

Table S16. MO levels and AO population of Octahedral $U(O\text{-}H)_6$ molecule with fixed D(U-O) = 187.3, 208.1, 228.9 (in pm) as in δ-[UO_3] (and 10% shorter or longer) and linearly arranged optimized D(O-H) = 97.4, 96.8, 96.8 pm, respectively. In the middle: Symmetry label and bonding characterization of selected canonical molecular orbitals. Left side: splitting of the molecular orbital energies by spin-orbit coupling, $\Delta_{SOC}E$ in eV. Right side: some AO %ages of the MOs at the scalar relativistic ZORA PBE level, all-electron TZ2P basis sets calculations with ADF.

$\Delta_{SOC}E$/eV			Symmetry	MO type	Admixture for 187.3 pm	Admixture for 208.1 pm	Admixture for 228.9 pm
187.3	208.1	228.9					
			$5T_{1u}$*		1% O-$2p_s$	13% O-$2p_s$	13% O-$2p_s$
0.25	0.49	0.56	$2T_{2u}$*	U-5f virtual	15% O-2p;<br>1% O-3d	17% O-2p	20% O-2p
			$1A_{2u}$		100% U-5f	100% U-5f	100% U-5f
0.02	0.02	0.02	$1T_{1g}$	O-$2p_\pi$ weakly antibonding	99% O-2p	99% O-2p	98% O-2p
1.71	0.42	0.13	$4T_{1u}$ a)		10% U-6p<br>3% U-7p	5% U-5f;<br>1% U-6p<br>3% U-7p	10% U-5f;<br>2% U-7p
0.01	0.02	0.00	$1T_{2u}$	O-$2p_\pi$ weakly bonding	18% U-5f	20% U-5f	24% U-5f
0.07	0.06	0.05	$1T_{2g}$		14% U-6d	14% U-6d	14% U-6d
0.96	1.03	0.61	$3T_{1u}$ a)	O-$2p_\sigma$ bonding	23% U-5f;<br>8% U-6p	15% U-5f;<br>6% U-6p	8% U-5f;<br>6% U-6p
			$3A_{1g}$		0 % U	0 % U	3% U-7s
			$2E_g$		15% U-6d	14% U-6d	12% U-6d
2.86	2.73	1.98	$2T_{1u}$*	O-2s semi core (anti-bonding with U-6p and non-bonding)	34% U-*n*p<br>28% O-2s<br>14% O-2p	40% U-*n*p<br>36% O-2s<br>5% O-2p	30% U-*n*p<br>50% O-2s<br>1% O-2p
			$2A_{1g}$		81% O-2s<br>3% O-2p<br>13% U-ns	80% O-2s<br>2% O-2p<br>11% U-7s	80% O-2s<br>1% O-2p<br>6% U-ns
			$1E_g$		83% O-2s	82% O-2s<br>2% U-6d	80% O-2s<br>2% U-6d
4.24	5.48	6.72	$1T_{1u}$	U-6p semi core (bonding with O-2s)	36% U-*n*p<br>51% O-2s<br>4% O-2p	44% U-*n*p<br>43% O-2s<br>3% O-2p	60% U-*n*p<br>33% O-2s<br>1% O-2p
			$1A_{1g}$	U-6s core	89% U-6s<br>9% O-2sp	96% U-6s<br>1% O-2p	99% U-6s

a) Both $3T_{1u}$ and $4T_{1u}$ are O-$2p_\sigma$-$2p_\pi$ mixed.

Fig. S38. Spin-orbit splitting of the T1u molecular orbitals versus the U-np admixture. Octahedral $U(OH)_6$ with three different U-O distances. Correlation line: $\Delta_{SOC}E$/eV = + 0.1·U-np (in %) R^2=0.95 (without intercept).

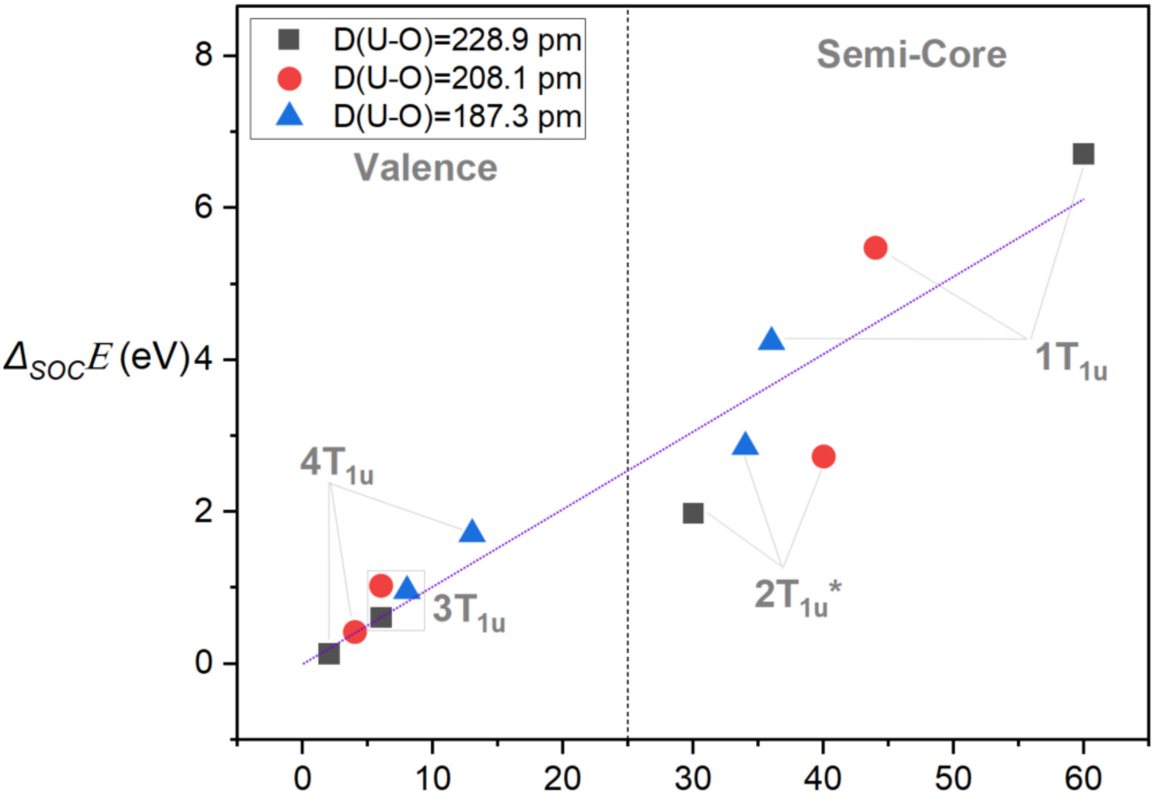

the SO splitting is quenched, and we may neglect the 0.02 to 0.03 eV of SO splitting for all U-5f mixed orbitals. We note that the SO splitting of O-2p is of the same order. In $U(OH)_6$, for occupied T_{1u} molecular orbitals, we found a positive correlation between the SO splitting energy and the U-6p % admixture, shown in Fig. S38.

In Tables S17 and S18, we further confirm that a slight change from O_h structure to a stretched or compressed D_{4h} structure (while keeping the average bond length) will significantly reduce the SOC.

Table S17. MO levels for vertically compressed uranylic D_{4h}, symmetric O_h, and vertically stretched D_{4h} complex-molecule $U(O\text{-}H)_6$, with linearly arranged U-O-H, with optimized D(O-H) for fixed D(U-O). Computations at the scalar and SOC relativistic ZORA Kohn-Sham PBE levels with ADF. Molecular orbital energies E in eV, internuclear distances in pm. As Pyykkö's paper (*97*) shows, optimized R_{axial} and $R_{\text{equatorial}}$ have a nearly linear relationship. Therefore, we can keep the average bond length at 187 pm for the two D_{4h} structures: 4*182 pm + 2*197 pm and 4*192 pm + 2*177 pm.

E/eV						MO symmetry				MO type
Stretched uranyl $U\text{-}O_{ax}$: 2×197 $U\text{-}O_{eq}$: 4×182 Mean:187^{+10}_{-5}		O_h symmetric U-O: 6*187		Compressed uranyl $U\text{-}O_{ax}$: 2*177 $U\text{-}O_{eq}$: 4*192 Mean: 187^{-5}_{+10}		O_h		D_{4h}		
SR	SOC	SR	SOC	SR	SOC	SR	SOC	SR	SOC	
−0.93	−0.72	−0.87	−0.63	−0.79	−0.56	$5T_{1u}$*	$E_{1/2u}$	E_u*	$E_{1/2u}$	U-5f virtual
	−0.71		−0.68		−0.60		$G_{3/2u}$		$E_{3/2u}$	
−0.78	−0.56			−1.04	−0.81			A_{2u}*	$E_{1/2u}$	
−1.18	−1.13	−1.33	−1.17	−1.46	−1.42	$2T_{2u}$*	$E_{5/2u}$	B_{2u}*	$E_{3/2u}$	
−1.38	−1.28		−1.34	−1.23	−1.13		$G_{3/2u}$	E_u*	$E_{3/2u}$	
	−1.40				−1.26				$E_{1/2u}$	
−2.43	−2.44	−2.44	−2.45	−2.43	−2.44	$1A_{2u}$	$E_{5/2u}$	B_{1u}	$E_{3/2u}$	
−6.77	−7.07	−6.35	−5.71	−5.92	−5.46	$4T_{1u}$ a)	$G_{3/2u}$	A_{2u}	$E_{1/2u}$	O-2p_π weakly antibonding
−6.05	−5.47			−6.46	−5.88			E_u	$E_{3/2u}$	
	−5.84		−7.42		−7.15		$E_{1/2u}$		$E_{1/2u}$	
−6.08	−6.07	−6.39	−6.38	−6.43	−6.43	$1T_{1g}$	$G_{3/2g}$	E_g	$E_{3/2g}$	
	−6.09				−6.44				$E_{1/2g}$	
−6.77	−6.77		−6.40	−6.03	−6.04		$E_{1/2g}$	A_{2g}	$E_{1/2g}$	
−7.58	−7.44	−7.88	−7.86	−8.04	−7.93	$1T_{2u}$	$G_{3/2u}$	E_u	$E_{3/2u}$	O-2p_π weakly bonding
	−8.01				−8.23				$E_{1/2u}$	
−8.40	−8.38		−7.87	−7.40	−7.39		$E_{5/2u}$	B_{2u}	$E_{3/2u}$	
−9.14	−9.11	−9.21	−9.17	−9.81	−9.77	$1T_{2g}$	$E_{5/2g}$	E_g	$E_{3/2g}$	
	−9.16		−9.24		−9.83		$G_{3/2g}$		$E_{1/2g}$	
−9.88	−9.88			−8.59	−8.60			B_{2g}	$E_{3/2g}$	
−9.70	−9.57	−9.64	−9.42	−9.46	−9.50	$3T_{1u}$ a)	$G_{3/2u}$	A_{2u}	$E_{1/2u}$	O-2p_σ bonding
−9.78	−9.60			−9.96	−9.83			E_u	$E_{3/2u}$	
	−10.38		−10.38		−10.39		$E_{1/2u}$		$E_{1/2u}$	
−11.07	−11.07	−11.24	−11.23	−11.13	−11.12	$3A_{1g}$	$E_{1/2g}$	A_{1g}	$E_{1/2g}$	
−12.84	−12.84	−13.22	−13.23	−13.92	−13.91	$2E_g$	$G_{3/2g}$	A_{1g}	$E_{1/2g}$	
−13.79	−13.79			−12.66	−12.67			B_{1g}	$E_{3/2g}$	
−20.04	−19.16	−20.17	−19.25	−20.48	−19.53	$2T_{1u}$*	$G_{3/2u}$	A_{2u}*	$E_{1/2u}$	O-2s semi core (anti-bonding with U-6p and non-bonding)
−20.30	−19.46			−20.10	−19.12			E_u*	$E_{3/2u}$	
	−22.09		−22.11		−22.10		$E_{1/2u}$		$E_{1/2u}$	
−23.06	−23.07	−23.53	−23.52	−23.30	−23.30	$2A_{1g}$	$E_{1/2g}$	A_{1g}	$E_{1/2g}$	
−24.11	−24.11	−24.11	−24.11	−24.88	−24.87	$1E_g$	$G_{3/2g}$	A_{1g}	$E_{1/2g}$	
−24.63	−24.63			−23.62	−23.63			B_{1g}	$E_{3/2g}$	
−25.58	−25.51	−27.06	−26.36	−28.89	−31.02	$1T_{1u}$	$U_{3/2u}$	A_{2u}	$E_{1/2u}$	U-6p semi-core (bonding with O-2s)
−27.92	−27.20			−26.28	−25.59			E_u	$E_{3/2u}$	
	−30.86		−30.60		−26.98		$E_{1/2u}$		$E_{1/2u}$	
−47.16	−47.03	−47.10	−46.97	−47.17	−47.04	$1A_{1g}$	$E_{1/2g}$	A_{1g}	$E_{1/2g}$	U-6s core; non-bonding

a) Both $3T_{1u}$ and $4T_{1u}$ are O-2p_σ-2p_π mixed.

Table S18. The energy level splitting $\Delta_{SOC}E$ of MO by SOC (in eV) for Vertically compressed uranylic D_{4h}, symmetric O_h, and vertically stretched D_{4h} complex-molecule U(O-H)$_6$ with linearly arranged U-O-H, with optimized D(O-H) and fixed D(U-O).

$\Delta_{SOC}E$/eV: Stretched Uranyl U-O$_{ax}$: 2×197 U-O$_{eq}$: 4×182 Mean:187^{+10}_{-5}	$\Delta_{SOC}E$/eV: O_h symmetric U-O: 6*187	$\Delta_{SOC}E$/eV: Compressed uranyl U-O$_{ax}$: 2*177 U-O$_{eq}$: 4*192 Mean:187^{+5}_{-10}	Sym-metry	MO type
0.23	0.25	0.24	$5T_{1u}$*<br>$2T_{2u}$*<br>$1A_{2u}$	U-5f virtual
0.88	1.71	1.15	$4T_{1u}$ a)	O-2p_π weakly antibonding
0.73	0.96	0.39	$3T_{1u}$ a)	O-2p_σ bonding
2.67	2.86	2.60	$2T_{1u}$*	O-2s semi core (anti-bonding with U-6p and non-bonding)
3.01	4.24	2.82	$1T_{1u}$	U-6p semi core (bonding with O-2s)

a) Both $3T_{1u}$ and $4T_{1u}$ are O-2p_σ-2p_π mixed.

Section 7. SOC in High-Pressure Phases

The phase transitions and electronic structures of actinide solids under high pressure are an important and active research field. [UO_3] under high pressure has been discussed in detail (*12, 13*). the α-phase is the most stable among the common ones at all pressures. However, there are three new phases predicted to be more stable under very high pressure, with space groups $P6_3mmc$, $Fm\bar{3}m$ and Pm3n, here referred to as x-[UO_3], y-[UO_3] and z-[UO_3] respectively.

For these $5f^0$-actinide oxides (and for most other materials), the general rule is that the band gap decreases for high pressure, eventually becoming metallic conductors. For the α- and δ-[UO_3] and [ThO_2] phases, the geometric structures and coordination numbers of An do not change under pressure. We see that an increasing pressure increases the PFB with increasing U-6p mixing into the O-2p band at the VBM, thereby enhancing the SO splitting of the valence band (see Table S19). For example, α-[UO_3] at 0 GPa, we see in the projected band Fig. S13 (left) that some bands with some more U-6p admixture (green color) still lie under the VBM level. Although these bands have important SO splitting, it still does not have an influence on the VBM level or the size of the band gap. However, at higher pressures, the increasing U-6p mixing pushes those bands up close to or even beyond the original VBM (Figs. S14-S15 left). Thereby the significant SO splitting originating in the U-6p admixture changes the energy values of the VBM and the band gap.

Remarkably, for the VBM of the β-, γ- and η-[UO_3] phases, the conclusion is the opposite (see Table S19). Although higher pressure in deed pushes more U-6p admixture into the VBM orbital, the SO splitting becomes smaller! Apparently, this is because there is a very big structure change and change of coordination number of U under pressure; CN increases form 6 or 7 to 8-12. The stronger and lower symmetric CF at higher pressure quenches the SOC at the VBM.

Table S19. Orbital energy level changes by SOC, $\Delta_{SOC}E$/eV, at CBm and VBM, for different phases of [UO_3], and for [ThO_2], the An-np and An-5f % contribution at VBM, the SO change of the gap, $\Delta_{SOC}\Delta E_{gap}$, and the SOC-suppressed SR gap, $\Delta E_{gap}(SR)$, all energies in eV. Calculations with VASP at the SR-ZORA PBE approximation. All at 3 different pressures: 0, 40, 80 GPa for the common phases α-, β-, γ-, δ-, η-[UO_3] and [ThO_2]; 80, 160, and 240 GPa for the predicted (*12*) high-pressure phases x-[UO_3], y-[UO_3]. We have studied the electronic structures of the common (meta-)stable phases α-, β-, γ-, δ-, η-[UO_3] and [ThO_2] under 0, 40 and 80 GPa pressure at the PBE density functional approximation, with results in Table S18 for SOC energy shifts of the valence band maximum (VBM), the conduction band minimum (CBm), the band gap, and the U-np and U-5f atomic orbital admixtures at the O-2p VBM.

Phase	Space Group	Pressure / GPa	$\Delta_{SOC}E$ (CBm) / eV	$\Delta_{SOC}E$ (VBM) / eV	An-np in VBM	An-5f in VBM	$\Delta_{SOC}\Delta E_{gap}$ / eV	$\Delta E_{gap}(SR)$ / eV
α-[UO_3]	P$\bar{3}$m1	0	−0.30	+0.07	3%	1%	−0.37	1.68
		40	−0.29	+0.18	5%	2%	−0.47	2.04
		80	−0.23	+0.36	14%	22%	−0.59	1.94
β-[UO_3]	P2_1	0	−0.26	+0.20	7%	13%	−0.46	1.44
		40	−0.31	+0.06	2%	11%	−0.37	1.32
		80	−0.06	+0.06	3%	13%	−0.12	0.72
γ-[UO_3]	I4_1	0	−0.24	+0.19	6%	14%	−0.43	1.89
		40	−0.18	+0.14	7%	30%	−0.32	1.38
		80	−0.12	+0.15	6%	23%	−0.27	1.11
δ-[UO_3]	Pm$\bar{3}$m	0	−0.42	+0.50	12%	12%	−0.92	1.67
		40	−0.35	+0.65	15%	21%	−1.00	1.47
		80	−0.26	+0.72	17%	27%	−0.98	1.11
η-[UO_3]	P$2_12_12_1$	0	−0.31	+0.22	5%	10%	−0.53	1.91
		40	−0.15	+0.12	5%	2%	−0.27	0.92
		80	−0.12	−0.02	9%	34%	−0.10	0.66
x-[UO_3]	P63mmc	80	−0.11	−0.03	9%	35%	−0.08	0.66
		160	−0.07	−0.04	10%	36%	−0.03	0.39
		240	−0.05	−0.06	11%	38%	+0.01	0.14
y-[UO_3] [a)]	Fm$\bar{3}$m	80	+0.04	+0.29	6%	3%	−0.25	0.75
		160	+0.05	+0.35	7%	3%	−0.30	0.51
		240	+0.03	+0.42	8%	3%	−0.39	0.25
[ThO_2]	Fm$\bar{3}$m	0	−0.05	+0.05	5%	6%	−0.10	4.45
		40	−0.03	+0.09	9%	5%	−0.12	4.56
		80	−0.02	+0.15	11%	7%	−0.17	4.50

a) Only data for Fm3m-[UO_3] is calculated by HSE. Because under PBE it is a metal, while HSE give us a band gap.

For all these solids, the strong CF at the high pressure destroys the high degeneracy of the atomic 5f atomic orbitals more and more, dominating the conduction band. Therefore, the SO splitting at the CBm is reduced. The high-pressure-stable y-[UO_3] phase has a very special structure: there are 6 equivalent next-nearest O atoms around the U atom, similar to δ-[UO_3]. Therefore triply-degenerate orbitals are found at the VBM, at the Γ point (Figs. S31-S33). Similar to the SO splitting results of molecular U(OH)$_6$, we found a positive correlation for δ-[UO_3] between the SO splitting energy and the U-6p % admixture to the various crystal orbitals of T_{1u} symmetry shown in Tables S20-S21 and Fig. S39. In conclusion, for high pressure phases, it is even more important not to neglect SOC, because high pressure may enhance the spin-orbit coupling at the VBM, leading to easily observable effects.

Table S20. Crystal orbitals energy levels of PBE-SR and PBE-SOC in VASP, for δ-[UO_3], under 0, 40, and 80 GPa, with D(U-O) = 189.2, 196.3, 208.1 (in pm) The energy zero point is Fermi energy of 0 GPa.

E/eV						Symmetry		CO type
80 GPa		40 GPa		0 GPa				
189.2		196.3		208.1				
SR	SOC	SR	SOC	SR	SOC	SR	SOC	
6.46	6.71	4.55	4.88	1.96	2.36	$6T_{1u}$	$E_{1/2u}$	U-5f virtual
	6.67		4.77		2.21		$G_{3/2u}$	
5.61	5.89	3.90	4.26	1.52	1.94	$3T_{2u}$	$E_{5/2u}$	
	5.56		3.78		1.30		$G_{3/2u}$	
5.33	5.06	3.75	3.40	1.47	1.05	$2A_{2u}$	$E_{5/2u}$	
11.40	11.02	8.61	8.41	4.94	4.95	$5T_{1u}$*	$E_{1/2u}$	
	11.86		8.96		5.16		$G_{3/2u}$	
7.86	7.92	5.76	5.83	2.92	3.02	$2T_{2u}$*	$E_{5/2u}$	
	7.99		5.87		3.00		$G_{3/2u}$	
5.91	5.89	4.11	4.07	1.64	1.57	$1A_{2u}$	$E_{5/2u}$	
1.68	1.67	0.78	0.77	−0.54	−0.54	$1T_{1g}$	$G_{3/2g}$	O-2p_π weakly antibonding
	1.66		0.75		−0.56		$E_{1/2g}$	
4.19	4.91	2.25	2.90	−0.23	0.28	$4T_{1u}$ a)	$G_{3/2u}$	
	2.53		0.82		−1.28		$E_{1/2u}$	
−0.56	−0.56	−1.21	−1.20	−2.16	−2.15	$1T_{2u}$	$G_{3/2u}$	O-2p_π weakly bonding
	−0.58		−1.22		−2.18		$E_{5/2u}$	
−3.03	−2.99	−3.36	−3.32	−3.85	−3.81	$1T_{2g}$	$E_{5/2g}$	
	−3.06		−3.39		−3.88		$G_{3/2g}$	
−1.29	−1.31	−1.89	−1.91	−2.80	−2.82	$3T_{1u}$ a)	$G_{3/2u}$	O-2p_σ bonding
	−1.26		−1.87		−2.80		$E_{1/2u}$	
0.15	0.15	−1.36	−1.37	−3.22	−3.23	$3A_{1g}$	$E_{1/2g}$	
−3.76	−3.80	−4.31	−4.35	−5.02	−5.05	$2E_g$	$G_{3/2g}$	
−6.75	−4.96	−9.07	−7.33	−12.25	−10.65	$2T_{1u}$*	$G_{3/2u}$	O-2s semi core (U-6p anti-bonding & non-bonding)
	−10.30		−12.29		−14.79		$E_{1/2u}$	
−14.47	−14.47	−15.53	−15.54	−16.83	−16.84	$2A_{1g}$	$E_{1/2g}$	
−15.88	−14.45	−16.42	−15.19	−17.20	−16.39	$1E_g$	$G_{3/2g}$	
−19.92	−19.22	−20.22	−19.48	−20.72	−19.86	$1T_{1u}$	$G_{3/2u}$	U-6p semi core (O-2s bonding)
	−22.55		−23.17		−24.30		$E_{1/2u}$	
−38.89	−38.84	−40.42	−40.37	−42.64	−42.59	$1A_{1g}$	$E_{1/2g}$	U-6s core

a) Both $3T_{1u}$ and $4T_{1u}$ are O-2p_σ-2p_π mixed.

Table S21. CO levels and AO populations of δ-[UO_3] with U at O_h symmetry position, under 0, 40, and 80 GPa pressure, yielding D(U-O) = 208.1 pm, 196.3 pm, and 189.2 pm, respectively. In the Middle: symmetry label and bonding type of the crystal orbital. Left side: splitting of the crystal orbital energies by spin-orbit coupling, $\Delta_{SOC}E$ in eV. Right side: dominant AO %-ages of the COs at the scalar relativistic DF-PBE level calculations with VASP.

$\Delta_{SOC}E$/eV			Sym-metry	CO type	AO Admixtures in the COs		
80GPa	40GPa	0 GPa			D(U-O): 189.2pm<br>at 80 GPa	D(U-O): 196.3pm<br>at 40 GPa	D(U-O): 208.1pm<br>at 0 GPa
189.2	196.3	208.1					
0.52	0.63	0.82	$6T_{1u}$ [c)]	U-5f<br>**virtual, conduction**	**89%U-5f**<br>2% O-2s	**92% U-5f**<br>2% O-2s	**95% U-5f**<br>1% O-2s
			$3T_{2u}$ [c)]		**100% U-5f**	**100% U-5f**	**100% U-5f**
			$2A_{2u}$ [c)]		**93% U-5f**	**96% U-5f**	**98% U-5f**
0.48	0.39	0.29	$5T_{1u}$* [b)]		**52%U-5f, 40%O-2p**<br>**5% U-6p**, 2% U-7p	**55%U-5f, 39%O-2p**<br>**3% U-6p**, 2% U-7p	**59%U-5f, 38% O-2p**<br>**2% U-6p**, 1% U-7p
			$2T_{2u}$* [b)]		**81%U-5f, 19%O-2p**	**80%U-5f, 20%O-2p**	**78%U-5f, 22% O-2p**
			$1A_{2u}$ [b)]		**100% U-5f**	**100% U-5f**	**100% U-5f**
0.02	0.02	0.02	$1T_{1g}$ [c)]	$O-2p_{\pi}$ **valence** non-&weakly anti- bonding with PFB	**100% O-2p**	**100% O-2p**	**100% O-2p**
2.38	2.08	1.56	$4T_{1u}$ [a,b)]		**59%O-2p, 22%U-5f**<br>**11% U-6p**, 8% U-7p	**66%O-2p, 18% U-5f**<br>**9% U-6p**, 8% U-7p	**78%O-2p, 11%U-5f**<br>**5% U-6p**, 6% U-7p
0.01	0.02	0.03	$1T_{2u}$ [b)]	$O-2p_{\pi}$ **valence** weakly U-f,d bonding	**81% O-2p**<br>**19% U-5f**	**80% O-2p**<br>**20% U-5f**	**78% O-2p**<br>**22% U-5f**
0.08	0.08	0.07	$1T_{2g}$ [c)]		**75% O-2p**<br>**25% U-6d**	**75% O-2p**<br>**25% U-6d**	**75% O-2p**<br>**25% U-6d**
0.05	0.05	0.02	$3T_{1u}$ [a,b)]	$O-2p_{\sigma}$ **valence** U-f,d,s bonding (f: &PFB)	**72% O-2p, 25%U-5f**<br>**2% U-6p**, 2% U-7p	**70%O-2p, 26%U-5f**<br>**2% U-6p**, 2% U-7p	**67%O-2p, 30%U-5f**<br>**1% U-6p**, 1% U-7p
			$3A_{1g}$ [c)]		**39%O-2p, 62%U-7s**	**51%O-2p, 50%U-7s**	**65%O-2p, 35%U-7s**
			$2E_g$ [c)]		**79%O-2p, 21%U-6d**	**77%O-2p, 23%U-6d**	**75%O-2p, 25%U-6d**
5.34	4.95	4.14	$2T_{1u}$* [c)]	O-2s<br>**semi-core**<br>(U-6p anti-& non-bonding)	**36%O-2s, 46%U-6p**<br>13% U-7p, 5% U-5f	**40%O-2s, 45%U-6p**<br>11% U-7p, 4% U-5f	**48%U-2s, 41%O-6p**<br>10% U-7p, 2% U-5f
			$2A_{1g}$ [b)]		90%O-2s, 6%U-6s<br>5% U-7s	91%O-2s, 4%U-6s<br>5% U-7s	93%O-2s, 2%U-6s<br>6% U-7s
			$1E_g$ [b)]		82%O-2s, 18%U-6d	83%O-2s,17%U-6d	85%O-2s, 15%U-6d
3.32	3.70	4.44	$1T_{1u}$ [c)]	U-6p **semi- core** (O-2s bonding)	**46%U-6p, 54%O-2s**<br>1% U-5f	**49%U-6p, 51%O-2s**<br>1% U-5f	**55%U-6p, 46%O-2s**<br>1% U-5f
			$1A_{1g}$ [b)]	U-6s<br>**core**	**94% U-6s**<br>2% O-2s	**96% U-6s**<br>1% O-2s	**98% U-6s**<br>2% O-2s

a) Both $3T_{1u}$ and $4T_{1u}$ are $O-2p_{\sigma}-2p_{\pi}$ mixed.

b) These COs are at Γ point , see Fig. S36

c) These COs are at R point , see Fig. S36

Fig. S39. Spin-orbit splitting of the T_{1u} crystal orbitals versus the U-*n*p admixture. Solid δ-[UO3] at three different pressures. Correlation line: $\Delta_{SOC}E$/eV = + 0.09·U-*n*p (in %), R^2=0.98 (without intercept)

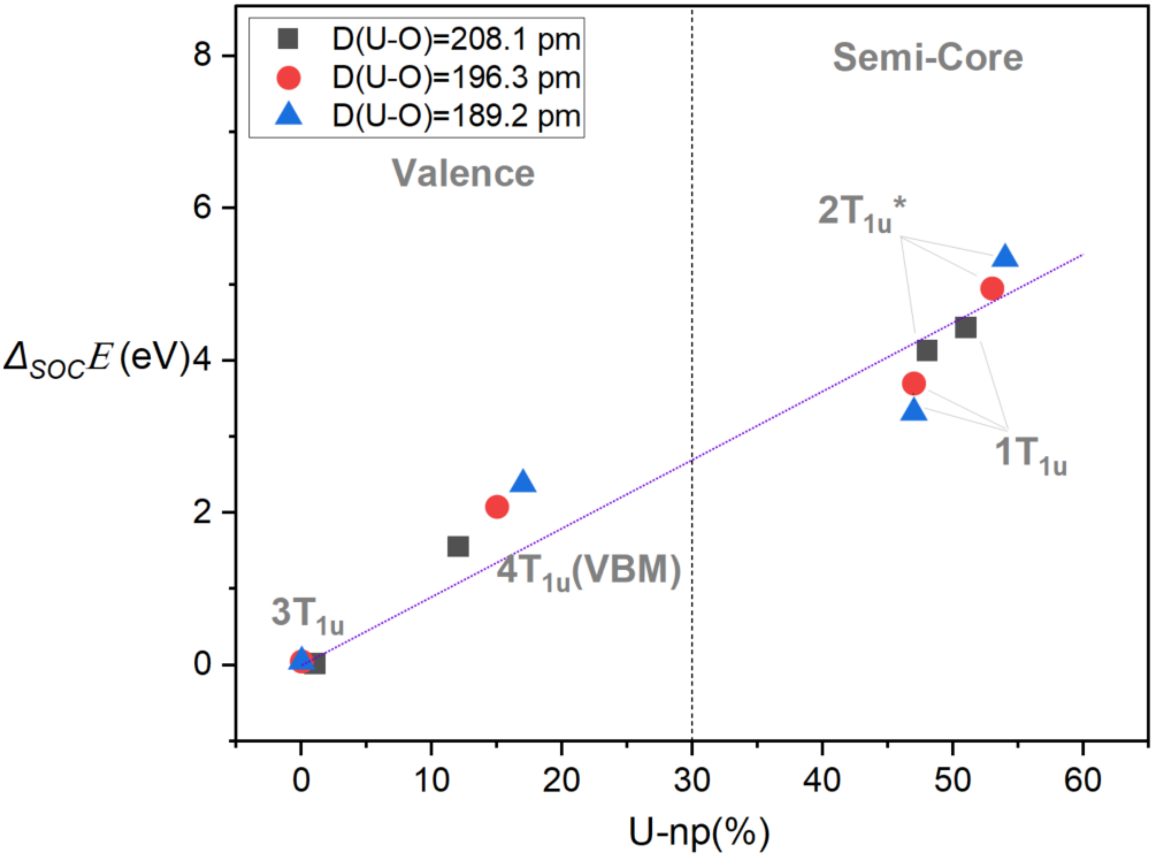

SI References

1. A. J. Cohen, P. Mori-Sánchez, W. Yang, Fractional charge perspective on the band gap in density-functional theory. *Phys. Rev. B* **77**, 115123 (2008).
2. A. J. Cohen, P. Mori-Sánchez, W. Yang, Insights into Current Limitations of Density Functional Theory. *Science* **321**, 792-794 (2008).
3. H. Jiang, M.-Y. Zhang, Density-functional theory methods for electronic band structure properties of materials. *Sci. Sin. Chim.* **50**, 1344-1362 (2020).
4. J. P. Perdew, M. Levy, Physical Content of the Exact Kohn-Sham Orbital Energies: Band Gaps and Derivative Discontinuities. *Phys. Rev. Lett.* **51**, 1884-1887 (1983).
5. S. L. Dudarev, D. N. Manh, A. P. Sutton, Effect of Mott-Hubbard correlations on the electronic structure and structural stability of uranium dioxide. *Philos. Mag. B* **75**, 613-628 (1997).
6. J. Schoenes, Optical properties and electronic structure of UO_2. *J. Appl. Phys.* **49**, 1463-1465 (1978).
7. T. T. Meek, B. von Roedern, P. G. Clem, R. J. Hanrahan, Some optical properties of intrinsic and doped UO_2 thin films. *Mater. Lett.* **59**, 1085-1088 (2005).
8. S. L. Dudarev, G. A. Botton, S. Y. Savrasov, C. J. Humphreys, A. P. Sutton, Electron-energy-loss spectra and the structural stability of nickel oxide: An LSDA+U study. *Phys. Rev. B* **57**, 1505-1509 (1998).
9. S. L. Dudarev *et al.*, Understanding STM images and EELS spectra of oxides with strongly correlated electrons: a comparison of nickel and uranium oxides. *Micron* **31**, 363-372 (2000).
10. T. Yamazaki, A. Kotani, Systematic Analysis of 4f Core Photoemission Spectra in Actinide Oxides. *J. Phys. Soc. Jpn.* **60**, 49-52 (1991).
11. A. E. Shields *et al.*, Pressure-induced evolution of the lattice dynamics for selected UO_3 polymorphs. *J. Nucl. Mater.* **584**, 154577 (2023).
12. J.-J. Ma *et al.*, Pressure-induced structural and electronic phase transitions of uranium trioxide. *Phys. Rev. B* **104**, 174103 (2021).
13. N. A. Brincat, S. C. Parker, M. Molinari, G. C. Allen, M. T. Storr, Ab Initio Investigation of the UO_3 Polymorphs: Structural Properties and Thermodynamic Stability. *Inorg. Chem.* **53**, 12253-12264 (2014).
14. X.-D. Wen *et al.*, Screened hybrid and DFT + U studies of the structural, electronic, and optical properties of U_3O_8. *J. Phys.: Condens. Matter* **25**, 025501 (2013).
15. J. I. Ranasinghe, L. Malakkal, E. Jossou, B. Szpunar, J. A. Szpunar, Comprehensive study on the electronic and optical properties of α-U_3O_8. *Comput. Mater. Sci* **171**, 109264 (2020).
16. N. A. Brincat, S. C. Parker, M. Molinari, G. C. Allen, M. T. Storr, Density functional theory investigation of the layered uranium oxides U_3O_8 and U_2O_5. *Dalton Trans.* **44**, 2613-2622 (2015).
17. H. He, D. A. Andersson, D. D. Allred, K. D. Rector, Determination of the Insulation Gap of Uranium Oxides by Spectroscopic Ellipsometry and Density Functional Theory. *J. Phys. Chem. C* **117**, 16540-16551 (2013).
18. H. Y. Geng, H. X. Song, K. Jin, S. K. Xiang, Q. Wu, First-principles study on oxidation effects in uranium oxides and high-pressure high-temperature behavior of point defects in uranium dioxide. *Phys. Rev. B* **84**, 174115 (2011).
19. X. Tian *et al.*, First principles calculation of UO_2 polymorphs and phase transitions under compressive and tensile loading. *Comput. Mater. Sci* **169**, 109124 (2019).
20. S. L. Dudarev *et al.*, Parametrization of LSDA+U for noncollinear magnetic configurations: Multipolar magnetism in UO_2. *Phys. Rev. Mater.* **3**, 083802 (2019).
21. B. Dorado, G. Jomard, M. Freyss, M. Bertolus, Stability of oxygen point defects in UO_2 by first-principles DFT+U calculations: Occupation matrix control and Jahn-Teller distortion. *Phys. Rev. B* **82**, 035114 (2010).
22. B. Dorado *et al.*, Advances in first-principles modelling of point defects in UO2: f electron correlations and the issue of local energy minima. *J. Phys.: Condens. Matter* **25**, 333201 (2013).
23. B. Dorado, B. Amadon, M. Freyss, M. Bertolus, DFT+U calculations of the ground state and metastable states of uranium dioxide. *Phys. Rev. B* **79**, 235125 (2009).
24. A. J. Devey, First principles calculation of the elastic constants and phonon modes of UO_2 using GGA+U with orbital occupancy control. *J. Nucl. Mater.* **412**, 301-307 (2011).

25. P. Zhang, B.-T. Wang, X.-G. Zhao, Ground-state properties and high-pressure behavior of plutonium dioxide: Density functional theory calculations. *Phys. Rev. B* **82**, 144110 (2010).
26. B.-T. Wang, H. Shi, W. Li, P. Zhang, First-principles LDA+U and GGA+U study of neptunium dioxide. *Phys. Rev. B* **81**, 045119 (2010).
27. S. Singh *et al.*, Ab-initio calculation on electronic and optical properties of ThO_2, UO_2 and PuO_2. *J. Nucl. Mater.* **511**, 128-133 (2018).
28. L. Petit, A. Svane, Z. Szotek, W. M. Temmerman, G. M. Stocks, Electronic structure and ionicity of actinide oxides from first principles. *Phys. Rev. B* **81**, 045108 (2010).
29. J. T. Pegg *et al.*, Magnetic structure of UO_2 and NpO_2 by first-principle methods. *Phys. Chem. Chem. Phys.* **21**, 760-771 (2019).
30. J. T. Pegg *et al.*, Hidden magnetic order in plutonium dioxide nuclear fuel. *Phys. Chem. Chem. Phys.* **20**, 20943-20951 (2018).
31. J. T. Pegg, X. Aparicio-Anglès, M. Storr, N. H. de Leeuw, DFT+U study of the structures and properties of the actinide dioxides. *J. Nucl. Mater.* **492**, 269-278 (2017).
32. H. Nakamura, M. Machida, M. Kato, Effects of spin-orbit coupling and strong correlation on the paramagnetic insulating state in plutonium dioxides. *Phys. Rev. B* **82**, 155131 (2010).
33. J.-B. Morée, R. Outerovitch, B. Amadon, First-principles calculation of the Coulomb interaction parameters U and J for actinide dioxides. *Phys. Rev. B* **103**, 045113 (2021).
34. X. Y. Liu, D. A. Andersson, B. P. Uberuaga, First-principles DFT modeling of nuclear fuel materials. *J. Mater. Sci.* **47**, 7367-7384 (2012).
35. M. Idiri, T. Le Bihan, S. Heathman, J. Rebizant, Behavior of actinide dioxides under pressure: UO_2 and ThO_2. *Phys. Rev. B* **70**, 014113 (2004).
36. A. Bouasria *et al.*, Ground state properties of actinide dioxides: A self-consistent Hubbard U approach with spin orbit coupling. *Int. J. Comput. Mater. Sci. Eng.* **06**, 1750006 (2017).
37. J. T. Pegg, A. E. Shields, M. T. Storr, D. O. Scanlon, N. H. de Leeuw, Interaction of hydrogen with actinide dioxide (011) surfaces. *J. Chem. Phys.* **153**, (2020).
38. V. Kanchana *et al.*, High-pressure study of binary thorium compounds from first principles theory and comparisons with experiment. *Acta Cryst. B* **70**, 459-468 (2014).
39. K. E. Garrett *et al.*, First principles investigation of the structural and bonding properties of hydrated actinide (IV) oxalates, $An(C_2O_4)_2 \cdot 6H_2O$ (An = U, Pu). *Comput. Mater. Sci* **153**, 146-152 (2018).
40. H. Gao, M. Li, Y. Yang, P. Zhang, First-principles calculation of structural, magnetic and electronic properties of $PuO_{2-x}H_x$, $0 \leq x \leq 2$. *J. Alloys Compd.* **857**, 157592 (2021).
41. J.-L. Chen, N. Kaltsoyannis, DFT + U study of $U_{1-y}An_yO_{2-x}$ (An = Np, Pu, Am and Cm) {111}, {110} and {100} surfaces. *Appl. Surf. Sci.* **537**, 147972 (2021).
42. J.-L. Chen, N. Kaltsoyannis, Computational Study of the Bulk and Surface Properties of Minor Actinide Dioxides $MAnO_2$ (MAn = Np, Am, and Cm); Water Adsorption on Stoichiometric and Reduced {111}, {110}, and {100} Surfaces. *J. Phys. Chem. C* **123**, 15540-15550 (2019).
43. B. Ao, R. Qiu, H. Lu, P. Chen, Differences in the Existence States of Hydrogen in UO_2 and PuO_2 from DFT + U Calculations. *J. Phys. Chem. C* **120**, 18445-18451 (2016).
44. J.-L. Chen, N. Kaltsoyannis, DFT + U Study of Uranium Dioxide and Plutonium Dioxide with Occupation Matrix Control. *J. Phys. Chem. C* **126**, 11426-11435 (2022).
45. H. Wang, K. Konashi, LDA+U study of Pu and PuO_2 on ground state with spin–orbital coupling. *J. Alloys Compd.* **533**, 53-57 (2012).
46. J. P. Allen, G. W. Watson, Occupation matrix control of d- and f-electron localisations using DFT + U. *Phys. Chem. Chem. Phys.* **16**, 21016-21031 (2014).
47. H. Xiao, J. Tahir-Kheli, W. A. Goddard, III, Accurate Band Gaps for Semiconductors from Density Functional Theory. *J. Phys. Chem. Let.* **2**, 212-217 (2011).
48. X.-D. Wen *et al.*, Effect of spin-orbit coupling on the actinide dioxides AnO_2 (An=Th, Pa, U, Np, Pu, and Am): A screened hybrid density functional study. *J. Chem. Phys.* **137**, 154707 (2012).
49. P. R. C. Kent, G. Kotliar, Toward a predictive theory of correlated materials. *Science* **361**, 348-354 (2018).

50. H. R. Hoekstra, S. Siegel, F. X. Gallagher, The uranium-oxygen system at high pressure. *J. Inorg. Nucl. Chem.* **32**, 3237-3248 (1970).

51. S. Siegel, H. Hoekstra, E. Sherry, The crystal structure of high-pressure UO_3. *Acta Cryst.* **20**, 292-295 (1966).

52. C. J. Pickard *et al.*, Structural Properties of Lanthanide and Actinide Compounds within the Plane Wave Pseudopotential Approach. *Phys. Rev. Lett.* **85**, 5122-5125 (2000).

53. G. Kresse, J. Hafner, Ab initio molecular-dynamics simulation of the liquid-metal--amorphous-semiconductor transition in germanium. *Phys. Rev. B* **49**, 14251-14269 (1994).

54. G. Kresse, J. Hafner, Ab initio molecular dynamics for liquid metals. *Phys. Rev. B* **47**, 558-561 (1993).

55. G. Kresse, J. Furthmüller, Efficiency of ab-initio total energy calculations for metals and semiconductors using a plane-wave basis set. *Comput. Mater. Sci* **6**, 15-50 (1996).

56. G. Kresse, J. Furthmüller, Efficient iterative schemes for ab initio total-energy calculations using a plane-wave basis set. *Phys. Rev. B* **54**, 11169-11186 (1996).

57. P. H. T. Philipsen *et al.*, BAND2022, SCM, Theoretical Chemistry, Vrije Universiteit, Amsterdam, The Netherlands, URL: http://www.scm.com.

58. G. Wiesenekker, E. J. Baerends, Quadratic integration over the three-dimensional Brillouin zone. *J. Phys.: Condens. Matter* **3**, 6721 (1991).

59. G. te Velde, E. J. Baerends, Precise density-functional method for periodic structures. *Phys. Rev. B* **44**, 7888-7903 (1991).

60. M. Franchini, P. H. T. Philipsen, L. Visscher, The Becke Fuzzy Cells Integration Scheme in the Amsterdam Density Functional Program Suite. *J. Comput. Chem.* **34**, 1819-1827 (2013).

61. M. Franchini, P. H. T. Philipsen, E. van Lenthe, L. Visscher, Accurate Coulomb Potentials for Periodic and Molecular Systems through Density Fitting. *J. Chem. Theory Comput.* **10**, 1994-2004 (2014).

62. E. J. Baerends *et al.*, ADF2022, SCM, Theoretical Chemistry, Vrije Universiteit, Amsterdam, The Netherlands, URL: http://www.scm.com.

63. G. te Velde *et al.*, Chemistry with ADF. *J. Comput. Chem.* **22**, 931-967 (2001).

64. C. Fonseca Guerra, J. G. Snijders, G. te Velde, E. J. Baerends, Towards an order-N DFT method. *Theor. Chem. Acc.* **99**, 391-403 (1998).

65. J. P. Perdew, K. Burke, M. Ernzerhof, Generalized Gradient Approximation Made Simple [Phys. Rev. Lett. 77, 3865 (1996)]. *Phys. Rev. Lett.* **78**, 1396-1396 (1997).

66. J. P. Perdew, K. Burke, M. Ernzerhof, Generalized Gradient Approximation Made Simple. *Phys. Rev. Lett.* **77**, 3865-3868 (1996).

67. A. V. Krukau, O. A. Vydrov, A. F. Izmaylov, G. E. Scuseria, Influence of the exchange screening parameter on the performance of screened hybrid functionals. *J. Chem. Phys.* **125**, (2006).

68. M. Shishkin, M. Marsman, G. Kresse, Accurate Quasiparticle Spectra from Self-Consistent GW Calculations with Vertex Corrections. *Phys. Rev. Lett.* **99**, 246403 (2007).

69. M. Shishkin, G. Kresse, Self-consistent GW calculations for semiconductors and insulators. *Phys. Rev. B* **75**, 235102 (2007).

70. M. Shishkin, G. Kresse, Implementation and performance of the frequency-dependent GW method within the PAW framework. *Phys. Rev. B* **74**, 035101 (2006).

71. M. S. Hybertsen, S. G. Louie, Electron correlation in semiconductors and insulators: Band gaps and quasiparticle energies. *Phys. Rev. B* **34**, 5390-5413 (1986).

72. L. Hedin, New Method for Calculating the One-Particle Green's Function with Application to the Electron-Gas Problem. *Physical Review* **139**, A796-A823 (1965).

73. E. van Lenthe, J. G. Snijders, E. J. Baerends, The zero-order regular approximation for relativistic effects: The effect of spin–orbit coupling in closed shell molecules. *J. Chem. Phys.* **105**, 6505-6516 (1996).

74. P. H. T. Philipsen, E. van Lenthe, J. G. Snijders, E. J. Baerends, Relativistic calculations on the adsorption of CO on the (111) surfaces of Ni, Pd, and Pt within the zeroth-order regular approximation. *Phys. Rev. B* **56**, 13556-13562 (1997).

75. E. v. Lenthe, E. J. Baerends, J. G. Snijders, Relativistic regular two-component Hamiltonians. *J. Chem. Phys.* **99**, 4597-4610 (1993).
76. G. Kresse, D. Joubert, From ultrasoft pseudopotentials to the projector augmented-wave method. *Phys. Rev. B* **59**, 1758-1775 (1999).
77. P. E. Blöchl, Projector augmented-wave method. *Phys. Rev. B* **50**, 17953-17979 (1994).
78. S. Steiner, S. Khmelevskyi, M. Marsmann, G. Kresse, Calculation of the magnetic anisotropy with projected-augmented-wave methodology and the case study of disordered $Fe_{1-x}Co_x$ alloys. *Phys. Rev. B* **93**, 224425 (2016).
79. E. Van Lenthe, E. J. Baerends, Optimized Slater-type basis sets for the elements 1–118. *J. Comput. Chem.* **24**, 1142-1156 (2003).
80. D. P. Chong, E. Van Lenthe, S. Van Gisbergen, E. J. Baerends, Even-Tempered Slater-Type orbitals revisited: From hydrogen to krypton. *J. Comput. Chem.* **25**, 1030-1036 (2004).
81. V. Wang, N. Xu, J.-C. Liu, G. Tang, W.-T. Geng, VASPKIT: A user-friendly interface facilitating high-throughput computing and analysis using VASP code. *Comput. Phys. Commun.* **267**, 108033 (2021).
82. G. Pizzi *et al.*, Wannier90 as a community code: new features and applications. *J. Phys.: Condens. Matter* **32**, 165902 (2020).
83. C. Ertural, S. Steinberg, R. Dronskowski, Development of a robust tool to extract Mulliken and Löwdin charges from plane waves and its application to solid-state materials. *RSC Adv.* **9**, 29821-29830 (2019).
84. P. C. Müller, C. Ertural, J. Hempelmann, R. Dronskowski, Crystal Orbital Bond Index: Covalent Bond Orders in Solids. *J. Phys. Chem. C* **125**, 7959-7970 (2021).
85. R. Dronskowski, P. E. Bloechl, Crystal orbital Hamilton populations (COHP): energy-resolved visualization of chemical bonding in solids based on density-functional calculations. *The Journal of Physical Chemistry* **97**, 8617-8624 (1993).
86. V. L. Deringer, A. L. Tchougréeff, R. Dronskowski, Crystal Orbital Hamilton Population (COHP) Analysis As Projected from Plane-Wave Basis Sets. *J. Phys. Chem. A* **115**, 5461-5466 (2011).
87. S. Maintz, V. L. Deringer, A. L. Tchougréeff, R. Dronskowski, LOBSTER: A tool to extract chemical bonding from plane-wave based DFT. *J. Comput. Chem.* **37**, 1030-1035 (2016).
88. S. Maintz, V. L. Deringer, A. L. Tchougréeff, R. Dronskowski, Analytic projection from plane-wave and PAW wavefunctions and application to chemical-bonding analysis in solids. *J. Comput. Chem.* **34**, 2557-2567 (2013).
89. K. Momma, F. Izumi, VESTA 3 for three-dimensional visualization of crystal, volumetric and morphology data. *J. Appl. Crystallogr.* **44**, 1272-1276 (2011).
90. J. Su *et al.*, Energy-Degeneracy-Driven Covalency in Actinide Bonding. *J. Amer. Chem. Soc.* **140**, 17977-17984 (2018).
91. W. R. Wadt, Why uranyl ion(2+) is linear and isoelectronic thorium dioxide is bent. *J. Amer. Chem. Soc.* **103**, 6053-6057 (1981).
92. H. L. Schläfer, G. Gliemann. *Einführung in die Ligandenfeldtheorie*, sect. B-V. (Akad. Verlagsgesellschaft Frankfurt am Main, 1967).
93. M. Atanasov, C. Daul, H. U. Güdel, T. A. Wesolowski, M. Zbiri, Ground States, Excited States, and Metal−Ligand Bonding in Rare Earth Hexachloro Complexes: A DFT-Based Ligand Field Study. *Inorg. Chem.* **44**, 2954-2963 (2005).
94. J. S. Griffith, *The theory of transition-metal ions*. (Cambridge university press, 1961).
95. M. J. Reisfeld, G. A. Crosby, Analysis of the Absorption Spectrum of Cesium Uranium (V) Hexafluoride. *Inorg. Chem.* **4**, 65-70 (1965).
96. W. W. Lukens *et al.*, Quantifying the σ and π Interactions between U(V) f Orbitals and Halide, Alkyl, Alkoxide, Amide and Ketimide Ligands. *J. Amer. Chem. Soc.* **135**, 10742-10754 (2013).
97. P. Pyykkö, Y. Zhao, The large range of uranyl bond lengths: ab initio calculations on simple uranium-oxygen clusters. *Inorg. Chem.* **30**, 3787-3788 (1991).
